# Host–guest selectivity in a series of isoreticular metal–organic frameworks: observation of acetylene-to-alkyne and carbon dioxide-to-amide interactions†
†Electronic supplementary information (ESI) available: Correspondence and requests for materials should be addressed to S. Y. and M. S. CCDC 1857732–1857743. For ESI and crystallographic data in CIF or other electronic format see DOI: 10.1039/c8sc03622e


**DOI:** 10.1039/c8sc03622e

**Published:** 2018-10-12

**Authors:** Jack D. Humby, Oguarabau Benson, Gemma L. Smith, Stephen P. Argent, Ivan da Silva, Yongqiang Cheng, Svemir Rudić, Pascal Manuel, Mark D. Frogley, Gianfelice Cinque, Lucy K. Saunders, Iñigo J. Vitórica-Yrezábal, George F. S. Whitehead, Timothy L. Easun, William Lewis, Alexander J. Blake, Anibal J. Ramirez-Cuesta, Sihai Yang, Martin Schröder

**Affiliations:** a School of Chemistry , University of Manchester , Oxford Road , Manchester , M13 9PL , UK . Email: Sihai.Yang@manchester.ac.uk ; Email: M.Schroder@manchester.ac.uk; b School of Chemistry , University of Nottingham , Nottingham , NG7 2RD , UK; c Department of Chemistry , University of Warwick , CV4 7AL , UK; d ISIS Facility , STFC Rutherford Appleton Laboratory , Oxfordshire OX11 0QX , UK; e Oak Ridge National Laboratory , Oak Ridge , TN 37831 , USA; f Diamond Light Source , Harwell Science and Innovation Campus , Oxfordshire , OX11 0DE , UK; g School of Chemistry , Cardiff University , Cardiff CF10 3XQ , UK

## Abstract

In order to develop new porous materials for applications in gas separations such as natural gas upgrading, landfill gas processing and acetylene purification it is vital to gain understanding of host–substrate interactions at a molecular level. Herein we report a series of six isoreticular metal–organic frameworks (MOFs) for selective gas adsorption. These materials do not incorporate open metal sites and thus provide an excellent platform to investigate the effect of the incorporation of ligand functionality *via* amide and alkyne groups on substrate binding. By reducing the length of the linker in our previously reported MFM-136, we report much improved CO_2_/CH_4_ (50 : 50) and CO_2_/N_2_ (15 : 85) selectivity values of 20.2 and 65.4, respectively (1 bar and 273 K), in the new amide-decorated MOF, MFM-126. The CO_2_ separation performance of MFM-126 has been confirmed by dynamic breakthrough experiments. *In situ* inelastic neutron scattering and synchrotron FT-IR microspectroscopy were employed to elucidate dynamic interactions of adsorbed CO_2_ molecules within MFM-126. Upon changing the functionality to an alkyne group in MFM-127, the CO_2_ uptake decreases but the C_2_H_2_ uptake increases by 68%, leading to excellent C_2_H_2_/CO_2_ and C_2_H_2_/CH_4_ selectivities of 3.7 and 21.2, respectively. Neutron powder diffraction enabled the direct observation of the preferred binding domains in MFM-126 and MFM-127, and, to the best of our knowledge, we report the first example of acetylene binding to an alkyne moiety in a porous material, with over 50% of the acetylene observed within MFM-127 displaying interactions (<4 Å) with the alkyne functionality of the framework.

## Abstract 2

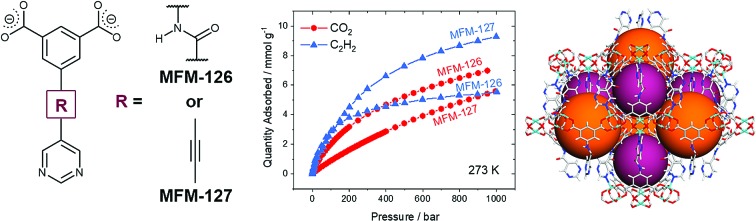
We report a series of six isoreticular metal–organic frameworks (MOFs) for selective gas adsorption, specifically for selective adsorption of CO_2_ and C_2_H_2_.

## Introduction

Porous metal–organic frameworks (MOFs) have attracted considerable attention due to their promise in a wide range of applications, notably in gas adsorption, selectivity and catalysis.^1^–^6^ Modulation of organic linkers and metal nodes allows fine-tuning of properties to tailor MOFs for the application of interest. In the field of gas adsorption, MOFs have shown advantages for selective uptake of specific guests, such as CO_2_ (ref. 7–9) and C_2_H_2_.^10^–^12^ Capture and separation of CO_2_ from flue gas streams *via* the utilization of selective and regenerable sorbents, such as MOFs, provides a promising alternative pathway to conventional amine-scrubbers.^13^ Moreover, MOF adsorbents also have applications in natural gas (containing ∼15% CO_2_) upgrading, and the selective separation of landfill gas (40–60% CO_2_).^14^,^15^ Other industrially significant separations include the purification of C_2_H_2_, an important feedstock in the petrochemical industry. C_2_H_2_ is produced by the partial combustion of CH_4_ and hence requires separation from CH_4_ and CO_2_ to obtain high-purity C_2_H_2_.^16^ However, the identical kinetic diameters of 3.30 Å and similar boiling points of –84 °C and –79 °C, for C_2_H_2_ and CO_2_, respectively, make C_2_H_2_/CO_2_ separation under ambient conditions a highly challenging task.^17^–^19^

Common strategies for enhancing host–guest interactions in MOFs include incorporation of open metal sites,^20^ polar functional groups (*e.g.*, –NH_2_, –CONH–, –OH, –F)^17^,^21^–^24^ and narrowing pore channels by use of small ligands.^25^ For example, polar nitrogen-containing groups remain a favored approach to enhancing CO_2_ adsorption, as shown in a crystallographic study that visualized CO_2_ molecules directly interacting with the amine group in a Zn–MOF.^26^ However, we have previously reported neutron diffraction and scattering data revealing that the high CO_2_ uptake in amide-functionalized MFM-136 is not solely due to guest–host interactions at the amide group, but is a combination of geometry, pore size and functionality that lead to improved gas sorption properties.^27^ Whilst many MOFs have been reported for their gas sorption properties, it is often difficult to fully account for differences in performance owing to many variables such as surface area, porosity and pore geometry, functionality and presence of open metal sites. Thus, to aid design-based approaches for improved materials, thorough investigations of isoreticular series of MOFs such as the IRMOF,^28^ UiO-66 (ref. 29) and MOF-74 (ref. 30) series are important.

Herein we present a comprehensive investigation into the roles of functional groups, pore geometry and porosity in enhancing selective gas binding through a series of six isostructural MOFs (MFM-126–128 and MFM-136–138; Table 1). MFM-137 and MFM-138 were designed by adapting previously reported MFM-136,^27^ replacing the amide group with an ethynyl bond and phenyl ring, respectively. Further modification of MFM-136–138 was achieved by removal of the central phenyl unit of the linker to produce ‘shortened’ derivatives, MFM-126–128. This systematic approach allows us to isolate either the effect of varying pore size or functionality to rationalize the properties of these materials. Our approach has been to focus on the role of ligand sites for substrate binding and, significantly, we report herein the first example of binding of acetylene to the alkyne groups in a porous material at crystallographic resolution.

**Table 1:** Structures of linkers, coordination environments, cage lengths, BET surface areas and experimentally and calculated pore volumes for MFM-126–128 and MFM-136–138. Colors: C, grey; H, white; O, red; N, blue; Cu, teal

	MFM-126	MFM-127	MFM-128	MFM-136^*a*^	MFM-137	MFM-138
Structures of linkers	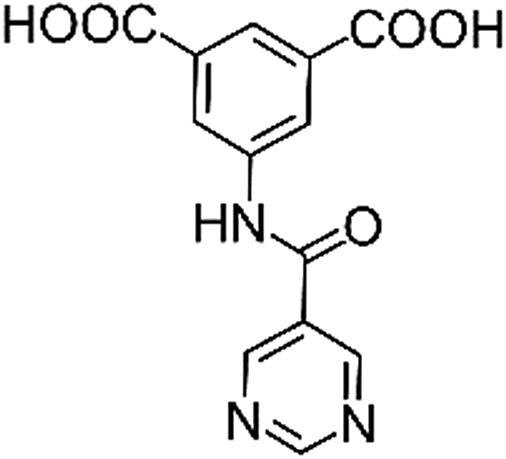	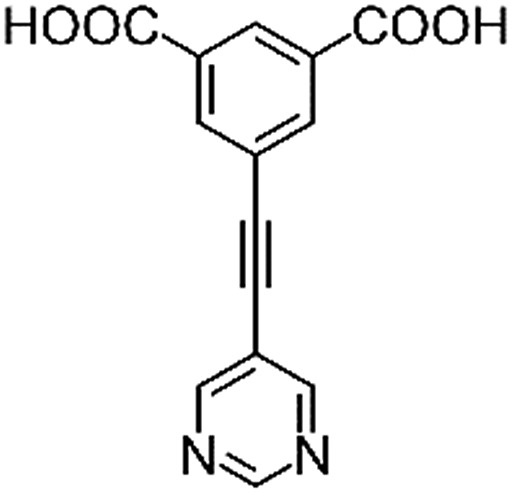	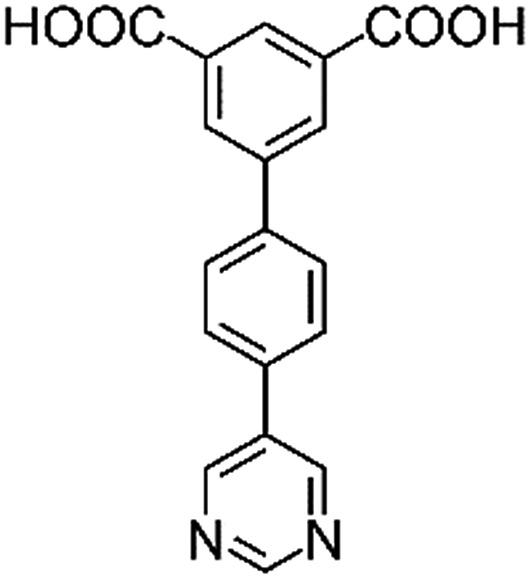	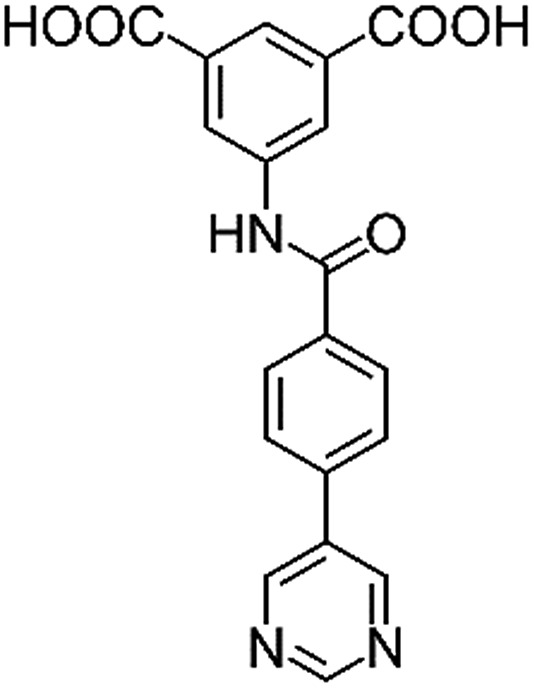	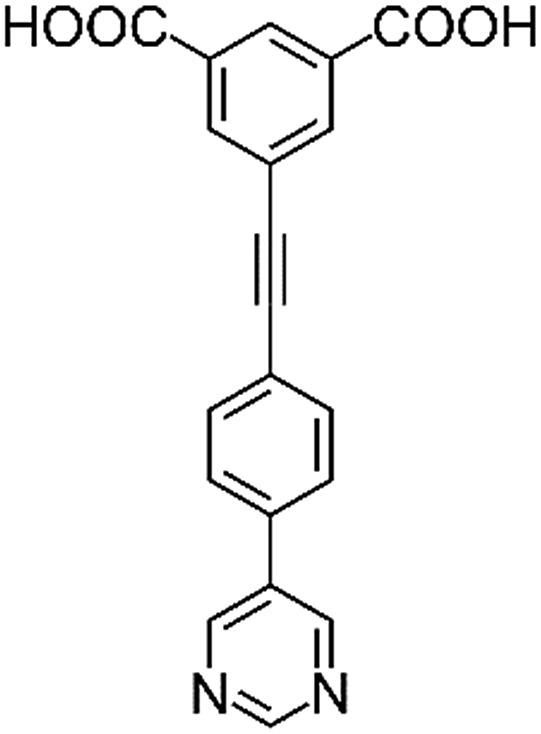	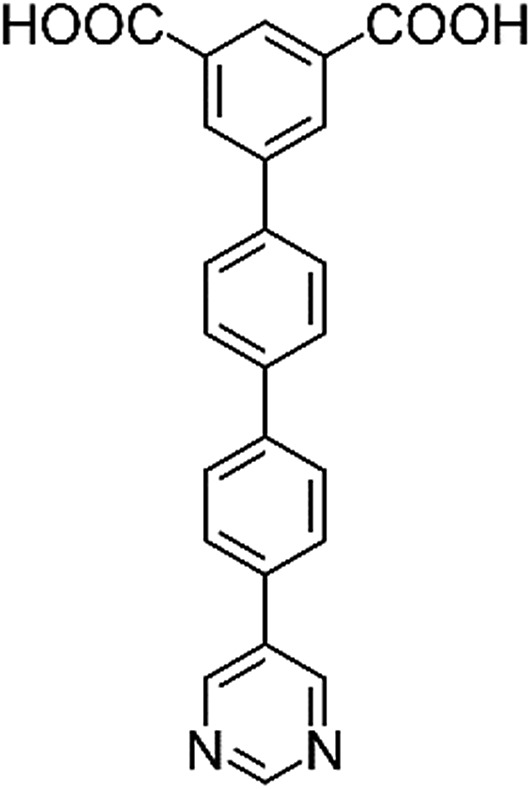
	H_2_L^1^	H_2_L^2^	H_2_L^3^	H_2_L^4^	H_2_L^5^	H_2_L^6^
Coordination environment	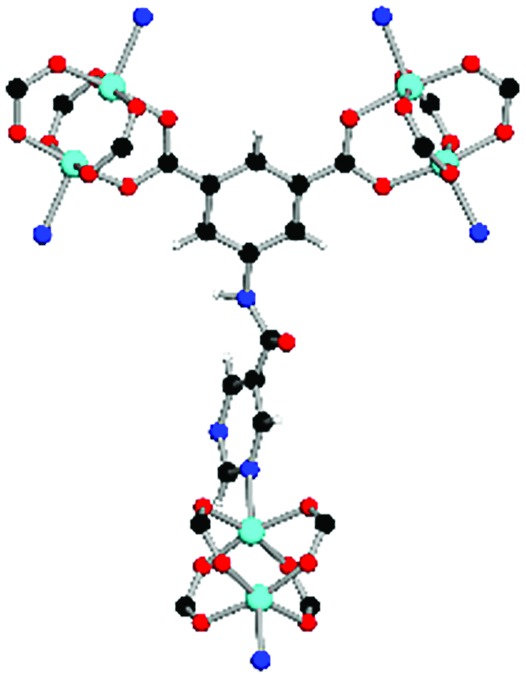	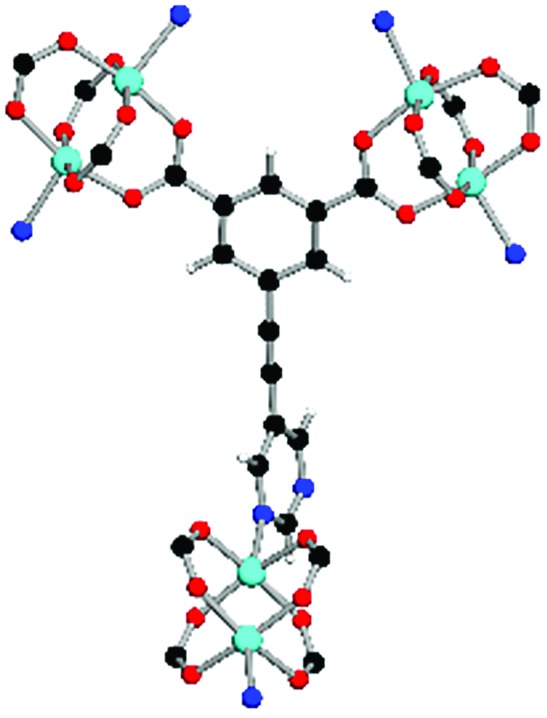	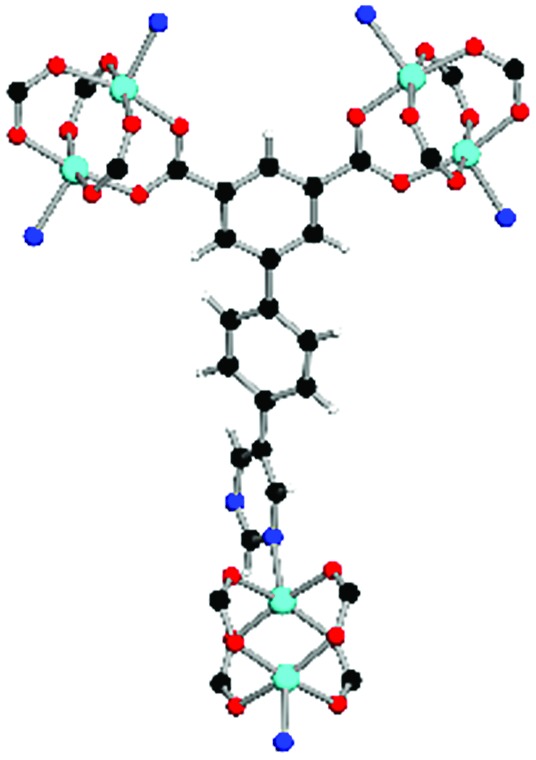	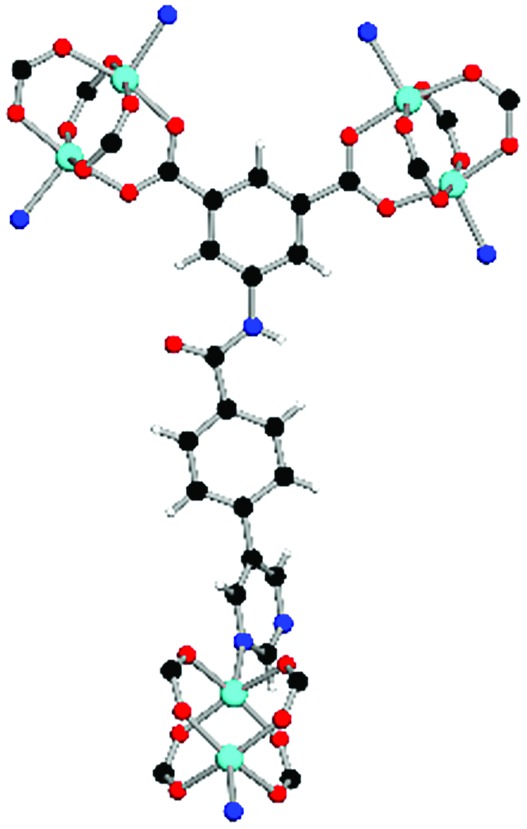	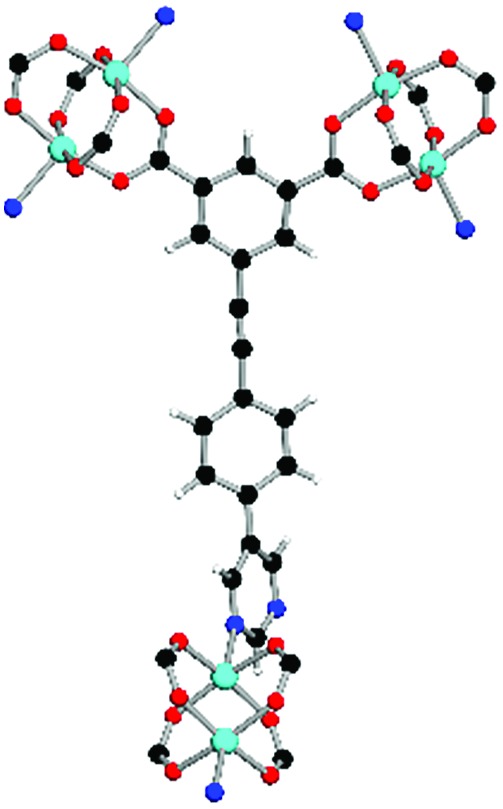	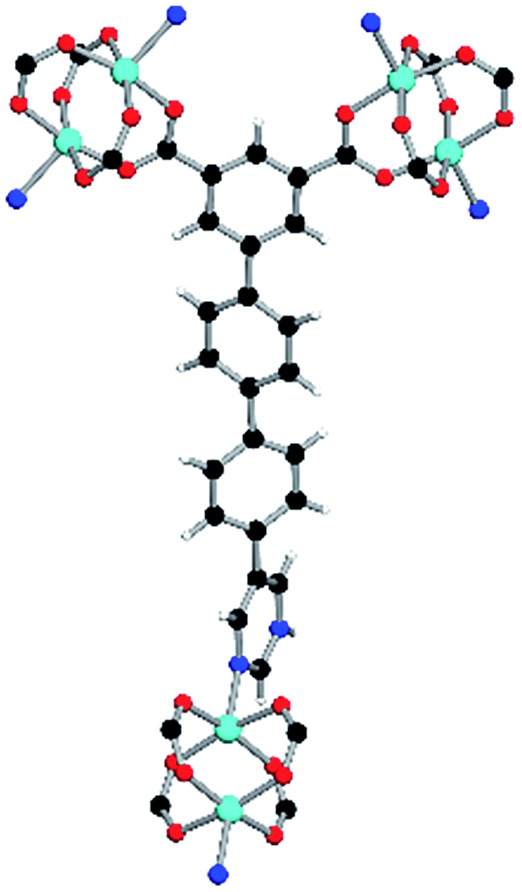
Cage sizes	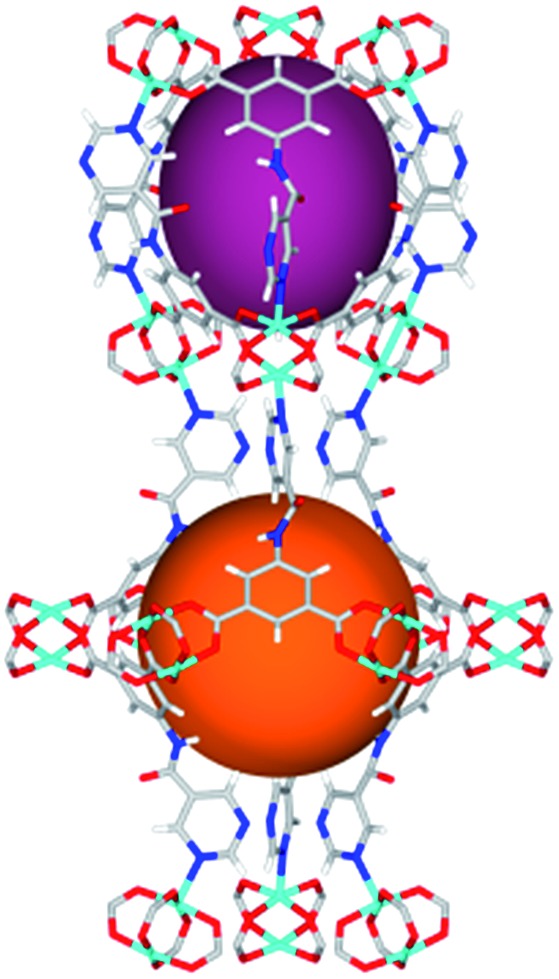	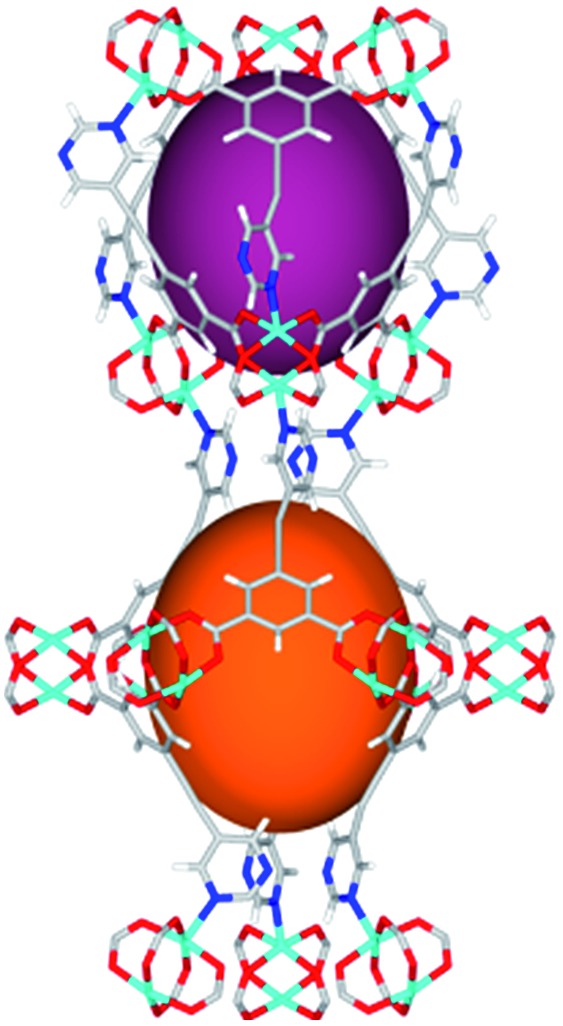	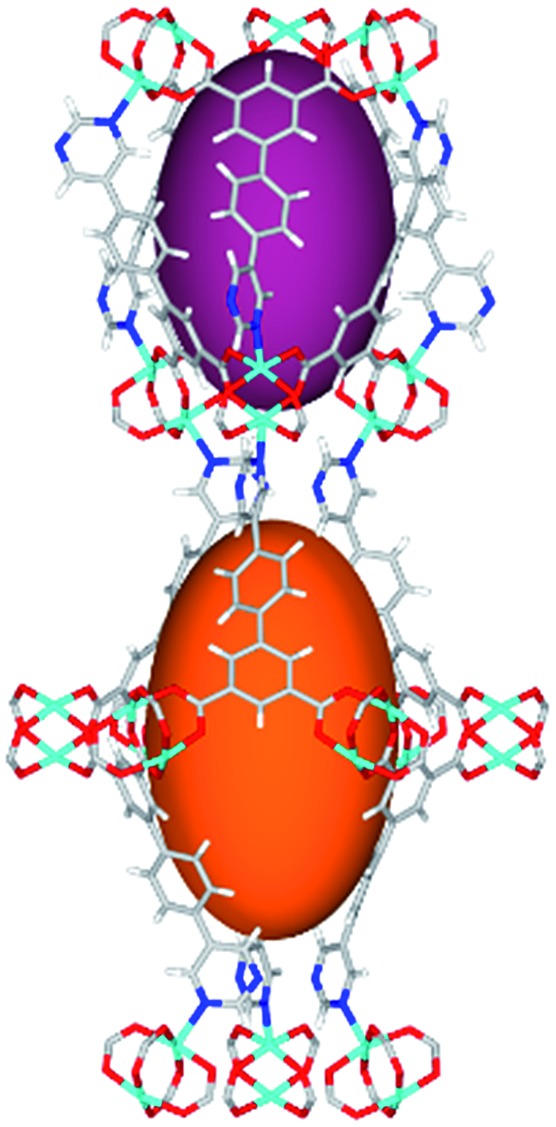	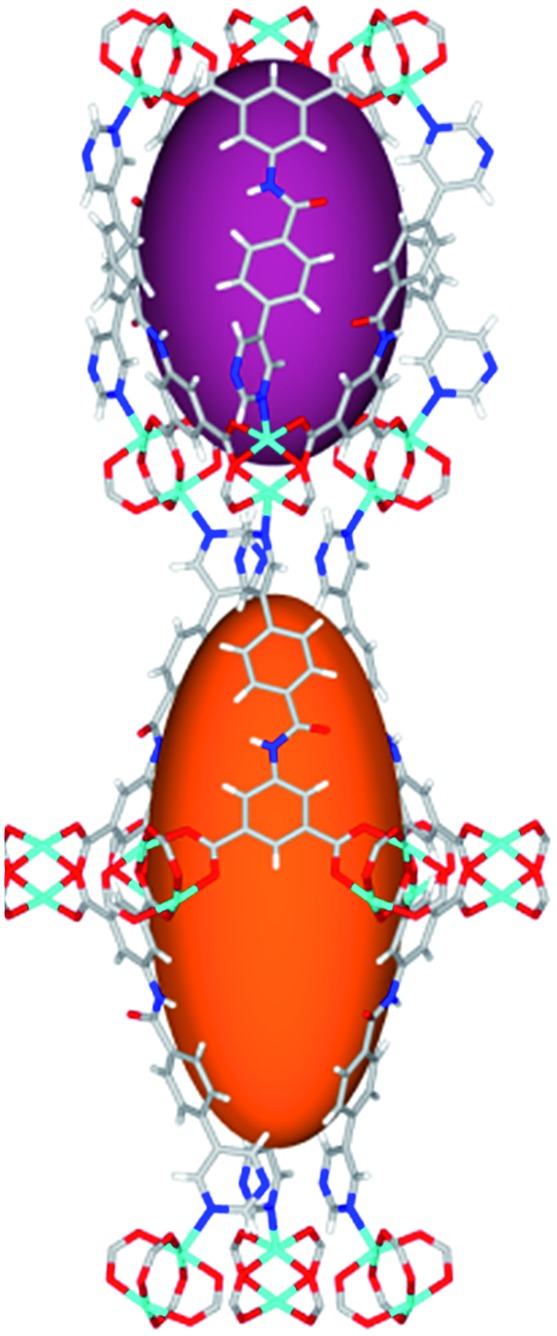	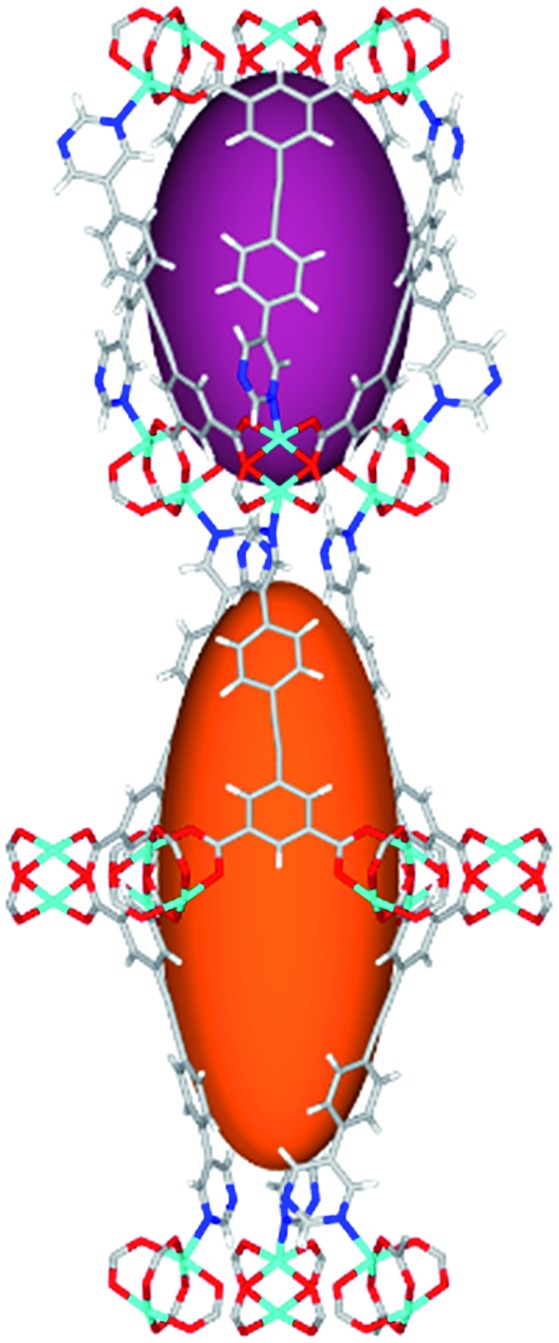	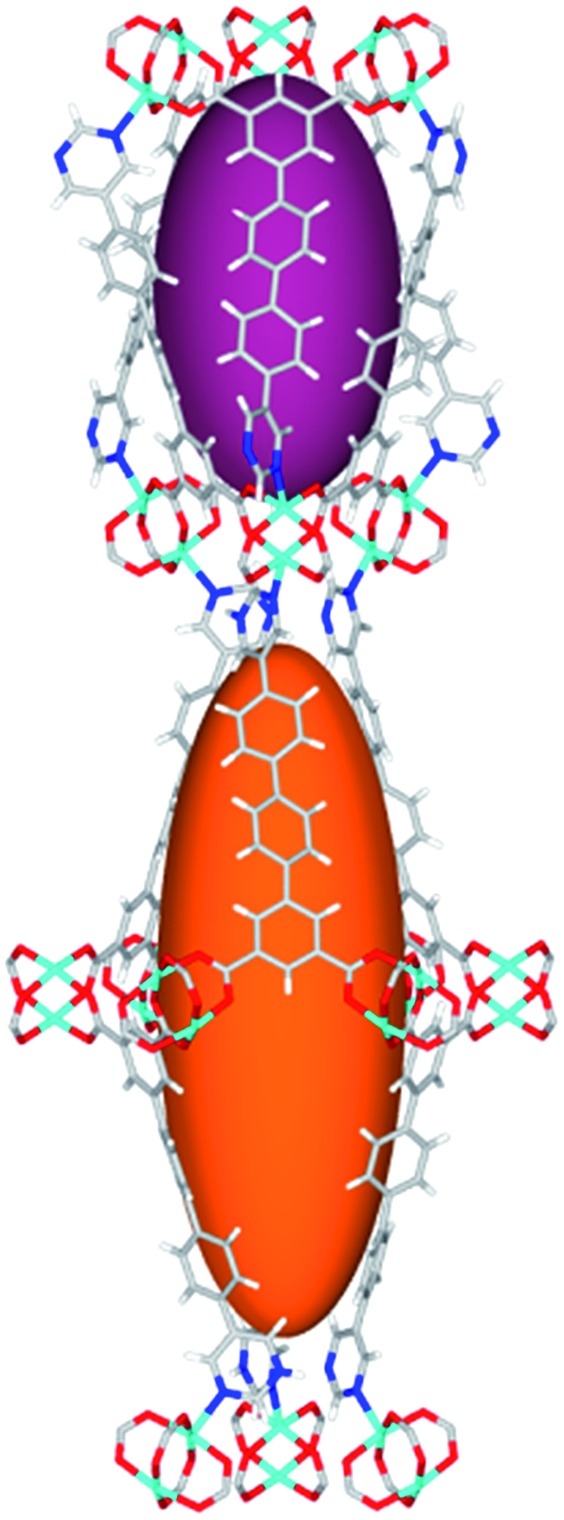
Long cage (**A**) length/Å	15.4	16.4	20.2	24.9	26.1	29.9
Short cage (**B**) length/Å	12.3	12.7	14.1	16.2	16.4	18.1
BET surface area/m^2^ g^–1^	1004	1557	1491	1634	1749	1590
Pore volume (N_2_ isotherm)/cm^3^ g^–1^	0.47	0.52	0.57	0.65	0.61	0.60
Pore volume (single crystal)/cm^3^ g^–1^	0.52	0.57	0.57	0.64	0.67	0.62

^*a*^Previously reported.^27^

## Experimental

### Neutron powder diffraction (NPD) of gas-loaded MFM-126 and MFM-127

NPD data were collected at WISH beamline at ISIS Muon and Neutron facility. Acetone-exchanged MFM-126 and MFM-127 were loaded into 11 mm diameter vanadium cans, heated at 393 K and degassed at 1 × 10^–7^ mbar for 2 days to activate the sample. Data for the bare framework were collected after placing the sample can into a liquid helium cryostat and cooling to 7 K. CO_2_, C_2_D_2_ and CD_4_ gases were dosed into the system after warming the system to 250 and 150 K. The gases were dosed volumetrically from a calibrated volume and, to ensure gases were fully adsorbed into the sample without condensation elsewhere, the system was cooled to 7 K slowly over 3 h. Structures of gas-loaded MFM-126 and MFM-127 were solved by sequential difference Fourier analyses followed by Rietveld refinements using TOPAS software (see ESI Section 4†).

### Inelastic neutron scattering of CO_2_-loaded MFM-126

INS data were collected on TOSCA beamline at ISIS Muon and Neutron facility. MFM-126 was loaded into an 11 mm diameter vanadium can and outgassed at 10^–7^ mbar at 393 K for 2 days. After placing the sample into a He-cooled cryostat, INS data of the bare framework were collected at 7 K. A loading of 1.0 CO_2_/Cu was dosed volumetrically from a calibrated volume at room temperature and gradually cooled to 7 K to allow the guest species to fully adsorb into MFM-126. INS data of 1.0 CO_2_/Cu of MFM-126 were collected at 7 K. Experimentally obtained INS data were compared with modelled data obtained *via* density functional theory (DFT) calculations and simulated using OClimax software (see ESI for further details†).

### Synchrotron FTIR microspectroscopy of CO_2_-loaded MFM-126

Single crystals of MFM-126 were loaded onto a ZnSe slide and placed into a Linkam FTIR600 variable temperature gas-tight cell fitted with ZnSe windows. The MOF sample was activated *in situ* under a flow of N_2_ whilst heating the Linkam stage to 413 K for 6 h. Partial pressures of zeolite-dried gases N_2_ and CO_2_ were controlled by varying the volumetric flow of the two gases *via* separate mass flow controllers. FTIR spectra were collected at the B22 MIRIAM beamline at Diamond Light Source using a polarized and highly bright synchrotron IR source connected to a Bruker Hyperion 3000 IR microscope with a 15× objective and MCT detector (liq. N_2_ cooled). Spectra (256 scans) were measured at room temperature with a 20 × 20 μm beam, in the spectral range of 4000–650 cm^–1^ (4 cm^–1^ resolution).

### Gas breakthrough

Breakthrough experiments were performed using a Hiden Isochema Automated Breakthrough Analyzer with integrated mass spectrometer. MFM-126 (950 mg) was packed into a stainless steel column and the sample was activated by heating to 393 K under a flow of He (100 mL min^–1^) overnight. Breakthrough experiments were conducted using gas mixtures of CO_2_/N_2_ (15 : 85) and equimolar CO_2_/CH_4_ which were flowed over MFM-126 at a total flow rate of 10 mL min^–1^ at 298 K and 1.0 bar (see ESI† for further detail).

## Results and discussion

### Structures

**Fig. 1:**
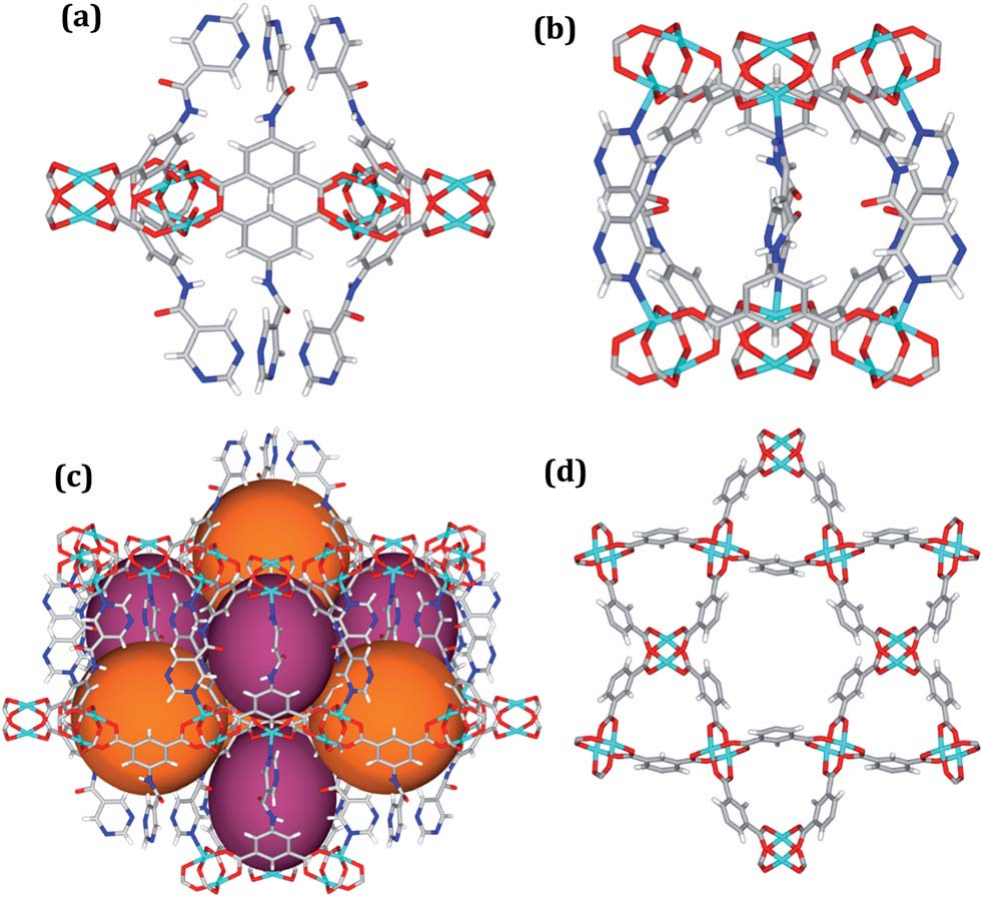
Views of crystal structure of MFM-126. (a) Cage **A**; (b) cage **B**. (c) View of the alternate packing of cages **A** (void space coloured orange) and **B** (void space coloured plum). (d) View along the *c*-axis of the *Kagomé* lattice in MFM-126. Colours: C, grey; H, white; O, red; N, blue; Cu, teal.

Solvothermal reactions of isophthalate–pyrimidyl linkers H_2_L^1^–H_2_L^6^ (Table 1) with Cu(NO_3_)_2_ affords isostructural (3,6)-connected MOFs of *eea* topology. All these frameworks are constructed from Cu(ii) cations bridged by four carboxylate groups from four independent linkers and capped by two pyrimidyl nitrogen donors to form elongated octahedral [Cu_2_(RCOO)_4_(NR)_2_] nodes (Table 1, Fig. 1). The isophthalate units bridge adjacent {Cu_2_} paddlewheels to form a *Kagomé* lattice net (Fig. 1d), and these are connected by coordination of a pyrimidyl nitrogen center to an axial position of the {Cu_2_} paddlewheels. The capping of the {Cu_2_} paddlewheels at both axial positions results in the absence of any open Cu(ii) sites. Lu *et al.* recently reported HHU-2,^31^ a (3,4,6)-connected Cu(ii)-based MOF using H_2_L^1^, but its structure has both pyrimidine N atoms (instead of just one as in MFM-126) bound to Cu(ii) ions and therefore does not have free N centers pointing into the pore as observed in MFM-126 and all MOFs in this current series. MFM-137 and MFM-138 are formed by replacement of the amide group of MFM-136 with alkyne and phenyl moieties, respectively. The removal of a central phenyl ring from MFM-136–138 affords the corresponding shortened derivatives MFM-126–128, respectively.

The MOFs in this series all incorporate two types of cages, the larger of which, cage **A** (Fig. 1a) is comprised of six ligands and six [Cu_2_(RCOO)_4_(NR)_2_] paddlewheel units forming a hexagonal bipyramid. The six {Cu_2_} units form the six equatorial vertices of this cage and the hexagonal window of the *Kagomé* lattice (Fig. 1d). Six pyrimidyl units form the apical vertices, whereby ligands form six of the twelve faces of the hexagonal bipyramid. The smaller cage **B** (Fig. 1b) is constructed from six ligands and six {Cu_2_} paddlewheels forming a ditrigonal scalenohedral cage, whereby two sets of three {Cu_2_} paddlewheels bridged by three linker isophthalate units form triangular windows of neighboring *Kagomé* lattices. The overall structure of these MOFs is comprised of discrete cages **A** and **B**, which are packed in an alternating manner (Fig. 1c), giving highly porous and robust 3D frameworks. Views of the structure along the principal crystallographic axes of MFM-126 are shown in Fig. S3.† The phase purity of all material samples was confirmed by powder X-ray diffraction (PXRD) data (Fig. S4†).

### Modulation of porosity and CO_2_ adsorption properties

All MOFs were solvent-exchanged with acetone or ethanol before heating at 393 K under dynamic vacuum to produce desolvated materials. The N_2_ (77 K) isotherms (Fig. S6†) reveal that the desolvated MOFs have BET surface areas of 1000–1750 m^2^ g^–1^ and pore volumes of 0.47–0.65 cm^3^ g^–1^ (Table 1). As expected, MFM-136–138 have higher BET surface areas compared to their isostructural shorter derivatives MFM-126–128. The higher BET surface area and pore volume of MFM-127 (C≡C linker) compared with MFM-126 (C(O)–NH linker) can be attributed to the presence of the space-efficient alkyne group in the linker, which has been shown previously to increase the hypothetical maximum surface area of MOFs.^32^ The measured pore volumes compare favorably with those calculated directly from single crystal structures, confirming complete desolvation and phase purity of these materials.

**Fig. 2:**
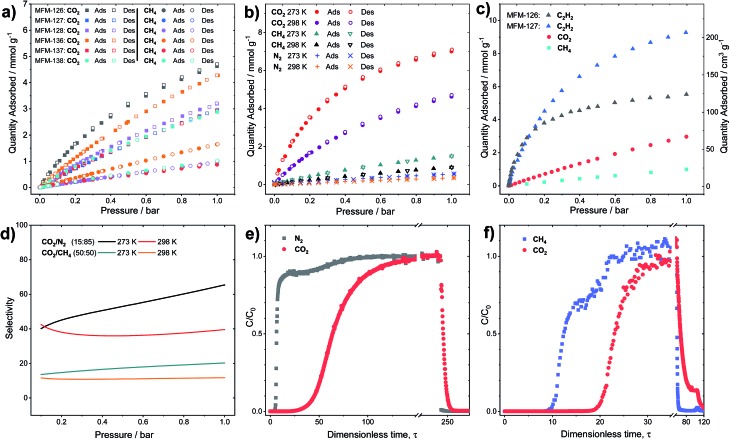
(a) Sorption isotherms for CO_2_ and CH_4_ in MFM-126–128 and MFM-136–138 at 298 K. (b) Sorption isotherms for CO_2_, CH_4_ and N_2_ in MFM-126 at 273 and 298 K. (c) Sorption isotherms for C_2_H_2_, CO_2_ and CH_4_ in MFM-127 and for C_2_H_2_ in MFM-126 at 273 K. (d) IAST selectivity for CO_2_/N_2_ (15 : 85) and equimolar CO_2_/CH_4_ in MFM-126 at 273 and 298 K. (e and f) Experimental breakthrough curves for the adsorption of CO_2_/N_2_ (15 : 85) and equimolar CO_2_/CH_4_ mixtures flowing through a 0.9 mL fixed-bed of MFM-126 at 298 K with a total gas flow of 10 mL min^–1^ at atmospheric pressure. After breakthrough of both species, a He purge was applied leading to desorption of all components.

CO_2_ adsorption isotherms were measured to 20 bar at 273 and 298 K for all MOFs (Fig. S5†). Throughout the series, it was found that the CO_2_ uptake at 20 bar increased with increasing pore volume, indicating that the pore functionality had little effect on high pressure gas adsorption, where porosity is the dominant factor. For example, a 10% increase in CO_2_ uptake at 20 bar is observed in MFM-127 compared with MFM-126 (11.3 and 10.2 mmol g^–1^ at 298 K, respectively), corresponding well with the 11% pore volume increase from MFM-126 to MFM-127. However, at 1.0 bar and 298 K, comparing phenyl-functionalized MFM-138 and MFM-128 (2.89 and 3.19 mmol g^–1^, respectively), it was found that the CO_2_ uptake is greater in MFM-128, indicating a stronger interaction with the MFM-128 framework at ambient pressure. This suggests that pore geometry has an important role in low pressure gas uptake, with MFM-126–128 all having greater uptake at 1.0 bar CO_2_ than the comparative extended derivatives, MFM-136–138 (Fig. 2a). The influence of functionality on CO_2_ uptake can be assessed by comparing MFM-136 and MFM-137 which show similar porosity (Table 1). MFM-136 and MFM-137 show CO_2_ uptakes of 7.30 and 5.76 mmol g^–1^ (273 K), respectively, suggesting that amide-functionalized MFM-136 possesses stronger affinity for CO_2_ compared to the alkyne groups in MFM-137.

Crucially, however, MFM-126, with the smallest pore volume, has the greatest CO_2_ uptake at low pressure across the series, reaching 4.63 mmol g^–1^ at 1 bar and 298 K, presumably due to the greater overlap of attractive interactions between gas molecules and the host framework. This is exemplified further at 0.15 bar, the partial pressure of CO_2_ in flue gas streams, where MFM-126 has a 52% higher uptake of CO_2_ compared with the next best-performing MOF, MFM-136 (2.94 and 1.94 mmol g^–1^, respectively) in this series.

### Selective sorption in MFM-126 and MFM-127

All the MOFs in this series show highly selective uptakes of CO_2_ with respect to CH_4_ and N_2_ (Fig. 2, Table 2). The selective uptake of CO_2_ over the other substrates studied is also evidenced by the analysis of heats of adsorption (Table 2 and Fig. S19†). MFM-126 exhibits the highest CO_2_ uptake at low pressures (<1 bar) as well as relatively low CH_4_ uptake in the same pressure region (Fig. 2b). The selectivity values were estimated from the single-component isotherms using ideal adsorbed solution theory (IAST) (Table 2).^33^ Considering the relative gas concentrations found in natural gas and flue gas streams, MFM-126 exhibits the highest selectivity values across the series for binary mixtures of both CO_2_/CH_4_ (equimolar) and CO_2_/N_2_ (15 : 85 composition) with selectivity values of 20.2 and 65.4, respectively at 1 bar and 273 K (Fig. 2d). MFM-126 also exhibits the highest adsorption enthalpy for CO_2_ of 30.7 kJ mol^–1^, which is significantly higher than 17.3 kJ mol^–1^ for CH_4_ (Table 2). Conversely, MFM-136 has the lowest CO_2_/CH_4_ selectivity value of 4.1 and exhibits similar values for the isosteric heat (*Q*_st_) for CO_2_ and CH_4_ adsorption, 20.1 and 18.9 kJ mol^–1^, respectively. When compared with other leading MOFs, MFM-126 has respectable CO_2_/CH_4_ and CO_2_/N_2_ selectivity values (Tables S18 and S19†).

**Table 2:** Summary of adsorption data for MFM-126–128 and MFM-136–138

MOF	CO_2_ uptake/mmol g^–1^ (1 bar)		CH_4_ uptake/mmol g^–1^ (1 bar)		*S*_CO_2_/CH_4__ (50 : 50, 1 bar)		*S*_CO_2_/N_2__ (15 : 85, 1 bar)		*Q*_st_/kJ mol^–1^ (virial method 1)	
	273 K	298 K	273 K	298 K	273 K	298 K	273 K	298 K	CO_2_	CH_4_
MFM-126	7.00	4.63	1.50	0.897	20.2	11.7	65.4	39.6	30.7	17.3
MFM-127	5.72	2.97	1.71	0.991	5.08	3.33	10.6	7.65	25.8	13.0
MFM-128	5.76	3.20	1.77	0.953	5.46	4.53	35.8	18.9	20.4	22.7
MFM-136	7.29	4.28	2.93	1.64	4.07	3.35	37.0	23.2	20.1	18.9
MFM-137	5.76	2.92	1.41	0.870	6.09	4.08	27.6	15.7	19.2	17.1
MFM-138	6.08	2.89	1.75	1.02	5.42	3.87	17.1	15.5	30.0	18.8

To evaluate the dynamic separation performance of MFM-126, breakthrough experiments were performed with CO_2_/N_2_ (15 : 85) and equimolar CO_2_/CH_4_ mixtures (Fig. 2e and f). The gas mixtures were flowed over a fixed-bed packed with MFM-126 with a total flow of 10 mL min^–1^ at 298 K (1 bar). These breakthrough experiments confirmed the separation potential for both gas mixtures as predicted by IAST selectivity calculations (Fig. 2d).

Although the amide-functionalised MFM-126 exhibits higher CO_2_ uptake compared with MFM-127, the functional groups were also investigated for their effect on acetylene adsorption. Interestingly, alkyne-functionalised MFM-127 exhibits a 68% higher acetylene uptake than MFM-126 at 273 K and 1 bar (9.28 and 5.54 mmol g^–1^, respectively, Fig. 2c). IAST analysis for MFM-127 reveals selectivity values for equimolar mixtures of C_2_H_2_/CO_2_ and C_2_H_2_/CH_4_ to be 3.7 and 21.2, respectively (Fig. S20†). This result is comparable to that observed for [Cu_2_(EBTC)] (H_4_EBTC = 1,1′-ethynebenzene-3,3′,5,5′-tetracarboxylic acid),^34^ which shows enhanced acetylene uptake compared with the non-alkyne bridged analogue MOF-505/MFM-100.^35^,^36^ Whilst the presence of C≡C···C≡C interactions between acetylene gas and an alkyne organic linker may be postulated, this has not yet been confirmed or observed experimentally.

### *In situ* neutron powder diffraction (NPD) of gas-loaded MFM-126 and MFM-127

The absence of open metal sites in this series of MOFs affords an excellent platform to study the role of functional group on gas binding. *In situ* neutron powder diffraction (NPD) has been applied to determine the preferred binding sites of CO_2_ and CD_4_ in MFM-126 and MFM-127 at loadings of 1.2 CO_2_/Cu and 1.0 CD_4_/Cu, and C_2_D_2_ in MFM-127 at a loading of 1.0 C_2_D_2_/Cu. NPD data for the desolvated samples confirm the complete removal of guest solvents and an absence of structural distortion in the parent solvated material. Relatively low loadings of 1.0–1.2 adsorbate/Cu were used to assess the strongest binding sites within the material without involving notable adsorbate–adsorbate interactions. These loadings represent typically 25–40% of the saturated capacities of each adsorbate. Fourier difference map analysis of the NPD patterns afforded the location of guest CO_2_, CD_4_ and C_2_D_2_ molecules which, after further development by Rietveld refinement, allowed unambiguous determination of gas positions, orientations and crystallographic occupancies within each sample.

**Fig. 3:**
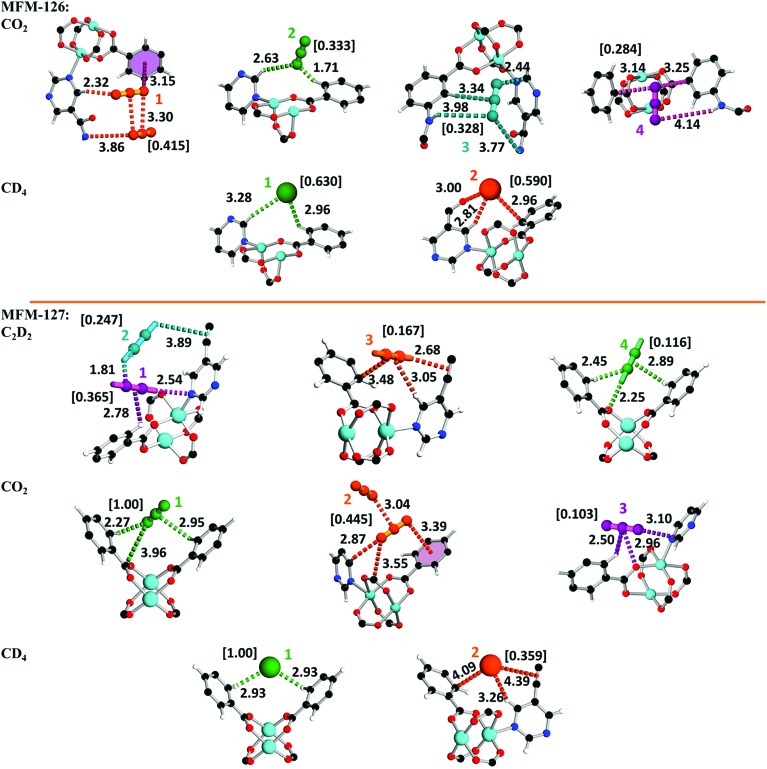
Binding sites of guests in MFM-126 and MFM-127 derived from Rietveld refinement of NPD data with quoted distances in Å. Colors: C, black; H, white; O, red; N, blue; Cu, teal. Refined occupancies of guest molecules are shown in square brackets.

MFM-126 displays four binding sites for CO_2_, **1–4** (in decreasing order of occupancy; Fig. 3). CO_2_-**1** is situated in small cage **B** and exhibits co-operative binding between crystallographically equivalent CO_2_ molecules [C_CO_2__···O_CO_2__ = 3.30(3) Å] where the linear bodies of the CO_2_ molecules lie parallel with an interaction to an adjacent amide [O_CO_2__···N_amide_ = 3.86(5) Å, <C=O···N = 111°]. In addition, there are short contacts between a pyrimidyl ring [C–H_pyrimidine_···O_CO_2__ = 2.32(5) Å, <C–H···O = 143°] and an isophthalate ring [O_CO_2__···centroid_isophthal._ = 3.15(4) Å, <C=O···centroid = 111°]. CO_2_-**2** is located in the triangular window of cage **B**, with a close contact to a C–H group of the isophthalate unit [O_CO_2__···H–C_isophthal._ = 1.71(10) Å, <O···H–C = 152°] and a side-on interaction with two adjacent pyrimidine rings [O_CO_2__···H–C_pyrimidine_ = 2.17(5) Å, <O···H–C = 128°]. CO_2_-**3** is situated in a pocket between cages **A** and **B** with two short contacts to amido N–H units [O_CO_2__···H–N_amide_ = 3.77(6) Å, <O···H–N = 124° and O_CO_2__···H–N_amide_ = 3.98(8) Å, <O···H–N = 138°]. Further to this, there are two other close contacts with a pyrimidyl ring [O_CO_2__···C_pyrimidine_ = 2.44(5) Å, C=O···C = 115°] and a side-on interaction with an isophthalate ring [C_CO_2__···H–C_isophthal._ = 3.34(12) Å, <C···H–C = 172°]. CO_2_-**4** is positioned at the periphery of larger cage **A** with a hydrogen bond to an amido N–H [O_CO_2__···H–N = 4.14(9) Å, <O···H–N = 149°] as well as lying in a crevice between two isophthalate units [O_CO_2__···C_isophthal._ = 3.14(11) Å, C=O···C = 81° and O_CO_2__···C_isophthal._ = 3.25(10) Å, C=O···C = 96°].

Analysis of CD_4_-loaded MFM-126 reveals seven sites **1–7**, the first two of which have significantly higher crystallographic occupancies of 0.630 and 0.590 than the rest (Fig. 3). CD_4_-**1**, resides in cage **B** in an equivalent site to that of CO_2_-**2**, also with close interactions to isophthalate and pyrimidyl rings [D_4_C···H–C_isophthal._ = 2.96(11) Å, <C···H–C = 122° and D_4_C···H–C_pyrimidine_ = 3.28(4) Å, <C···H–C = 159°]. CD_4_-**2** is found in an identical site to that of CO_2_-**1** located in the center of cage **B** with two key interactions to an isophthalate ring and an amido carbonyl of the framework [D_4_C···H–C_isophthal._ = 2.81(4) Å, <C···H–C = 146° and D_4_C···O=C_amide_ = 3.00(5) Å, <C···O=C = 140°].

Refinement of NPD data of C_2_D_2_-loaded MFM-127 unveiled five sites, **1–5** (Fig. 3). Interestingly, over 50% of C_2_D_2_ were found having short contacts (<4 Å) to the framework alkyne units. C_2_D_2_-**1** is situated in a narrow window between cages **A** and **B**, with closest interactions to pyrimidyl and isophthalate rings [DC_2_–D···N_pyrimidine_ = 2.54(5) Å, <C–D···N_pyrimidine_ = 145° and η^2^-D_2_C_2_···H–C_isophthal._ = 2.78(2) Å, <C–H···C≡C_centroid_ = 108°]. Cooperative binding is visualized between C_2_D_2_-**1** and C_2_D_2_-**2** [**1**-D_2_C≡C···D–C_2_D-**2** = 1.81(3) Å, <C^1^···D–C^2^ = 127°]. C_2_D_2_-**2** also binds to the framework alkyne [DC_2_–D···η^2^-C≡C_framework_ = 3.89(5) Å, <C–D···C≡C_centroid_ = 123°] suggesting the framework alkyne has an integral role in the uptake of acetylene in MFM-127. C_2_D_2_-**3** is located in the center of cage **B** with H-bonds to a framework alkyne moiety [DC_2_–D···η^2^-C≡C_framework_ = 2.68(5) Å, <C–D···C≡C_centroid_ = 120°] as well as close contacts to pyrimidyl and isophthalate rings [η^2^-D_2_C≡C···H–C_pyrimidine_ = 3.05(8) Å, <C–H···C≡C_centroid_ = 130° and η^2^-C≡CD_2_···C_isophthal._ = 3.48(3) Å]. C_2_D_2_-**4** occupies the hydrophobic triangular window of cage **B** with strong binding to an oxygen of the {Cu_2_} paddlewheel [DC_2_–D···O_paddlewheel_ = 2.25(5) Å, <C–D···O = 150°] as well as to adjacent isophthalate rings [D_2_C_2_···H–C_isophthal._ = 2.45(2) Å, <C–H···C = 120° and η^2^-C≡CD_2_···H–C_isophthal._ = 2.89(1) Å, <C–H···C≡C_centroid_ = 124°].

Three CO_2_ binding domains, **1–3**, were found in MFM-127 (Fig. 3). CO_2_-**1** is situated in an equivalent site to C_2_D_2_-**4** with a close contact to a carboxylate C^*δ*+^ [O_CO_2__···C_COO_ = 3.96(6) Å, <C–O···C = 100°] as well as side-on interactions with isophthalate rings [O_CO_2__···H–C_isophthal._ = 2.27(1) Å, <C–H···O = 135° and C_CO_2__···H–C_isophthal._ = 2.95(7) Å, <C–H···C = 114°]. CO_2_-**2** occupies the corresponding site to CO_2_-**1** in MFM-126. As for MFM-126, cooperative binding is exhibited between crystallographically equivalent CO_2_-**2** molecules in cage **B** of MFM-127 [C_CO_2__···O_CO_2__ = 3.04(2) Å, <C=O···C = 128°]. Crucially, these results reveal the key role of the amide groups on selective adsorption of CO_2_ in MFM-126 as well as confirming that alkyne moieties do not act as effective adsorption sites for CO_2_ in MFM-127.

NPD data for CD_4_-loaded MFM-127 exposed only two noteworthy sites of interaction. Both sites correspond directly to CD_4_ sites **1** and **2** observed in MFM-126, respectively. In MFM-127, CD_4_-**1** resides in the hydrophobic triangular windows of cage **B** [D_4_C···H–C_isophthal._ = 2.93(1) Å, <C–H···C = 120°], further showing that this pocket provides an optimal environment for CD_4_. CD_4_-**2** is found in the center of cage **B** and interacts weakly to the framework alkyne [D_4_C···η^2^-C≡C_framework_ = 4.39(1) Å] revealing that the alkyne moiety has little effect on CD_4_ adsorption.

Thus, significantly, the NPD study reveals that it is a combination of cooperative binding as well as the amide functionality that leads to enhanced interaction of CO_2_ with MFM-126. On the other hand, NPD data reveal that alkyne-functionalized MFM-127 exhibits much weaker interaction with CO_2_, but that the alkyne moieties play critical roles in acetylene binding, with over half of the adsorbed acetylene molecules exhibiting interactions (<4 Å) to alkyne moieties in the pore of MFM-127. This represents the first example of direct visualization of acetylene binding to an alkyne moiety in porous materials.

### Inelastic neutron scattering (INS) studies of CO_2_-loaded MFM-126

To gain further understanding into CO_2_ binding in MFM-126, inelastic neutron scattering (INS) was measured as a function of CO_2_-loading (Fig. S18†). The INS spectrum for the bare MOF exhibits numerous vibrational modes which have been assigned by comparison with a DFT-calculated INS spectrum (Fig. S17†). Interestingly, unlike previously reported with isostructural MFM-136,^27^ MFM-126 exhibits significant interactions with guest CO_2_. Upon CO_2_ loading, there is a shift in the peak at 82.8 meV (assigned to the in-plane bending of the amide group) to 84.1 meV signifying an interaction between adsorbed CO_2_ and the amide group, such as those visualized in the structure model of CO_2_-loaded MFM-126. This result contrasts with that of MFM-136 (ref. 27) and confirms the importance of cooperativity between functional groups (in this case amide) and the pore geometries on guest binding.

### *In situ* synchrotron FT-IR microspectroscopy of MFM-126

**Fig. 4:**
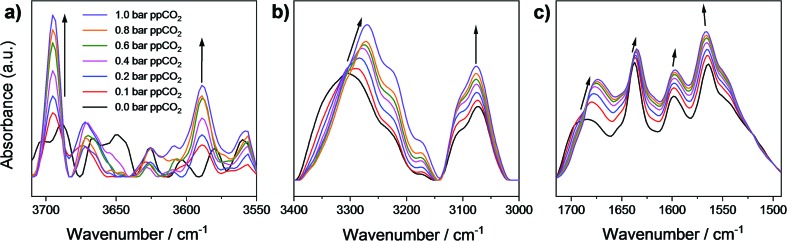
Micro-FTIR spectra of a single crystal of MFM-126 as a function of CO_2_-loading incrementally from 0 to 1 bar ppCO_2_ focusing on (a) the CO_2_ combination bands at 3695 and 3590 cm^–1^; (b) *ν*(N–H) stretching band shifting from 3306 cm^–1^ at 0 bar ppCO_2_ (in N_2_) of CO_2_ (in N_2_) to 3270 cm^–1^ at 1 bar ppCO_2_; (c) the *ν*(N–H) bending mode shifting from 1684 cm^–1^ at 0 bar ppCO_2_ (in N_2_) to 1674 cm^–1^ at 1 bar ppCO_2_. All spectra were recorded referenced to the blank cell as a function of CO_2_ loading to remove contributions of free gaseous CO_2_.

To investigate further the nature of host–guest interactions, *in situ* synchrotron FT-IR microspectroscopy was conducted on a single crystal of activated MFM-126. FTIR spectra were collected as a function of CO_2_-loading by increasing the partial pressure of CO_2_ in N_2_ from 0 to 1.0 bar (ppCO_2_; Fig. 4). The *ν*_1_ + *ν*_3_ (3695 cm^–1^) and *2ν*_2_ + *ν*_3_ (3590 cm^–1^) combination bands of CO_2_ (Fig. 4a) were used to monitor CO_2_ sorption as the fundamental antisymmetric stretch at ∼2348 cm^–1^ saturates at ppCO_2_ above 0.2 bar. These combination bands are red-shifted from their free values (3714 and 3612 cm^–1^, respectively) at all partial pressures of CO_2_ loading and increase in intensity as a function of ppCO_2_, confirming that CO_2_ interacts strongly with the framework (Fig. 4a). Activated MFM-126 has a *ν*(N–H) stretch at 3306 cm^–1^ and upon increasing ppCO_2_ from 0 to 1 bar, this band shifts by 36 cm^–1^ to 3270 cm^–1^. In addition, the observed shift of the amide *ν*(C=O) stretching band by 10 cm^–1^ (from 1684 cm^–1^ in the absence of CO_2_ to 1674 cm^–1^ at 1.0 bar ppCO_2_; Fig. 4c) further indicates CO_2_ adsorption directed by the amide group in MFM-126. Overall, these observations are highly consistent with the NPD and INS results.

## Conclusions

A series of isoreticular (3,6)-connected pyrimidyl isophthalate Cu(ii)-based MOFs, of rare *eea* topology have been synthesized and characterized. Although MFM-136 exhibits the highest CO_2_ uptake at 20 bar, it was found that upon shortening the linker unit to form MFM-126, selectivity of CO_2_ uptake at 1 bar increases dramatically. The relative ease of synthesis of MFM-126 coupled with its high selectivity for binary mixtures of CO_2_/CH_4_ and CO_2_/N_2_, position this MOF as a viable candidate for CO_2_ separations. Furthermore, the CO_2_ separation performance of MFM-126 has been confirmed by dynamic breakthrough experiments. NPD data of CO_2_-loaded MFM-126 has revealed that cooperative binding of CO_2_ (at position CO_2_-**1**) and binding to amide groups that decorate cage **B** both have strong effects on the observed selective CO_2_ binding. The *in situ* spectroscopic studies using INS and FTIR also establish that adsorbed CO_2_ interacts strongly with the amide groups of MFM-126, with significant shifts of amide (C=O and N–H) vibrational bands on CO_2_ loading. Replacing the amide-group in MFM-126 with an alkyne-group to give MFM-127 leads to a decrease in both CO_2_ and CH_4_ uptake capacities relative to MFM-126, but affords a 68% greater C_2_H_2_ capacity than MFM-126. NPD experiments reveal for the first time that acetylene interacts directly with alkyne moieties of MFM-127 in the pore. Notably, over 50% of the acetylene observed within MFM-127 displays strong interactions (<4 Å) with the alkyne functionality of the framework. The understanding gained in this study provides further insights into the development of materials showing improved gas binding *via* specific interaction to ligand sites within the MOF.

## Conflicts of interest

The authors declare no competing financial interests.

## Supplementary Material

Supplementary information

Crystal structure data
