# Supplementary material for: Host–guest selectivity in a series of isoreticular metal–organic frameworks: observation of acetylene-to-alkyne and carbon dioxide-to-amide interactions
Source: Chem Sci. 2018 Oct 12;10(4):1098–106. doi: 10.1039/c8sc03622e (PMC6346404; doi:10.1039/c8sc03622e)
Supplement: Supplementary file 1 [file SC-010-C8SC03622E-s001.pdf]

Electronic Supplementary Material (ESI) for Chemical Science.
This journal is © The Royal Society of Chemistry 2018

Supplementary Information

Host-guest selectivity in a series of isoreticular metal-organic frameworks: observation of acetylene-to-alkyne and carbon dioxide-to-amide interactions

Jack D. Humby,[1a] Oguarabau Benson,[2a] Gemma L. Smith,[1] Stephen P. Argent,[3] Ivan da Silva,[4] Yongqiang Cheng,[5] Svemir Rudić,[4] Pascal Manuel,[4] Mark D. Frogley,[6] Gianfelice Cinque,[6] Lucy K. Saunders,[6] Iñigo J. Vitórica-Yrezábal,[1] George F. S. Whitehead,[1] Timothy L. Easun,[7] William Lewis,[2] Alexander J. Blake,[2] Anibal J. Ramirez-Cuesta,[4] Sihai Yang*[1] and Martin Schröder*[1]

1. School of Chemistry, University of Manchester, Manchester, M13 9PL, UK.
   Sihai.Yang@manchester.ac.uk; M.Schroder@manchester.ac.uk.
2. School of Chemistry, University of Nottingham, Nottingham, NG7 2RD, UK.
3. Department of Chemistry, University of Warwick, CV4 7AL, UK.
4. ISIS Facility, STFC Rutherford Appleton Laboratory, Oxfordshire OX11 0QX, UK.
5. Oak Ridge National Laboratory, Oak Ridge, TN 37831, USA.
6. Diamond Light Source, Harwell Science and Innovation Campus, Oxfordshire, OX11 0DE, UK.
7. School of Chemistry, Cardiff University, Cardiff CF10 3XQ, UK.

a. these authors contributed equally to this work.

Contents:

1. Synthesis and Characterisation

2. X-ray Crystallography

3. Gas Sorption

4. Neutron Powder Diffraction Studies

5. Inelastic Neutron Spectroscopy

6. Calculation of Isosteric Heats of Adsorption

7. Selectivity Calculations

8. Breakthrough Experiments

1. Synthesis and Characterisation

All chemical reagents and gases were obtained from commercial sources and used as received. ^{1}H and ^{13}C NMR were measured on Bruker DPX 300, Bruker AV400, or Bruker AV(III)500 spectrometers. Residual protonated species in the deuterated solvents were used as internal references. Mass spectrometry was performed on a Bruker MicroTOF with the sample dissolved in methanol or acetonitrile. Elemental analyses were measured on a CE-440 Elemental Analyzer.

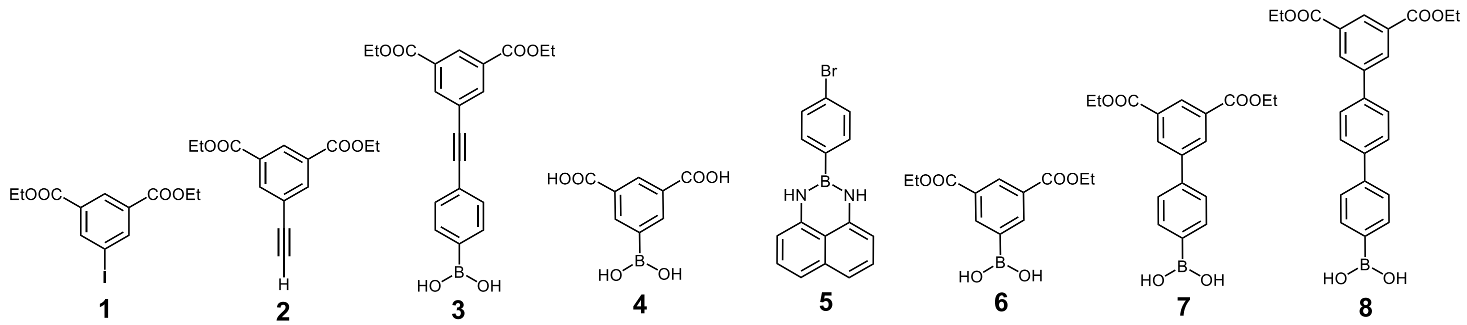

Scheme S1. Target starting materials, 1–8.

Preparation of diethyl 5-iodoisophthalate (1)

A solution of $NaNO_2$ (4.62 g, 64.9 mmol) in H_2O (30 mL) was added to a cold suspension of diethyl 5-aminoisophthalate (12.84 g, 54.1 mmol, obtained by esterification of 5-aminoisophthalic acid with EtOH) in 2M HCl (80 mL) and the reaction mixture stirred for 1 h. An ice-cold aqueous solution of KI (13.48 g, 81.2 mmol, 1.5 eq) in H_2O (100 mL) was added dropwise at 0°C and CH_2Cl_2 (50 mL) added to aid solubility. The reaction mixture was stirred overnight at ambient temperature and quenched with a saturated aqueous solution of $Na_2S_2O_3$ (100 mL). The organic layer was extracted with CH_2Cl_2 (3 x 50 mL) washed with brine (50 mL) and dried over Na_2SO_4. The solvent was removed *in vacuo* to give a brown oil/solid, which was purified by column

chromatography using CH_2Cl_2 as eluent to yield **1** (12.50 g, 66 % yield). ^{1}H NMR (400 MHz, DMSO-*d*$_6$) δ (ppm) 8.39 (d, *J* = 1.51 Hz, 2H, isoph-H), 8.35 (t, *J* = 1.63 Hz, 1H, isoph-H), 4.34 (q, *J* = 7.07 Hz, 4H, CH_2), 1.34 (t, *J* = 7.09 Hz, 6H, CH_3); ^{13}C NMR (101 MHz, DMSO-*d*$_6$) δ (ppm) 164.02, 141.90, 132.56, 129.02, 95.56, 62.06, 14.51; MS (ESI): *m/z* = calc. 348.9931, found 348.9946 (M + H).

Preparation of diethyl 5-ethynylisophthalate (2)

A solution of **1** (5.00 g, 14.4 mmol) in a mixture of Et_3N (50 mL) and THF (50 mL) was degassed under Ar. $Pd(PPh_3)_4$ (0.45 g, 2 mol%) and CuI (0.08 g, 3 mol%) were added followed by ethynyltrimethylsilane (2.6 mL, 17.3 mmol) and left to stir at ambient temperature for 24 h before being quenched by the addition of aqueous saturated NH_4Cl solution (150 mL). The organic layer was extracted with CH_2Cl_2 (3 x 100 mL), dried over $MgSO_4$, filtered through silica to yield a brown solution which was stirred with Cs_2CO_3 (5.64 g, 17.3 mmol) in EtOH (25 mL) and H_2O (25 mL) at ambient temperature for 4 h. The mixture was extracted with CH_2Cl_2 (2 x 30 mL), dried over $MgSO_4$ and the solvent removed *in vacuo*. The solid was purified by column chromatography using CH_2Cl_2 as eluent to yield target compound **2** as a yellow solid (2.72 g, 77 % yield).^{1}H NMR (400 MHz, chloroform-*d*) δ (ppm) 8.65 (t, *J* = 1.63 Hz, 1H, isoph-H), 8.33 (d, *J* = 1.63 Hz, 2H, isoph-H), 4.43 (q, *J* = 7.15 Hz, 6H, CH_2), 3.20 (s, 2H, B(OH)), 1.43 (t, *J* = 7.09 Hz, 6H, CH_3); ^{13}C NMR (101 MHz, chloroform-*d*) δ (ppm) 165.00, 136.89, 131.31, 130.62, 123.03, 81.70, 79.04, 61.60, 14.29; MS (ESI): *m/z* = calc. 247.0965, found 247.0960 (M + H), calc. 269.0784, found 269.0778 (M+Na).

Preparation of (4-((3,5-bis(ethoxycarbonyl)phenyl)ethynyl)phenyl) boronic acid (3)

$Pd(PPh_3)_4$ (0.50 g, 1.6 mmol) and CuI (0.03 g, 0.3 mmol) were added to a degassed solution of **2** (1.12 g, 4.5 mmol) and 2-(4-bromophenyl)-2,3-dihydro-1H-naphtho[1,8-*de*][1,3,2]diazaborinine (1.45 g, 4.5 mmol) in THF (30 mL) and diisopropylamine (15 mL). The temperature of the mixture was raised to 50 ^oC and stirred for 48 h, and the reaction was cooled and quenched by the addition of aqueous saturated NH_4Cl solution (50 mL). The reaction was extracted with CH_2Cl_2 (3 x 30 mL) and dried over $MgSO_4$. The solvent was removed *in vacuo* to give a solid that was washed with MeOH. The solid which was de-esterified by stirring at 60 ^oC in THF (100 mL) and 2M H_2SO_4 (25 mL). After reducing the solvent volume by half, water was added to precipitate the product which was isolated by filtration, washed with water and dried to give **3** (0.64 g, 39 % yield).^{1}H NMR (400 MHz, DMSO-*d*$_6$) δ (ppm) 8.41 (t, *J* = 1.57 Hz, 1H, isoph-H), 8.26 (d, *J* = 1.51 Hz, 2H, isoph-H), 8.23 (s, 2H, B(OH)), 7.84 (d, *J* = 7.90 Hz, 2H, Ar-H), 7.59 (d, *J* = 8.70 Hz, 2H, Ar-H), 4.37 (q, *J* = 7.11 Hz, 4H, CH_2), 1.35 (t, *J* = 7.09 Hz, 4H, CH_3);^{13}C NMR (101 MHz, DMSO-*d*$_6$) δ (ppm) 164.69, 136.07, 134.74, 131.67, 131.04, 124.12, 123.36 , 92.07, 67.49, 62.06, 30.88, 25.59, 14.53; MS (ESI): *m/z* = calc. 367.1347, found 367.1268 (M + H), calc. 389.1167, found 389.1171 (M + Na), calc. 365.1202, found 365.1208 (M-H).

Preparation of 5-boronoisopthalic acid (4)

3,5-Dimethylphenylboronic acid (15.00 g, 95.0 mmol) and NaOH (15.00 g, 375 mmol) were dissolved in *tert*-butanol:water (600 mL, 1:1 v/v). The mixture was heated to 50 °C and $KMnO_4$ (82 g, 1 mol) added in 2 to 4 g portions over five days. The temperature was increased to 65°C after two-thirds had been added. Excess permanganate was reduced by addition of $Na_2S_2O_3$ (ca. 5.0 g) and the precipitated MnO_2 removed by filtration and washed with boiling water. The solvent volume was reduced *in vacuo* and the product precipitated by addition of conc. HCl to pH 2. The resulting solid was isolated by filtration, washed with cold slightly acidic water, recrystallized from hot water and dried to yield **4** (11.02 g, 55 %). ^{1}H NMR (400 MHz, DMSO-d_6) δ (ppm) 13.15 (br. s., 2H, COOH), 8.61 (d, J = 1.88 Hz, 2 H, isoph-H), 8.51 (t, J = 1.76 Hz, 1 H, isoph-H), 8.41 (br. s. 2 H B(OH)); ^{13}C NMR (101 MHz, DMSO-d_6) δ (ppm) 167.48,139.49, 132.05, 130.86; MS: (ESI) *m/z* calc.209.0263; found 209.0261 (M-H).

Preparation of 2-(4-bromophenyl)-2,3-dihydro-1*H*-naphtho[1,8-*de*] [1,3,2]diazaborinine (5)

A mixture of 4-bromophenyl boronic acid (10.00 g, 48.8 mmol) and 1,8-diaminonaphthalene (9.40 g, 58.8 mmol) in toluene under reflux at 140°C using a Dean-Stark apparatus. After 2 h, the reaction was stopped and the solvent evaporated *in vacuo.* The resulting solid was dissolved in $CHCl_3$ and filtered through silica. The filtrate was evaporated to dryness and recrystallized from CH_2Cl_2/petroleum ether (boiling fraction 60-80 °C) to yield 5 as a brown solid (15.04 g, 95 %).^{1}H NMR (400 MHz, DMSO-d_6) δ (ppm) 8.33 (s, 2 H), 7.90 (m, J = 8.28 Hz, 2 H), 7.63−7.68 (m, 2 H), 7.05−7.12 (m, 2 H), 6.89−6.93 (m, 2 H), 6.59 (dd, J = 7.47, 0.82 Hz, 2 H); ^{13}C NMR (101 MHz, DMSO-d_6) δ (ppm) 142.65, 136.40, 135.27, 131.10, 128.12, 124.47, 120.20, 116.86, 106.19; MS: (ESI) *m/z* calc. 367.0262; found 367.0274, (M+CO_2).

Preparation of diethylisophthalate-5-boronic acid (6)

A suspension of **4** (7.00 g, 33.4 mmol) and conc. H_2SO_4 (12 mL) in EtOH (250 mL) were heated under reflux at 100 °C for 22 h. The reaction mixture was filtered when hot and evaporated to half volume. Water was added to precipitate **6** which was isolated by filtration, washed with water and dried (8.62 g, 82 %).^{1}H NMR (400 MHz, DMSO-d_6) δ (ppm) 8.59 (d, J = 1.63 Hz, 2 H, isoph-H), 8.47 (t, J = 1.63 Hz, 1 H, isoph-H), 4.33 (q, J = 7.03 Hz), 1.32 (t, J = 7.09 Hz, isoph-H); ^{13}C NMR (101 MHz, DMSO-d_6) δ (ppm) 165.85, 139.44, 131.56, 130.15, 61.70, 14.56; MS: (ESI) *m/z* calcd 267.1034; found 267.1028, (M+H) calcd 289.0854; found 289.0857 (M+Na).

Preparation of (3',5'-bis(ethoxycarbonyl)-[1,1'-biphenyl]-4-yl)boronic acid (7)

To a degassed solution of **5** (1.50 g, 4.6 mmol), **2** (1.44 g, 5.4 mmol) and K_2CO_3 (1.50 g, 10.6 mmol) in toluene (110 mL) and water (27 mL) was added (^{t}Bu)$_3$P (1.0 mL, 1M solution in toluene, 1.0 mmol) and $Pd_2(dba)_3$ (0.3 g, 0.3 mmol). The reaction mixture heated to 80 °C under reflux for 40 min after which it was quenched with aqueous saturated NH_4Cl solution. The aqueous layer was extracted with CH_2Cl_2 and the solution dried over $MgSO_4$ and filtered through silica before the solvent removed in *vacuo*. The resulting solid was recrystallized from $CHCl_3$ and petroleum ether (boiling fraction 60-80 °C), dried and then heated at 60 °C in THF (90 mL)

and 2M sulphuric acid (15 mL) for 2 h. The product was precipitated with water and then recrystallised from methanol/water to yield **7** (1.08 g, 69 %). ^{1}H NMR (400 MHz, DMSO-d_6) δ (ppm) 8.45 (t, *J* = 1.83 Hz, 1 H, isoph-H) 8.41 (d, *J* = 1.52 Hz, 2 H, isoph-H) 7.94 (d, *J* = 8.07 Hz, 2 H, Ar-H) 7.71 (d, *J* = 8.07 Hz, 2 H) 4.39 (q, *J* = 7.00 Hz, 4 H, Et-H) 1.36 (t, *J* = 7.08 Hz, 6 H, Et-H); ^{13}C NMR (101 MHz, DMSO-d_6) δ (ppm) 165.33, 141.88, 139.89, 135.52, 131.85, 131.77, 128.78, 126.39, 61.86, 55.37, 14.61; MS: (ESI): *m/z* = calc. 343.1347, found 343.1349 (M+H); calc. 365.1167, found 365.1159; (M+Na) calc. 360.1613, found 360.1623 ($M+NH_4$).

Preparation of (3',5'-bis(ethoxycarbonyl)-[1,1'4',1''-terphenyl]-4-yl)-boronic acid (8)

To a degassed solution of **5** (3.00 g, 9.3 mmol), **7** (4.40 g, 12.9 mmol) and K_2CO_3 (4.50 g, 32.6 mmol) in toluene (400 mL) and water (100 mL) was added (^{t}Bu)$_3$P (3.0 mL, 1 M solution in toluene, 3.0 mmol) and $Pd_2(dba)_3$ (0.8 g, 0.8 mmol). The reaction mixture heated to 80 °C under reflux for 30 minutes after which it was quenched with aqueous saturated NH_4Cl solution. The organic layer was extracted with CH_2Cl_2 and the solution dried over $MgSO_4$ before the solvent removed in *vacuo*. The dry solid was deprotected by heating at 60 °C in THF (200 mL) and 2M H_2SO_4 (50 mL) for 2 h and the product precipitated on addition of the product with water. The product was isolated by filtration, washed extensively with water and dried to give **8** (2.30 g, 74%). ^{1}H NMR (400 MHz, DMSO-d_6) δ (ppm) 8.48 (s, 1 H), 8.46 (s, 2 H), 8.13 (s, 2 H), 7.92 (d, *J* = 8.28 Hz, 2 H), 7.87 (s, 4 H), 7.73 (d, *J* = 8.16 Hz, 2 H), 4.41 (q, *J* = 7.11 Hz, 4 H), 1.38 (t, *J*=7.09 Hz, 6 H).

Preparation of Organic Linkers H_2L^1-H_2L^6

Preparation of 5-(pyrimidine-5-carboxamido)isophthalic acid (H_2L^1)

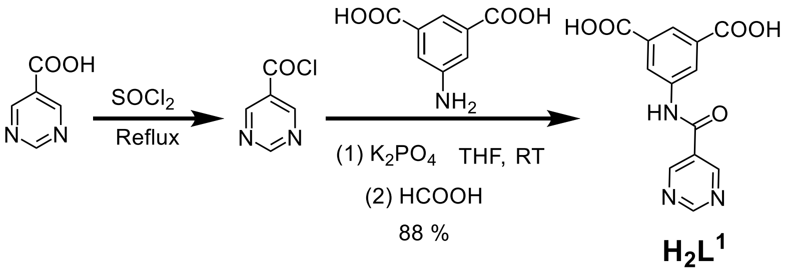

Scheme S2. Synthesis of H_2L^1.

A suspension of 5-pyrimidine carboxylic acid (1.00 g, 7.8 mmol) in thionyl chloride (25 mL) and DMF (0.02 mL) was heated at 80°C under reflux to give a clear solution after 3 h. The solvent evaporated with care under reduced pressure, and the resulting solid was azeotroped with toluene under vacuum to remove traces of residual thionyl chloride. The resulting solid was suspended in anhydrous THF (50 mL) under Ar and then cooled to 0°C. 5-Aminoisophthalic acid (1.44 g, 7.8 mmol) and K_3PO_4 (4.14 g, 19.5 mmol) were added and the reaction mixture stirred as it warmed up to ambient temperature for 2 h. Water (50 mL) was added and some of the solvent (30 mL) removed *in vacuo*. MeCN (30 mL) was added and the solution acidified to pH = 2 using formic acid. The resulting yellow solid was isolated by filtration and washed with water and MeCN and dried to give

H_2L^1 (1.02 g, 46%). ^{1}H NMR (400 MHz, DMSO-d_6): δ (ppm) = 13.31 (br s, 2H, COOH) 10.92 (s, 1H, NH), 9.38 (s, 1H, Pymr-H), 9.31 (s, 2H, Pymr-H), 8.63 (d, J = 1.51 Hz, 2H, Ar-H), 8.25 (t, J = 1.51 Hz, 1H, Ar-H; ^{13}C NMR (100 MHz, DMSO-d_6): δ (ppm) = 166.87, 163.04, 160.80, 156.81, 139.69, 132.35, 128.66, 125.94, 125.10 ; HRMS (ESI): *m/z* = calc. 288.0615, found 288.0612 (M+H), calc. 310.0434, found 310.0430 (M+Na); ATR FT-IR: ν (cm^{-1}) = 1693(m) 1563(w) 1413(m) 1332(w) 1255(m) 1175(w) 909(m) 758(m) 708(w) 656(m) 596(m); Elemental Analysis (Calc. in % for $C_{13}H_9N_3O_5 \cdot H_2O$): C, 51.15; H, 3.63; N, 13.77. Found: C, 51.22; H, 3.25; N, 12.89.

Preparation of 5-(pyrimidin-5-yl) isophthalic acid (H_2L^2)

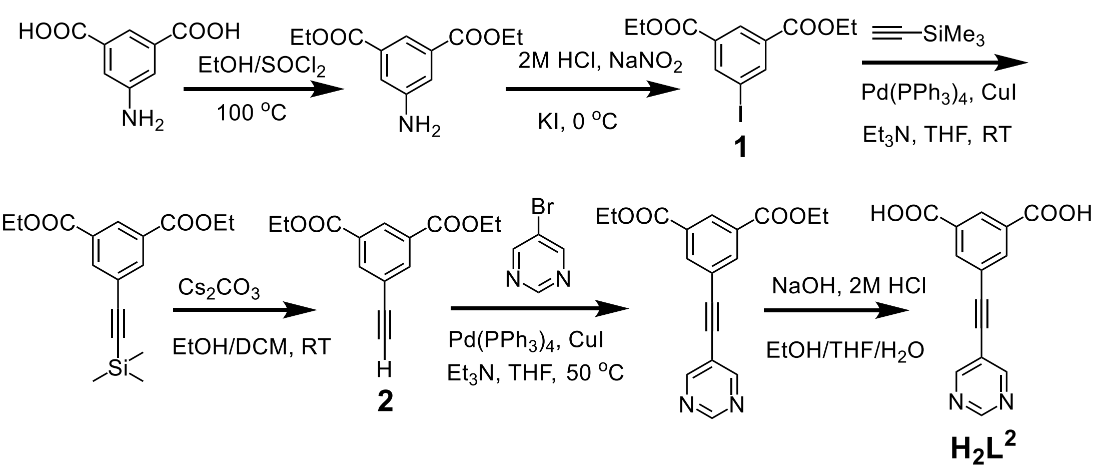

Scheme S3. Synthesis of H_2L^2.

2 (2.72 g, 11.0 mmol) was added to a degassed solution of 5-bromopyrimidine (1.96 g, 12.1 mmol) in THF (70 mL) and Et_3N (35 mL) at ambient temperature. $Pd(PPh_3)_4$ (0.51 g, 3 mol%) and CuI (0.063 g, 3 mol%) were then added and the solution heated to 50°C for 24 h. After quenching with aqueous saturated NH_4Cl solution (100 mL), the organic layer was extracted with CH_2Cl_2 (3 x 30 mL) and dried over $MgSO_4$. The solvent was removed *in vacuo* to give a yellow solid which was purified by column chromatography (CH_2Cl_2: ethyl acetate, 3:2) to give a light-yellow solid which was hydrolysed using NaOH in EtOH and H_2O to yield H_2L^2 (0.94 g, 32%). ^{1}H NMR (400 MHz, DMSO-d_6) δ (ppm) 13.60 (br. s. 2 H, COOH), 9.24 (s, 1H, pymr-H), 9.11 (s, 2H, pymr-H), 8.50 (t, *J* = 1.63 Hz, 1H, Ar-H), 8.33 (d, *J* = 1.63 Hz, 2H, Ar-H); ^{13}C NMR (101 MHz, DMSO-d_6) δ (ppm) 166.19, 159.49, 157.68, 136.21, 132.73, 130.94, 122.79, 118.88, 94.01 , 84.98; ; MS (ESI): *m/z* = calc. 269.0557, found 269.0556 (M + H), calc. 291.0376, found 291.0369 (M + Na); ATR FT-IR: ν (cm^{-1}) 1704(s), 1446(w), 1412(w), 1268(s), 1181(m) 1098(w), 910(w), 756(m), 709(m), 689(w), 644(w), 612(w), 571.10(w); Elemental Analysis [(%), (Calc. for $C_{14}H_8N_2O_4$)]: C, 62.69; H, 3.01; N, 10.44. Found: C, 62.02; H, 3.01; N, 10.27.

Preparation of 4'-(pyrimidin-5-yl)-[1, 1'-biphenyl]-3,5-dicarboxylic acid (H_2L^3)

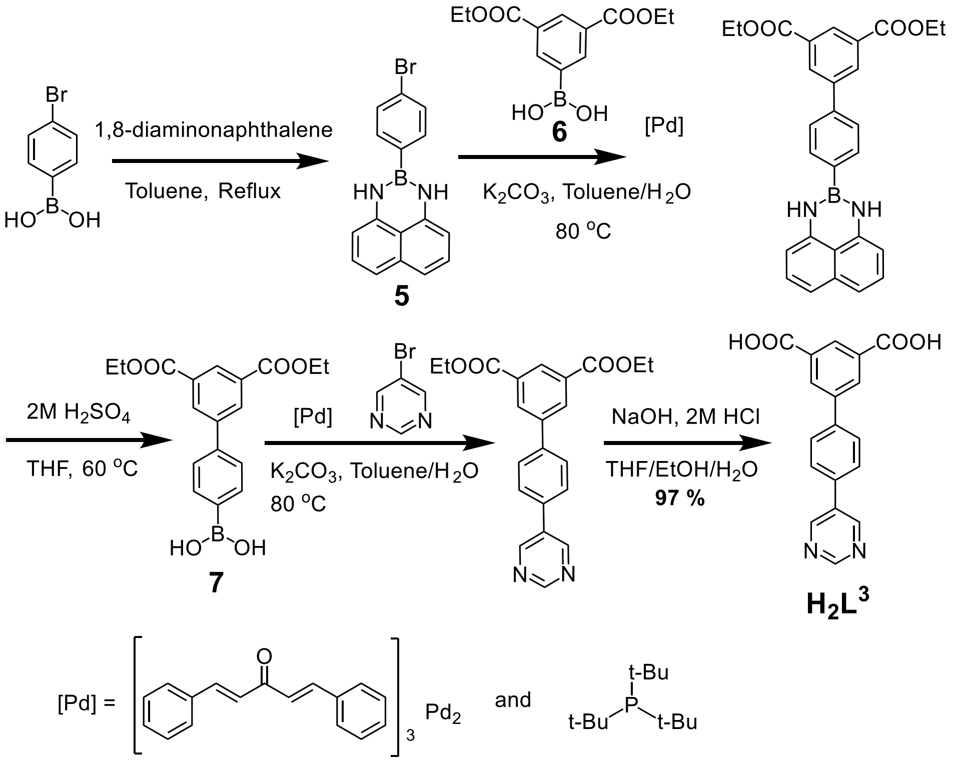

Scheme 4. Synthesis of H_2L^3.

To a degassed solution of **7** (0.50 g, 1.5 mmol), 5-bromopyrimidine (0.24 g, 1.5 mmol) and K_2CO_3 (0.50 g, 3.6 mmol) in toluene:water (100 mL, 4:1 v/v) at 60°C was added $(^tBu)_3P$ (0.5 mL, 1.0 mol solution in toluene, 0.5 mmol) and $Pd_2(dba)_3$ (0.2 g, 0.2 mmol) and the reaction mixture heated to 80°C for 30 min after which it was quenched with aqueous saturated NH_4Cl solution. The organic layer was extracted with CH_2Cl_2, dried over and the solvent removed in *vacuo*. The product was purified by column chromatography (CH_2Cl_2:ethyl acetate, 3:2) and dried to yield the diethyl ester [diethyl 4'-(pyrimidin-5-yl)-[1,1'-biphenyl]-3,5-dicarboxylate (0.36 g, 67%)]. This was hydrolysed using NaOH (0.40 g, 10 mmol) in EtOH:THF:water (36 mL, 1:1:1 v/v) by heating under reflux at 85°C for 5 h. The solvent was reduced to half *in vacuo* and the product was precipitated by addition of conc. HCl to pH 1. The resulting solid was collected by filtration, washed with water and dried to yield target ligand. H_2L^3 (0.30 g, 97%). ^1H NMR (400 MHz, DMSO-d_6): δ (ppm) = 9.22 (s, 2H, Pymr-H), 9.21 (s, 1H, Pymr-H), 8.49--8.48 (t, 1H, Ar-H), 8.44-8.43 (d, 2H, Ar-H), 7.99--7.97 (dd, 2H, Ar-H), 7.93-87.91(dd, 2H, Ar-H); ^{13}C NMR (100 MHz, DMSO-d_6): δ (ppm) = 166.74, 157.68, 154.99, 147.46, 139.08, 133.93, 132.85, 132.47, 132.14, 131.57, 128.10, 128.03; HRMS (ESI): *m/z* = calc. 321.0870, found 321.0851 (M+H); ATR FT-IR: ν (cm^{-1}) = 3360(w), 3122 (m), 3054(m), 2443(w), 2351(w), 2323 (w), 2160(w), 1961(w), 1842 (w), 1704(s), 1589(w), 1557 (w), 1416(s), 1396(w), 1355(w), 1328(m), 1281(w), 1247 (s), 1176 (s), 1138(m), 1127(w), 1069 (m), 1028(m), 1008(s), 919(m), 900(m), 829 (s), 786 (m), 754 (s), 721 (s), 736(w), 673(m), 651(s), 642(m), 633(m); Elemental Analysis (Calc. in % for $C_{18}H_{12}N_2O_4$·$0.5H_2O$): C, 65.85; H, 3.83; N, 8.53. Found: C, 65.77; H, 3.85; N, 8.35.

Preparation of 5-(4-(pyrimidin-5-yl)benzamido)isophthalic acid (H_2L^4)

This was completed by the published procedure.[1]

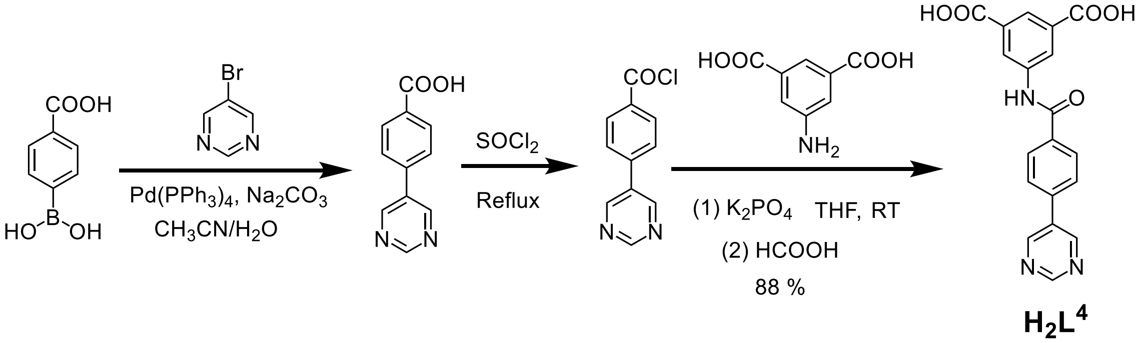

Scheme 5. Synthesis of H_2L^4.

Preparation of 5-((4-(pyrimidin-5-yl)phenyl)ethynyl)isophthalic acid (H_2L^5)

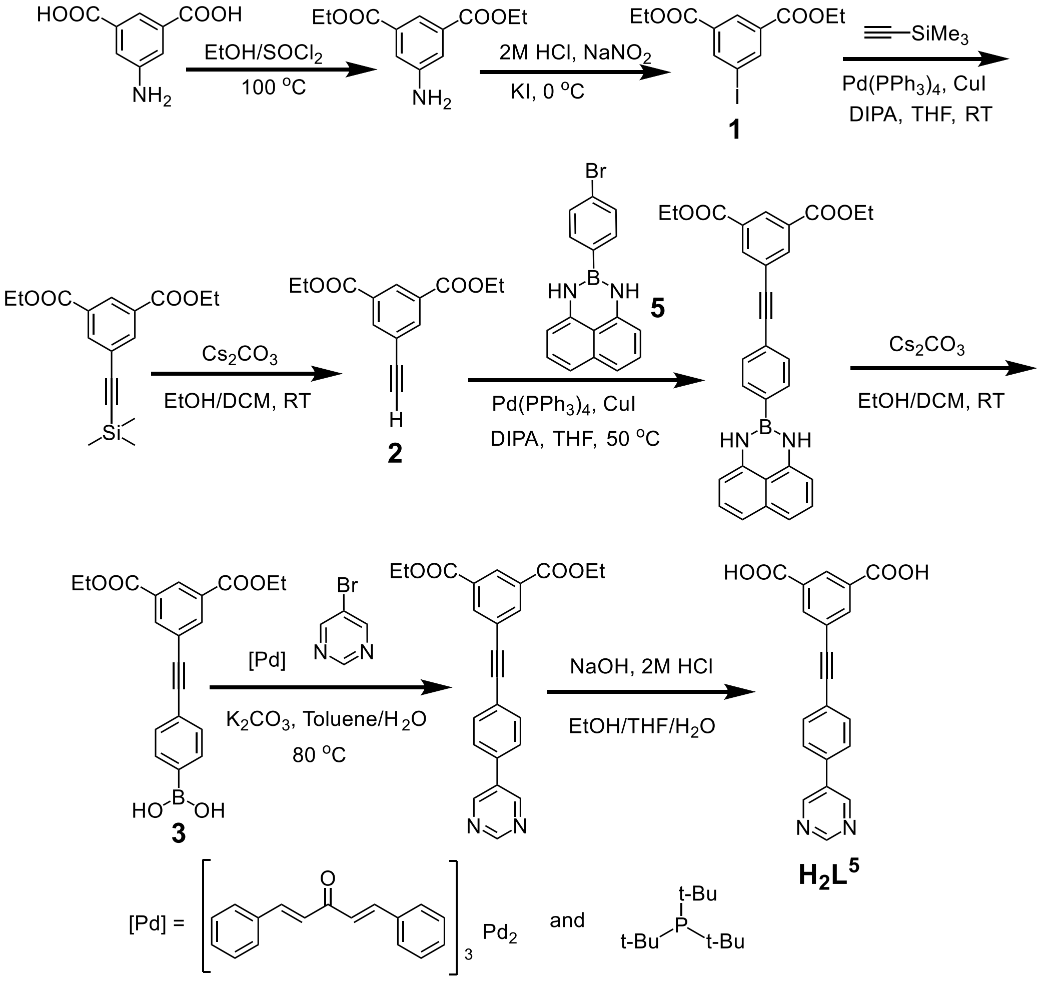

Scheme 6. Synthesis of H_2L^5.

A suspension of **3** (0.64 g, 1.7 mmol) and 5-bromopyrimidine in a mixture of toluene (50 mL) and H_2O (15 mL) was degassed under Ar at 60°C. $P(^tBu)_3$ (0.6 mL, 1.0 mol solution in toluene, 0.6 mmol) and $Pd_2(dba)_3$ (0.2 g, 0.2 mmol) were added and the reaction mixture heated to 80°C under reflux for 40 min after which it was quenched with aqueous saturated NH_4Cl solution (50 mL). The organic layer was extracted with CH_2Cl_2 (50 mL), washed with brine, dried over $MgSO_4$ and the solvent removed *in vacuo*. The obtained solid was washed with MeOH (50 mL) and dried to give an off-white solid, which was hydrolysed using NaOH (0.5 g) in EtOH (20 mL), THF (20 mL) and H2O (20 mL) to yield H_2L^5 (0.29 g, 94%). ^1H NMR (400 MHz, DMSO-d_6) δ (ppm) 13.57 (br. s, 2H, COOH.), 9.21 (s, 3H, pymr-H), 8.45 (t, *J* = 2.10 Hz, 1H, isoph-H), 8.28 (d, *J* = 2.10 Hz, 2H, isoph-H), 7.93 (d, *J* = 7.70 Hz, 2H, Ar-H), 7.79 (d, *J* = 7.70 Hz, 2H, Ar-H); ^{13}C NMR (101 MHz, DMSO-d_6) δ (ppm) 166.32, 158.04, 155.28, 136.06, 134.85, 132.95, 132.75, 132.63, 130.34, 127.72, 123.63, 122.68, 90.94, 89.25; MS (ESI): *m/z* = calc. 345.0870, found 345.0862 (M + H); ATR FT-IR: ν (cm^{-1}) 1721(s), 1278(m), 1200(m), 1177(w) 1160(w), 1111(m), 1007(m), 921(w), 834(m), 754(w), 710(w), 668(m), 653(m), 635(w), 562(w).

Preparation of 4''-(pyrimidin-5-yl)-[1,1':4',1''-terphenyl]-3,5-dicarboxylic acid (H_2L^6)

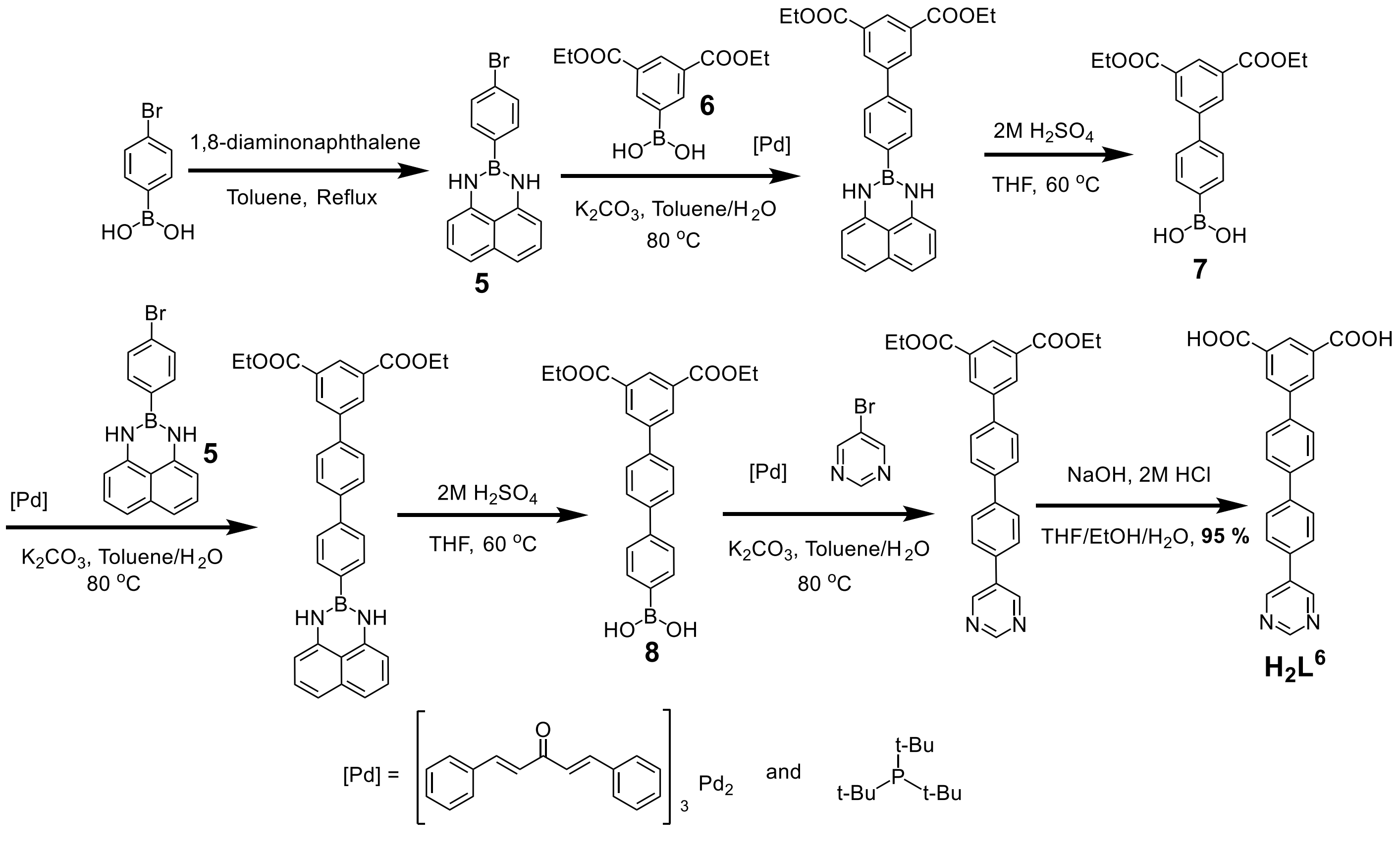

Scheme S7. Synthesis of H_2L^6.

To a degassed solution of **8** (2.30 g, 5.5 mmol), 5-bromopyrimidine (0.81 g, 5.0 mmol) and K_2CO_3 (2.30 g, 16.6 mmol) in toluene:water (280 mL, 4:1 v/v) at 60°C was added $P(^tBu)_3$ (2.0 mL, 1.0 mol solution in toluene, 2.0

mmol) and $Pd_2(dba)_3$ (0.7 g, 0.7 mmol). The reaction mixture heated to 80°C for 20 min and quenched with saturated aqueous NH_4Cl solution. The organic layer was extracted with CH_2Cl_2, washed with brine, and dried over $MgSO_4$. The solution was evaporated to dryness, the product washed with acetone and dried to yield the diethyl ester [diethyl 4'-(pyrimidin-5-yl)-[1,1'-biphenyl]-3,5-dicarboxylate (0.36 g, 67%)]. The product was hydrolysed using NaOH (0.40 g, 10 mmol) in EtOH:THF:water (120 mL, 1:1:1 v/v) by heating under reflux at 100°C overnight (ca. 17 h). The solution was reduced in volume to half *in vacuo* and the product precipitated by addition of 2M HCl to pH 1. The resulting solid was collected by filtration, washed with water and dried to yield H_2L^6 (0.73 g, 97%). ^{1}H NMR (400 MHz, DMSO-d_6) δ (ppm) 13.46 (br. S, 2 H, COOH.), 9.23 (s, 2 H, pymr-H), 9.21 (s, 1 H, pymr-H), 8.48 (t, J = 1.60 Hz, 2 H, Ar-H), 8.45 (d, J = 1.63 Hz, 2 H, Ar-H), 7.85 - 7.99 (m, 8 H, Ar-H); ^{13}C NMR (101 MHz, DMSO-d_6) δ (ppm) 166.99, 157.81, 155.14, 140.96, 140.22, 139.52, 138.23, 133.51, 133.16, 132.68, 131.64, 129.40, 128.06, 128.03, 127.99, 127.96; MS (ESI): *m/z* = calc. 397.1183, found 397.1191 (M+H); ATR FT-IR: ν (cm^{-1}) = 3119(w), 2474(w), 1867(m), 1601(m), 1579(m), 1566(s), 1504(w), 1454(w), 1415(s), 1345(m), 1305(s), 1251(w), 1116(m), 1073(s), 1037(s), 1004(m), 907(s), 742(s); Elemental analysis (Calc. in % for $C_{24}H_{16}N_2O_4\cdot0.5H_2O$): C, 71.10; H, 4.23; N, 6.91. Found: C, 71.18; H, 4.15; N 6.77.

Synthesis of MFM-126

H_2L^1 (10 mg, 0.04 mmol) and $Cu(NO_3)_2.3H_2O$ (20 mg, 0.08 mmol) were dissolved in DMF (4 mL) in a pressure tube and HCl (2M, 0.1 mL) was added to the mixture. The tube was tightly capped and heated in an oil bath at 80°C for 18 h to afford hexagonal green plates which were washed with DMF and filtered and dried to yield MFM-126 (6.0 mg, 34%). ATR FT-IR: ν(cm^{-1}) = 1693(s), 1564(s), 1413(s), 1332(m), 1255(s), 1087(w), 908(s), 758(m), 709(m), 656(m), 596(m). Analysis (Calc. in % for $C_{13}H_7N_3O_5Cu\cdot0.75DMF\cdot2.25H_2O$): C, 41.38; H, 3.47; N, 11.87. Found: C, 41.07; H, 3.83; N, 11.75.

Synthesis of MFM-127

H_2L^2 (10 mg, 0.04 mmol) and $Cu(NO_3)_2\cdot3H_2O$ (20 mg, 0.08 mmol) were dissolved in DMF (4 mL) and EtOH (1 mL) in a pressure tube and HCl (2M, 0.1 mL) was added to the mixture. The tube was tightly capped and heated in the oven at 80°C for 24 h to afford green crystals which were washed with DMF (5 mL x 3 mL), filtered off, rinsed with acetone and dried to give MFM-127 (8 mg, 52%). ATR FT-IR: ν (cm^{-1}) 1667 (s), 1633 (s), 1580.83(m), 1553(w) 1431 (w), 1373 (m), 1268(s), 1187 (w), 1181 (m) 1098 (w), 1091 (w) 767 (w), 730 (m), 711 (m), 659 (w), 645 (w), 569 (w), 557 (w); Elemental Analysis [(%), (Calc. for $C_{14}H_6N_2O_4Cu\cdot3.09H_2O$)]: C, 43.63; H, 3.18; N, 7.27. Found: C, 43.63; H, 2.86; N, 7.54.

Synthesis of MFM-128

H_2L^3 (10 mg, 0.03 mmol), $Cu(NO_3)_2.3H_2O$ (22 mg, 0.09 mmol) and 2M HCl (0.1 mL) was dissolved in DMA/DMSO (5 mL, 1:1 v/v) in Ace pressure tube (bushing type, front sealed) and heated in oil bath at 80°C for 48 h. The resulting green hexagonal plates were thoroughly rinsed with DMF and then acetone and filtered and dried to give MFM-128 (7 mg, 52%); ATR FT-IR: ν (cm^{-1}) = 1632 (s), 1589 (s), 1448 (w), 1414 (w), 1370 (m), 1297 (s), 1254 (m), 1088 (w), 1009 (w), 836 (w), 775 (m), 760 (w), 728 (m), 717 (w), 655 (w), 646 (w), 635 (m), 560 (w); Elemental Analysis (Calc. in % for $C_{24}H_{14}N_2O_4Cu{\cdot}2H_2O$): C, 51.74; H, 3.38; N, 6.70. Found: C, 51.54; H, 2.75; N, 6.59.

Synthesis of MFM-136

This was completed from the published procedure.[1]

Synthesis of MFM-137

H_2L^5 (20 mg, 0.06 mmol) and $Cu(NO_3)_2{\cdot}3H_2O$ (44 mg, 0.12 mmol) were added to a vial containing a mixture of DMF (4 mL) and DMSO (1 mL) containing 2M HCl (0.1 mL) and heated in an oven at 80°C for 18 h. The resulting green hexagonal plates were thoroughly rinsed sequentially with DMF and acetone, then filtered off and dried to give MFM-137 (16 mg, 68%). ATR FT-IR: ν (cm^{-1}) 1667 (s), 1632 (m), 1586 (m), 1417 (w) 1372 (s), 1089 (m), 921 (w), 834 (m), 773 (w), 730 (w), 718 (m), 658 (w), 557 (m). Elemental Analysis (Calc in % for $C_{18}H_{10}N_2O_4Cu{\cdot}2H_2O$): C, 55.25; H, 4.03; N, 5.60. Found: C, 55.59; H, 4.07; N, 5.19.

Synthesis of MFM-138

H_2L^6 (10 mg, 0.025 mmol), $Cu(NO_3)_2.3H_2O$ (12 mg, 0.05 mmol) and 2M HCl (0.1 mL) was dissolved in N,N'-diethylformamide (4 mL) in an Ace pressure tube (bushing type, front sealed) and heated in oil bath at 80°C for 48 h. The resulting green hexagonal plates were thoroughly rinsed with DMF and then acetone and filtered and dried to give MFM-138 (7 mg, 45%). ATR FT-IR: ν (cm^{-1}) = 1668 (s), 1633 (m), 1587 (w), 1447 (w), 1407 (m), 1370 (s), 1297 (w), 1089 (w,) 821 (m), 773 (s), 747 (w), 718 (s), 656 (w), 6360 (w). Elemental Analysis (Calc in % for $C_{18}H_{10}N_2O_4Cu{\cdot}2H_2O{\cdot}C_3H_6O$): C, 62.93; H, 3.08; N, 6.12. Found: C, 63.49; H, 3.62; N, 5.92.

ATR-FTIR spectra of MFM-126 – MFM-138 and their respective linkers

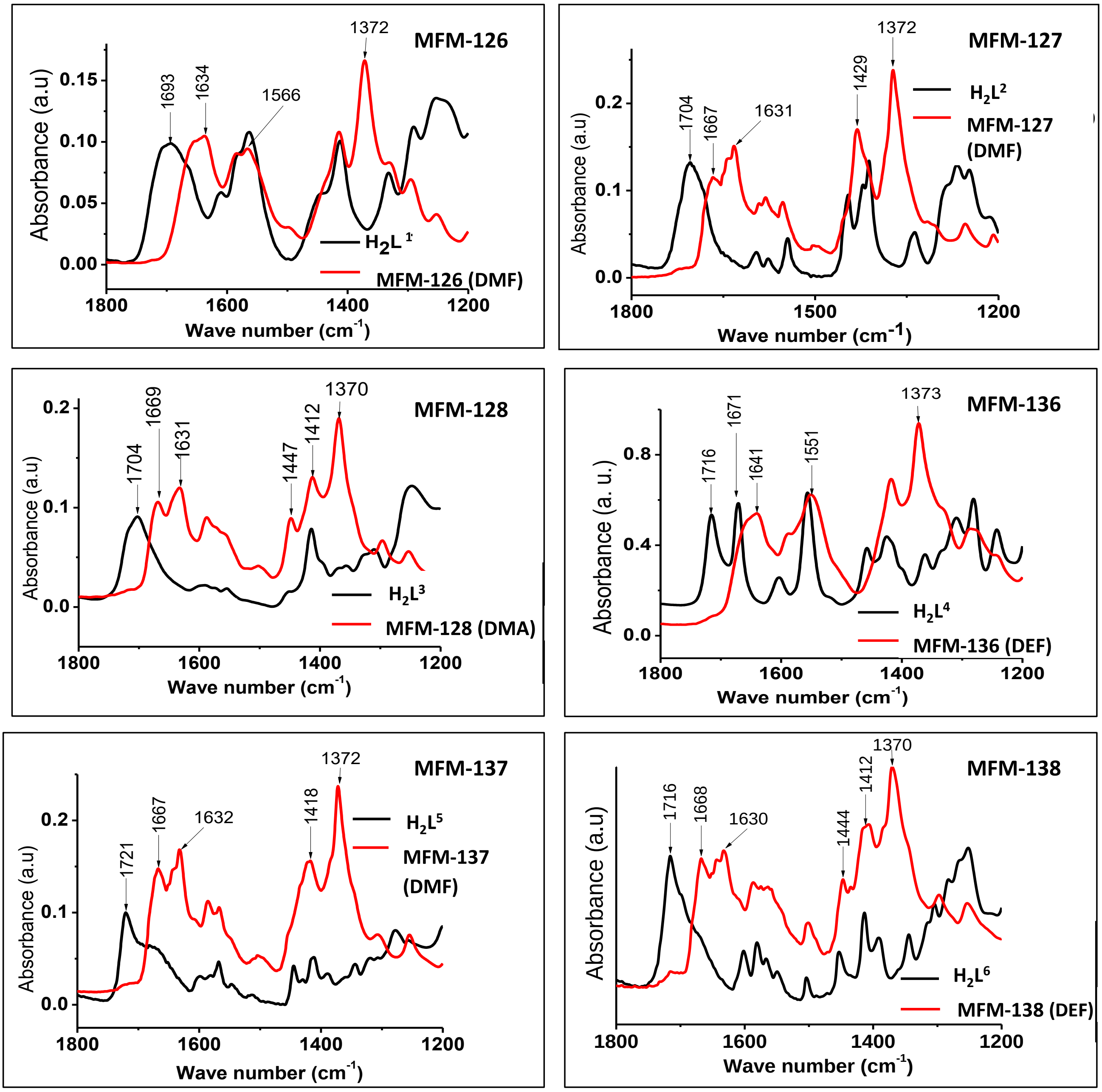

Figure S1. ATR-FTIR spectra of MFM-126-128 and MFM-136-138 and of the corresponding linkers H_2L^{1-6}. Labelled peaks highlight the shift of carbonyl stretching vibrations from 1693 cm^{-1} to 1634 cm^{-1}; 1704 cm^{-1} to 1667 cm^{-1}; 1704 cm^{-1} to 1669 cm^{-1}; 1716 cm^{-1} to 1641 cm^{-1}; 1721 cm^{-1} to 1667 cm^{-1} and 1716 cm^{-1} to 1668 cm^{-1}, from free linkers H_2L^{1-6} to corresponding MOFs, respectively. The depletion of these carbonyl bands in the spectra of the MOFs indicates the absence of residual unbound linkers in the respective MOF materials.

Thermogravimetric Analyses (TGA) of MFM-126-128 and MFM-136-138

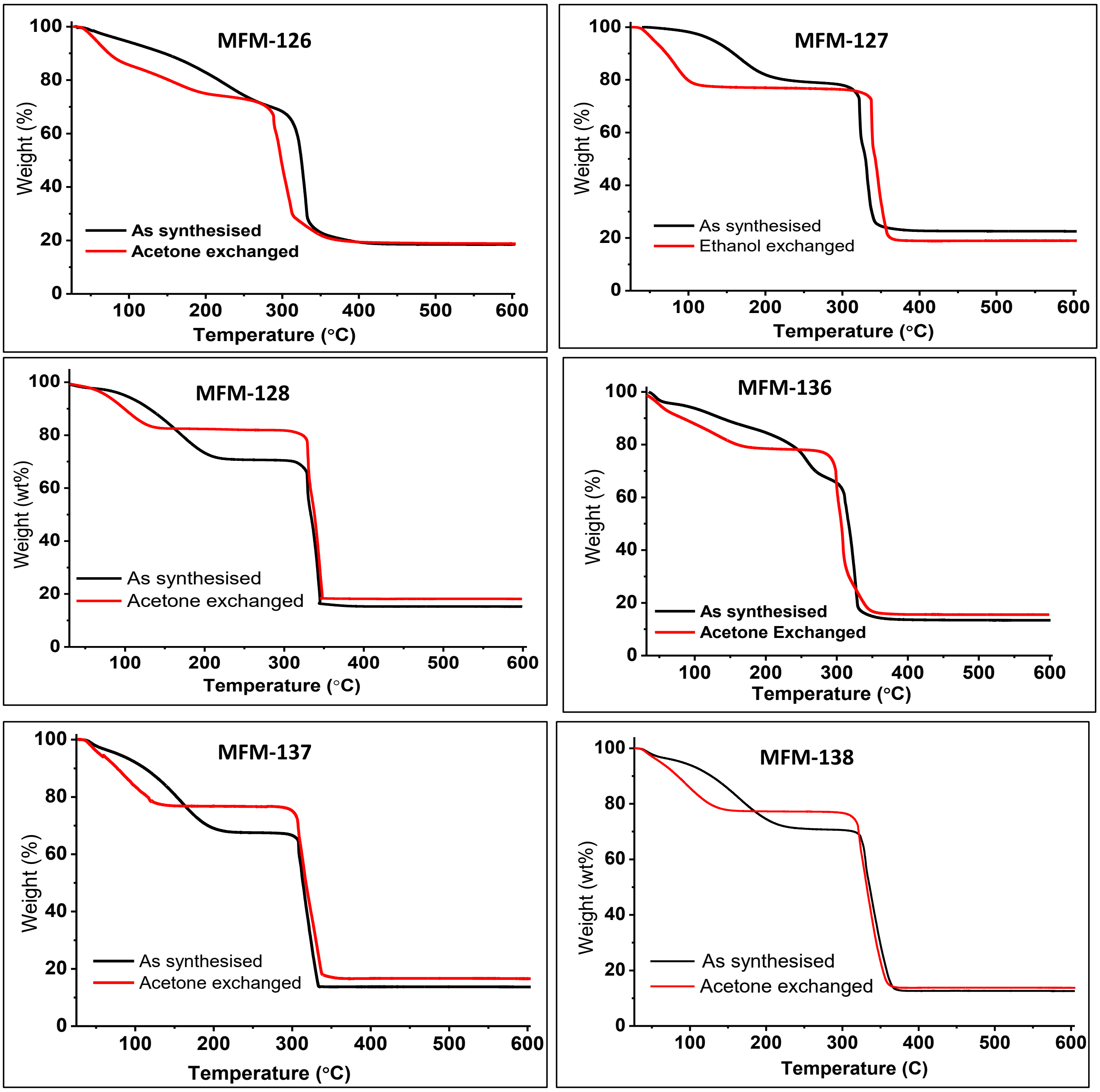

Figure S2. TGA thermographs of as-synthesised and solvent-exchanged samples of MFM-126-128 and MFM-136–138. The measurements were carried out under a flow of air at a rate of 100 mL min^{-1}.

2. X-Ray Crystallography

Single Crystal X-ray Diffraction Structure Determinations

MFM-126, MFM-137 and MFM-138 (Rigaku Supernova); MFM-128 and MFM-127 (I19 beamline, Diamond Light Source)

Diffraction data were collected on a Rigaku Oxford Diffraction SuperNova diffractometers equipped with Atlas detectors and microfocus Mo or Cu X-ray sources. X-ray data for MFM-127 were collected using a synchrotron radiation at single crystal X-ray diffraction beamline I19 in Diamond light Source,[2] equipped with a Pilatus 2M detector and an Oxford Cryosystems nitrogen flow gas system. MFM-127 data were measured using GDA suite of programs. The raw data were reduced and corrected for Lorentz and polarisation effects using CrysAlisPro;[3] corrections for the effects of adsorption were applied using a numerical absorption correction based on Gaussian integration over a multifaceted crystal model. All structures were solved by direct methods (SHELXS)[4] and refined by full-matrix least-squares (SHELXL).[5] Regions of diffuse solvent in the solvated structures were treated with the PLATON SQUEEZE routine.[6]

MFM-126

The diffraction of the crystal was weak with little intensity beyond 0.9 Å resolution. This is likely a consequence of large volumes of poorly ordered solvent (total void fraction 0.44) and conformational disorder in the ligand. Conformational disorder was observed in pyrimidine ring N11-C16, amide moiety C17-N19 and phenyl ring C21-C26. The occupancies of the two pyrimidine ring components were refined before each being fixed at a value of 0.5. The occupancies of the two phenyl ring components were freely refined and constrained to sum to unity (occupancy of component A 0.35(1)). The four conformations of the amide moiety were constrained to have a value of half of the occupancy of the phenyl ring with which they share connectivity. The phenyl ring was constrained to have regular hexagonal geometry (AFIX 66), and the pyrimidine ring was constrained to have planar geometry (FLAT). The 1,2 and 1,3 distances around the disordered pair of pyrimidine rings were restrained to reflect the C_{2v} symmetry of the pyrimidyl moiety (SADI). Pairs of amide nitrogen atoms N19C/N19D and N19E/N19F were constrained to occupy the same sites and have identical isotropic thermal displacement parameters (EXZY/EADP). The three atoms of each amide moiety and connected pyrimidine carbon atom were fixed to have co-planar geometry (FLAT). All atoms except those of the disordered amide moieties have been refined with anisotropic displacement parameters. Rigid bond and similarity restraints have been applied to the displacement parameters of all the atoms in the structure (RIGU, SIMU). Hydrogen atoms were placed geometrically and refined using a riding model. Disordered solvent molecules could not be sensibly modelled, so the structure was treated with PLATON SQUEEZE.[6] A total of 1154 electrons were accounted from the *P*1 cell, equating to 1.5 dimethylformamide molecules per asymmetric unit, which have been included in the unit cell contents and calculation of derived parameters. A large positive electron density peak (3.94 e Å^{-3}) lies on the three-fold symmetry axis 2.00 Å from disordered pyrimidine nitrogen atoms N13A and N13B. The electron density is too close to the pyrimidine moieties to be plausibly modelled as a solvent.

Table S1. Summary of single crystal X-ray diffraction data for MFM-126

	**MFM-126**
**Formula**	$C_{17.5}H_{17.5}CuN_{4.5}O_{6.5}$
**Formula weight**	458.40
**Temperature (K)**	120(2)
**Crystal system**	trigonal
**Space group**	*R*-3
a (Å)	18.5219(8)
b (Å)	18.5219(8)
c (Å)	34.897(3)
**α (°)**	90
**β (°)**	90
**γ (°)**	120
V **(Å^3)**	10367.8(13)
Z	18
**Density** (g cm^{-3})	1.322
**μ** (mm^{-1})	1.703
***F*(000)**	4230
**Crystal size (mm^3)**	0.11 × 0.06 × 0.05
**Radiation (Å)**	Cu *K*α (λ = 1.54184)
**2Θ range for data collection (°)**	6.064 to 148.624
**Reflections collected**	13039
**Independent reflections**	3410 (R_{int} = 0.0452)
**Data/restraints/parameters**	4527/279/272
**Goodness-of-fit on F^2**	1.287
**Final *R* indexes [*I* ≥ 2σ(*I*)]**	R_1 = 0.1213, wR_2 = 0.3815
**Final *R* indexes [all data]**	R_1 = 0.1213, wR_2 = 0.3815
**CCDC Deposit Number**	1857732

Table S2. Selected bond lengths and valence angles for MFM-126

**MFM-126**						
**Bond Length/Å**			**Bond Angle/°**			
Cu1	Cu1	2.6334(19)	O32	Cu1	O33	167.8(2)
Cu1	O32	1.961(5)	O32	Cu1	O42	89.2(3)
Cu1	O33	1.954(5)	O33	Cu1	O42	89.2(2)
Cu1	O42	1.964(5)	O43	Cu1	O32	89.1(3)
Cu1	O43	1.951(5)	O43	Cu1	O33	90.0(3)
Cu1	N11	2.185(11)	O43	Cu1	O42	168.0(2)
C41	O42	1.277(3)	O32	C31	O33	125.7(7)
C41	O43	1.249(4)	O42	C41	O43	125.6(7)
C51	O52	1.249(3)				
C51	O53	1.263(3)				

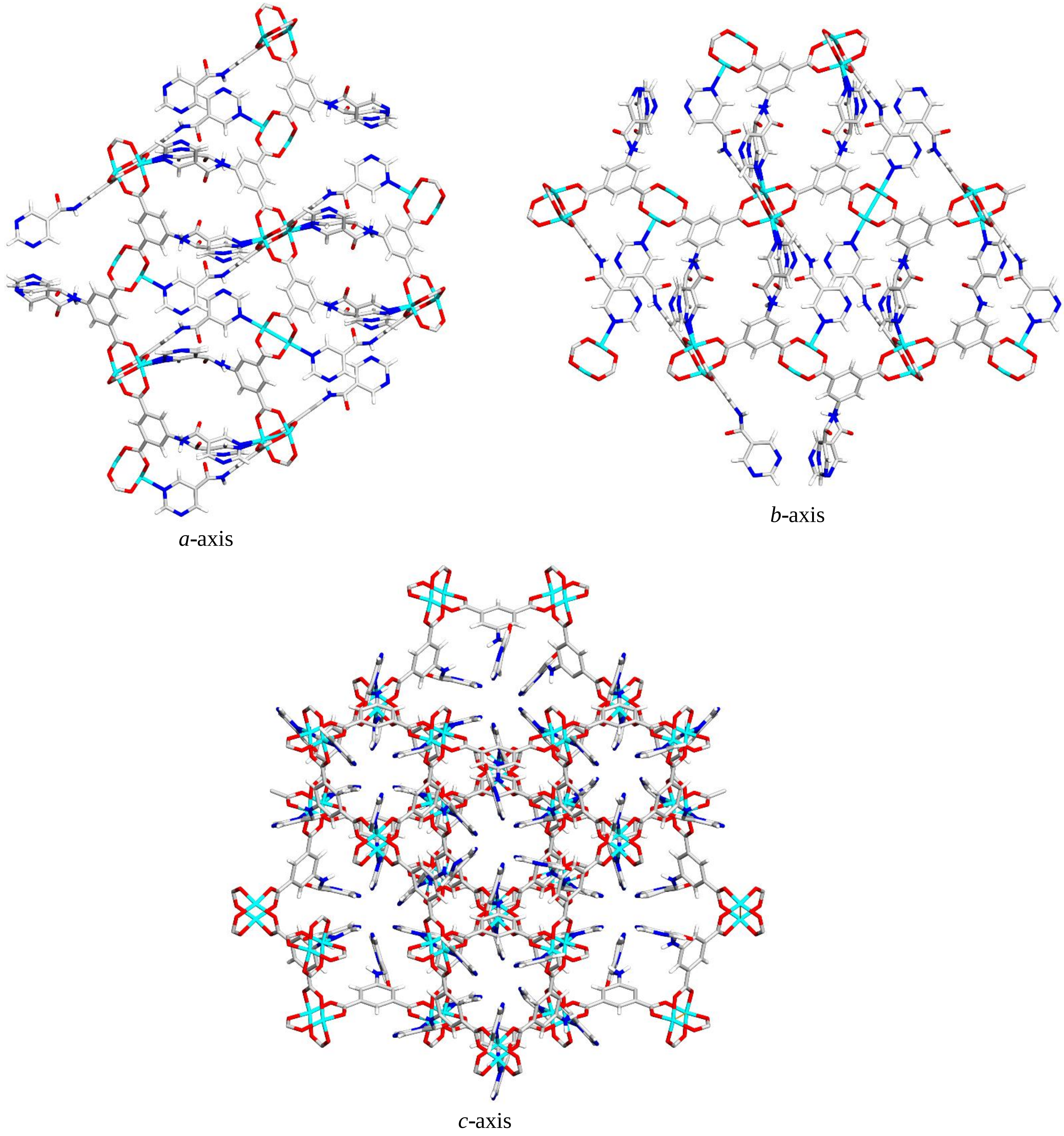

Figure S3. Views of the crystal structure of MFM-126 along the *a*-, *b*- and *c*-axes.

MFM-127

A residual electron density peak with a height of 1.65 e A^{-3} is located 0.662 A from copper atom Cu1. The electron density peak possibly arises as a result of an unmodeled disorder component of copper atom Cu1 which in turn could be caused by an alternative conformation of the pyrimidine ring. The alternative pyrimidine ring conformation is observed as a disorder component when the structure is solved the lower symmetry space group R-3. An attempt to model the peak as a disorder site with copper atom Cu1 resulted in a refined fractional occupancy of 0.04 (the occupancies of the two sites were constrained to sum to unity). No sensible model for associated ligand disorder could be modelled and the structure is reported with a single full occupancy copper site. Disordered solvent molecules could not be sensibly modelled, so the structure was treated with PLATON SQUEEZE.[6] A total of 1747 electrons were accounted from the P1 cell in this, equating to 1.2 dimethylformamide molecules per asymmetric unit, which have been included in the unit cell contents and calculation of derived parameters.

Table S3. Summary of single crystal X-ray diffraction data for MFM-127

	**MFM-127**
**Formula**	$C_{16.4}H_{14.4}N_{3.2}O_{5.2}Cu$
**Formula weight**	403.05
**Temperature (K)**	120.0(2)
**Crystal system**	trigonal
**Space group**	*R*-3c
a (Å)	17.9877(1)
b (Å)	17.9877(1)
c (Å)	72.1623(4)
**α (°)**	90
**β (°)**	90
**γ (°)**	120
V **(Å^3)**	20220.5(3)
Z	36
**Density** (g cm^{-3})	1.192
**μ** (mm^{-1})	0.921
F**(000)**	7409
**Crystal size (mm^3)**	0.1 × 0.1 × 0.1
**Radiation** (Å)	0.6889; Synchrotron (I19-1, DLS)
**2Θ range for data collection (°)**	2.754 to 68.4
**Reflections collected**	140455
**Independent reflections**	11355 (R_{int} = 0.0587)
**Data/restraints/parameters**	8114/75/190
**Goodness-of-fit on F^2**	0.999
**Final *R* indexes [*I* ≥ 2σ(*I*)]**	R_1 = 0.0364, wR_2 = 0.1070
**CCDC Deposit Number**	1857733

Table S4. Selected bond lengths and valence angles for MFM-127

MFM-127						
**Bond Length/Å**			**Bond Angle/°**			
Cu1	Cu1	2.6495(9)	O32	Cu1	O42	89.62(12)
Cu1	O32	1.942(2)	O32	Cu1	O33	167.81(9)
Cu1	O42	1.952(2)	O32	Cu1	O43	89.25(12)
Cu1	O33	1.968(3)	O42	Cu1	O33	89.04(12)
Cu1	O43	1.967(2)	O42	Cu1	O43	167.79(9)
Cu1	N11	2.138(12)	O43	Cu1	O33	89.50(12)
C31	O32	1.262(4)	O33	C31	O32	126.7(3)
C31	O33	1.241(4)	O43	C41	O42	126.2(4)
C41	O42	1.260(4)				
C41	O43	1.245(4)				

MFM-128

Phenyl ring C21-C26 was found to be disordered over two orientations. The occupancies of the two components were refined and constrained to sum to unity with values 0.63(1) and 0.37(1). The two disordered ring components were restrained to each have planar geometry (FLAT) and the chemically equivalent 1,2- and 1,3-distances around the ring were restrained to be similar (SADI). Rigid bond restraints (RIGU) were applied to the anisotropic thermal displacement parameters of all atoms in the structure. Similarity restraints were applied the anisotropic thermal displacement parameters of the disordered phenyl rings (SIMU). Unmodeled residual electron density peaks remain in the fourier map close to uncoordinated pyrimidine nitrogen atom N13 (Q1 1.39 e Å $^{-3}$, 1.088 Å; Q2 1.29 e Å $^{-3}$, 2.134 Å). The positions of the peaks indicate they might represent a carboxylate group from a minor ligand disorder component. No sensible model for such disorder could be developed, and the electron density is too close to the main residue to be treated with PLATON SQUEEZE.[6] A damping factor was used in the refinement to aid problematic convergence of the esd on the z coordinate of Cu1. Disordered solvent molecules could not be sensibly modelled, so the structure was treated with PLATON SQUEEZE.[6] A total of 3030 electrons were accounted from the *P*1 cell in this, equating to 2 dimethylacetamide molecules per asymmetric unit, which have been included in the unit cell contents and calculation of derived parameters.

Table S5. Summary of single crystal X-ray diffraction data for MFM-128

	**MFM-128**
**Formula**	$C_{26}H_{28}CuN_4O_6$
**Formula weight**	556.06
**Temperature (K)**	120(2)
**Crystal system**	trigonal
**Space group**	*R*-3*c*
a (Å)	18.3805(4)
b (Å)	18.3805(4)
c (Å)	83.574(2)
**α (°)**	90
**β (°)**	90
**γ (°)**	120
V **(Å^3)**	24452.1(12)
Z	36
**Density** (g cm^{-3})	1.359
**μ** (mm^{-1})	1.523
***F*(000)**	10404
**Crystal size (mm^3)**	0.2736 × 0.1384 × 0.0846
**Radiation** (Å)	Cu *K*α (λ = 1.54184)
**2Θ range for data collection (°)**	6.982 to 149.102
**Reflections collected**	22725
**Independent reflections**	5491(R_{int} = 0.0311)
**Data/restraints/parameters**	5491/114/251
**Goodness-of-fit on F^2**	1.048
**Final *R* indexes [$I \geq 2\sigma(I)$]**	R_1 = 0.652, wR_2 = 0.1876
**Final *R* indexes [all data]**	R_1 = 0.0703, wR_2 = 0.1824
**CCDC Deposit Number**	1857734

Table S6. Selected bond lengths and valence angles for MFM-128

**MFM-128**						
**Bond Length/Å**			**Bond Angle/°**			
Cu1	Cu1	2.6513(8)	O42	Cu1	O43	91.08(11)
Cu1	O42	1.937(2)	O42	Cu1	O52	88.90(11)
Cu1	O43	1.986(2)	O42	Cu1	O53	170.88(9)
Cu1	O52	1.971(2)	O43	Cu1	O52	165.25(9)
Cu1	O53	1.958(2)	O53	Cu1	O52	89.59(12)
Cu1	N11	2.160(3)	O53	Cu1	O43	88.10(12)
C41	O42	1.266(4)	O43	C41	O42	125.5(3)
C41	O43	1.259(4)	O53	C51	O52	125.8(3)
C51	O52	1.267(4)				
C51	O53	1.254(4)				

MFM-137

Pyrimidyl ring N11-C16, phenyl ring C21-C26 and alkyne atom C27 were found to be disordered over two orientations. The occupancies of these disorder components were refined before being fixed at 0.5 each. Geometric similarity restraints were applied to the bond distances round the disordered phenyl and pyrimidyl rings (SADI). The disordered phenyl and pyrimidyl rings were restrained to have approximately planar geometries (FLAT). The disordered alkyne C-C triple bond distances were restrained to be the same length (SADI). Rigid bond and similarity restraints were applied to the anisotropic thermal displacement parameters of the disordered atoms (RIGU and SIMU). Several disordered solvent molecules could not be sensibly modelled, and so the structure was treated with PLATON SQUEEZE.[6] A total of 1811 electrons were accounted for in the P1 cell, equating to 2.5 dimethylformamide molecules per asymmetric unit, these are included in the chemical formula and in all quantities calculated from it.

Table S7. Summary of single crystal X-ray diffraction data for MFM-137

	**MFM-137**
**Formula**	$C_{27.5}H_{27.5}CuN_{4.5}O_{6.5}$
**Formula weight**	588.58
**Temperature (K)**	120.00(2)
**Crystal system**	trigonal
**Space group**	*R*-3
a (Å)	18.1817(4)
b (Å)	18.1817(4)
c (Å)	49.5775(17)
**α (°)**	90
**β (°)**	90
**γ (°)**	120
V **(Å^3)**	14193.4(7)
Z	18
**Density** (g cm^{-3})	1.239
**μ** (mm^{-1})	1.359
F**(000)**	5490.0
**Crystal size (mm^3)**	0.37 × 0.32 × 0.02
**Radiation** (Å)	Cu *K*α (λ = 1.54184)
**2Θ range for data collection (°)**	5.348 to 148.938
**Reflections collected**	22635
**Independent reflections**	6283 [R_{int} = 0.0514
**Data/restraints/parameters**	6283/295/355
**Goodness-of-fit on F^2**	1.041
**Final *R* indexes [$I \geq 2\sigma(I)$]**	R_1 = 0.0522, wR_2 = 0.1574
**Final *R* indexes [all data]**	R_1 = 0.0589, wR_2 = 0.1650
**CCDC Deposit Number**	1857735

Table S8. Selected bond lengths and valence angles for MFM-137

**MFM-137**						
**Bond Length/Å**			**Bond Angle/°**			
Cu1	Cu1	2.6521(5)	O42	Cu1	O43	167.82(6)
Cu1	O42	1.9522(16)	O42	Cu1	O53	89.62(8)
Cu1	O43	1.9646(17)	O43	Cu1	O53	88.83(9)
Cu1	O52	1.9523(16)	O52	Cu1	O42	89.36(8)
Cu1	O53	1.9655(17)	O52	Cu1	O43	89.65(8)
Cu1	N11	2.163(5)	O52	Cu1	O53	167.97(6)
C41	O42	1.263(3)	O43	C41	O42	125.4(2)
C41	O43	1.256(3)	O53	C51	O52	125.6(2)
C51	O52	1.263(3)				
C51	O53	1.259(3)				

MFM-138

Pyrimidyl ring N11-C16, phenyl rings C21-C26 and C31-C36 were found to be disordered over two orientations. The occupancies of these disorder components were refined before being fixed at 0.5 each, and Geometric similarity restraints were applied to the bond distances round the disordered phenyl rings (SADI). Rigid bond and similarity restraints were applied to the anisotropic thermal displacement parameters of the disordered atoms (RIGU and SIMU). Several disordered solvent molecules could not be sensibly modelled, and so the structure was treated with PLATON SQUEEZE.[6] A total of 1995 electrons were accounted for in the *P*1 cell, equating to 2 diethylformamide molecules per asymmetric unit, these are included in the chemical formula and in all quantities calculated from it.

Table S9. Summary of single crystal X-ray diffraction data for MFM-138

	**MFM-138**
**Formula**	$C_{34}H_{36}CuN_4O_6$
**Formula weight**	660.21
**Temperature (K)**	120(2)
**Crystal system**	trigonal
**Space group**	*R*-3
a (Å)	18.1118(3)
b (Å)	18.1118(3)
c (Å)	55.4016(10)
**α (°)**	90
**β (°)**	90
**γ (°)**	120
V **(Å^3)**	15738.9(6)
Z	18
**Density** (g cm^{-3})	1.254
**μ** (mm^{-1})	1.266
***F*(000)**	6210
**Crystal size (mm^3)**	0.094 × 0.08 × 0.05
**Radiation (Å)**	Cu *K*α (λ = 1.54184)
**2Θ range for data collection (°)**	4.786 to 119.988
**Reflections collected**	20947
**Independent reflections**	5217 [R_{int} = 0.0275
**Data/restraints/parameters**	5217/588/373
**Goodness-of-fit on *F*^{2}**	1.046
**Final *R* indexes [$I \geq 2\sigma(I)$]**	R_1 = 0.0511, wR_2 = 0.1367
**Final *R* indexes [all data]**	R_1 = 0.0584, wR_2 = 0.1430
**CCDC Deposit Number**	1857736

Table S10. Selected bond lengths and valence angles for MFM-138

**MFM-138**						
**Bond Length/Å**			**Bond Angle/°**			
Cu1	Cu1	2.6414(7)	O52	Cu1	O53	168.12(9)
Cu1	O52	1.952(2)	O52	Cu1	O62	89.00(11)
Cu1	O53	1.960(2)	O52	Cu1	O63	90.52(11)
Cu1	O62	1.951(2)	O53	Cu1	O62	90.51(11)
Cu1	O63	1.958(2)	O53	Cu1	O63	87.53(11)
Cu1	N11	2.15(3)	O62	Cu1	O63	168.17(9)
C51	O52	1.257(4)	O52	C51	O53	125.9(3)
C51	O53	1.258(4)	O62	C61	O63	125.7(3)
C61	O62	1.259(4)				
C61	O63	1.261(4)				

Powder X-ray Diffraction (PXRD) of MFM-126-128 and MFM-136-138

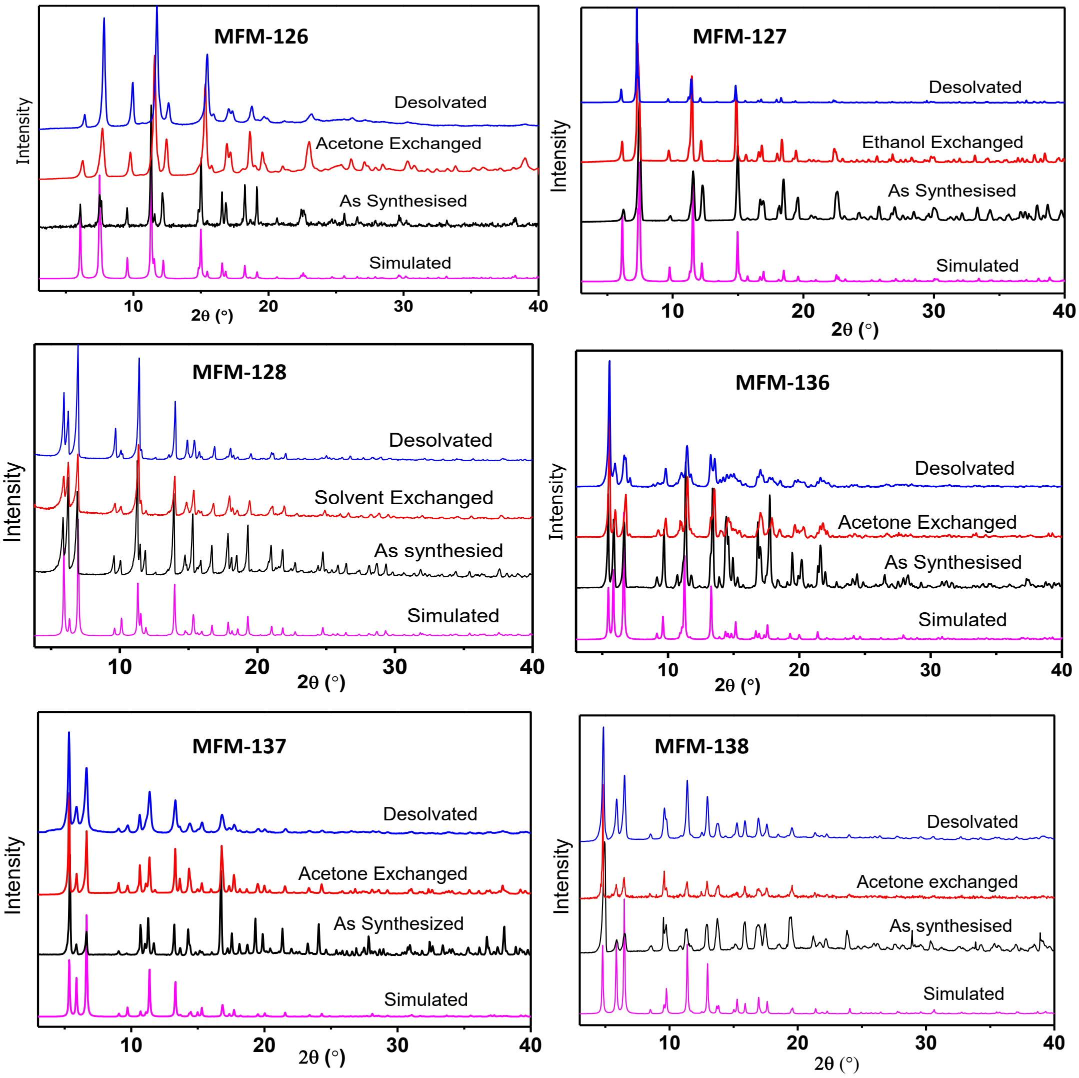

Figure S4. PXRD patterns for as-synthesised (black), solvent-exchanged (red), desolvated (blue) and simulated (pink) MFM-126-128 and MFM-136-138. Radiation Cu-K$_{\alpha 1}$ (λ = 1.5406 Å). The PXRD data confirm the phase purity of bulk MFM-126-138 samples and the retention of the framework structure upon removal of guest solvent molecules.

3. Gas Sorption

Gravimetric N_2, CO_2, CH_4 and C_2H_2 Adsorption

All MOFs were solvent-exchanged with acetone or EtOH before heating at 393 K under dynamic vacuum to produce the desolvated materials. Low pressure (0-1 bar) sorption isotherms for CO_2, CH_4 and N_2 and high pressure (0-20 bar) sorption isotherms for CO_2 and CH_4 were recorded using a Hiden Isochema Gravimetric Analyser (IGA-003) instrument. All isotherms were collected using ultrahigh vacuum diaphragm and turbo pumping systems, with ultra-pure research grade gases (99.9999%) purchased from BOC and used as received. Hiden temperature-controlled water baths were used to obtain isotherms at 273 and 298 K. The MOF samples were loaded from solvent into a sample basket in the sorption analyser and heated to 393 K under dynamic vacuum for 16 h to obtain fully activated samples (50-70 mg).

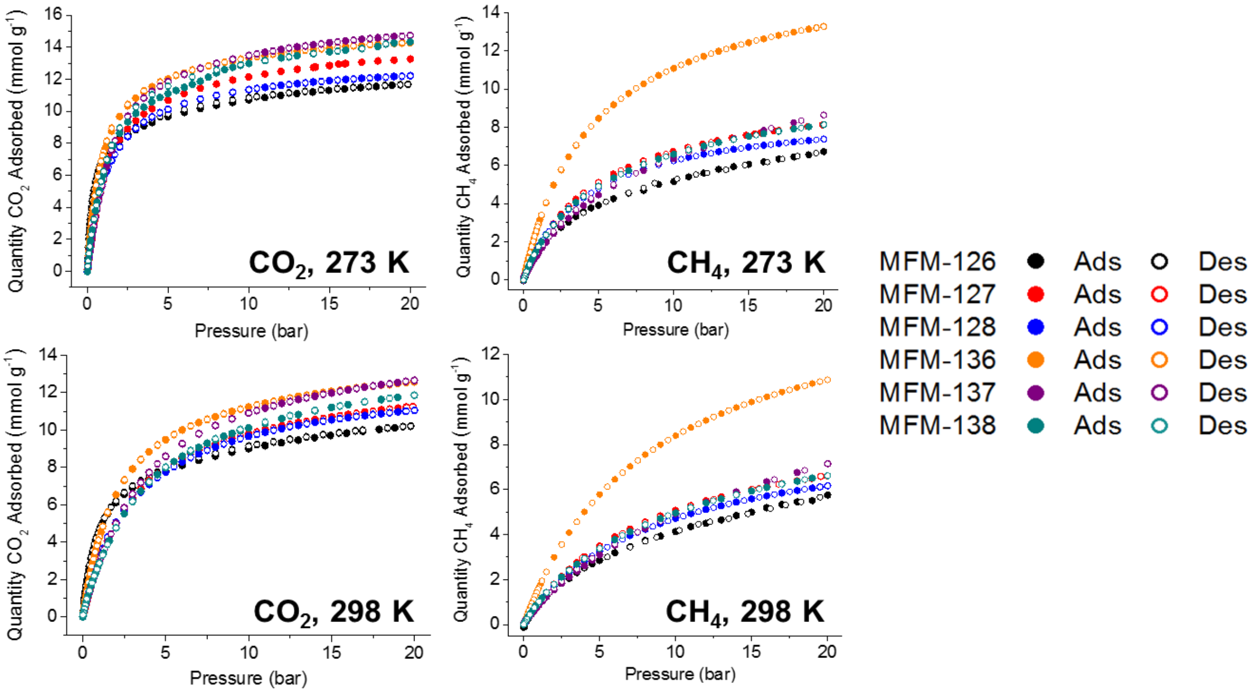

Figure S5. Gravimetric sorption isotherms (0-20 bar) for CO_2 and CH_4 in MFM-126-128 and MFM-136-138 at 273 K and 298 K.

Brunauer–Emmett–Teller (BET) Surface Areas

Volumetric N_2 adsorption data were recorded at 77 K (liquid nitrogen) on a Quantachrome Autosorb-1c instrument under ultra-high vacuum in a clean system with diaphragm and turbo pumping system using ultra-pure research grade (99.9999%) N_2. The BET surface areas were calculated using the software (version 1.60) integrated in the instrument. Pore size distribution data and cumulative pore volume were determined by analysis of the N_2 isotherms at 77 K using a non-local density functional theory (NLDFT) implementing a hybrid kernel. Vacuum dried powder samples were loaded on the instrument and degassed at 373 K and 10^{-9} bar for a minimum of 16 h to yield desolvated sample, which was then loaded in the instrument for N_2 adsorption measurements.

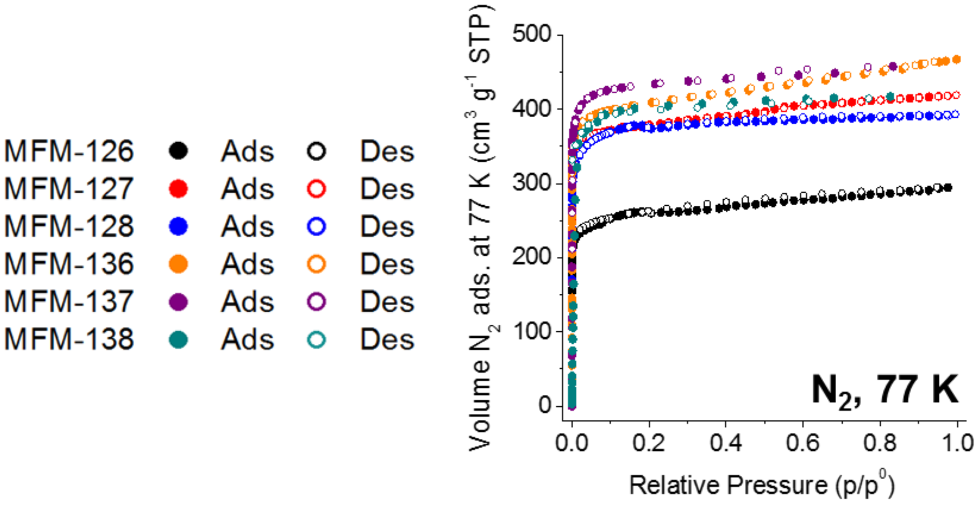

Figure S6. Volumetric N_2 isotherms for MFM-126-128 and MFM-136-138 at 77 K in the pressure range of 0-1 p/p^o.

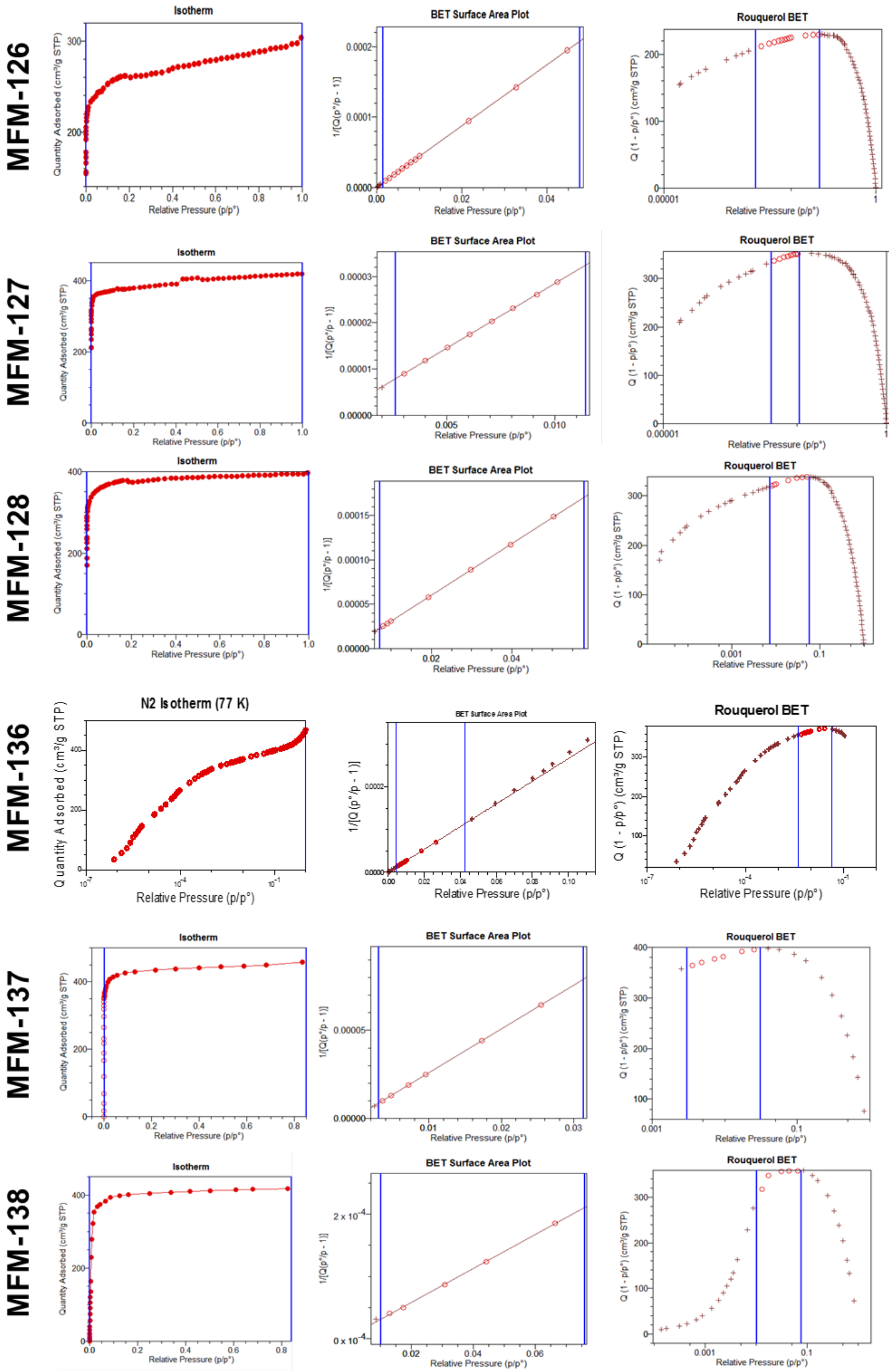

Figure S7. BET plots for series MFM-126-128 and MFM-136-138.

4. Neutron Powder Diffraction (NPD) Studies of MFM-126 and MFM-127

NPD data were collected at the WISH Diffractometer at ISIS Muon and Neutron Source, UK.[7] Acetone-exchanged MFM-126 and ethanol-exchanged MFM-127 were loaded into 11mm diameter vanadium sample cans and outgassed at 1 x 10^{-7} mBar and 130°C for 3 days. The samples were loaded into a liquid helium cryostat and cooled to 7 K for data collection of the bare framework. CO_2, C_2D_2 and CD_4 were volumetrically dosed from a calibrated volume, calibrated *via* use of the ideal gas equation ($PV = nRT$), after warming the sample to 293 K with data collected at various loadings of CO_2, C_2D_2 and CD_4 per copper of the materials. The sample can was isolated after reaching the target dosing amount to minimise the presence of "free gas" inside the can. The sample was then slowly cooled to 7 K (over ~ 3 h) to ensure adsorbates were completely adsorbed with no condensation elsewhere in the system. Sufficient time was allowed to achieve thermal equilibrium before data collection.

Rietveld Refinements of Guest Molecule Positions

The locations of CO_2 and CD_4 molecules within MFM-126 and MFM-127 as well as C_2D_2 in MFM-127 were determined as a function of gas loading by sequential Fourier difference map analysis followed by Rietveld refinement using the Topas software package.[8] Analysis of the Fourier map of the outgassed data indicated no residual nuclear density in the voids. The structure as solved by single crystal X-ray diffraction data was used as a starting point for the framework model which was geometrically restrained and refined against the NPD data. The framework atom coordinates were subsequently fixed before the models of guest molecules were developed. All binding sites were checked carefully for their unambiguous presence in the final structural model. Common C-O / C-D bond distances and isotropic thermal factors were included for the guest molecules. Final refinements comprised all free structural variables from both the framework and guest molecules.

NPD Refinements of CO_2 and CD_4 in MFM-126

Table S11. Summary of NPD Rietveld refinement statistics for gas-loaded MFM-126.

	**Bare MFM-126**	**1.2 CO_2**	**1.0 CD_4**
R_{exp}/%	0.25	0.25	0.24
R_{wp}/%	1.90	1.54	2.17
R_p/%	1.70	1.37	1.80
GoF	7.51	6.16	8.88
**CCDC Deposition number**	1857737	1857738	1857739

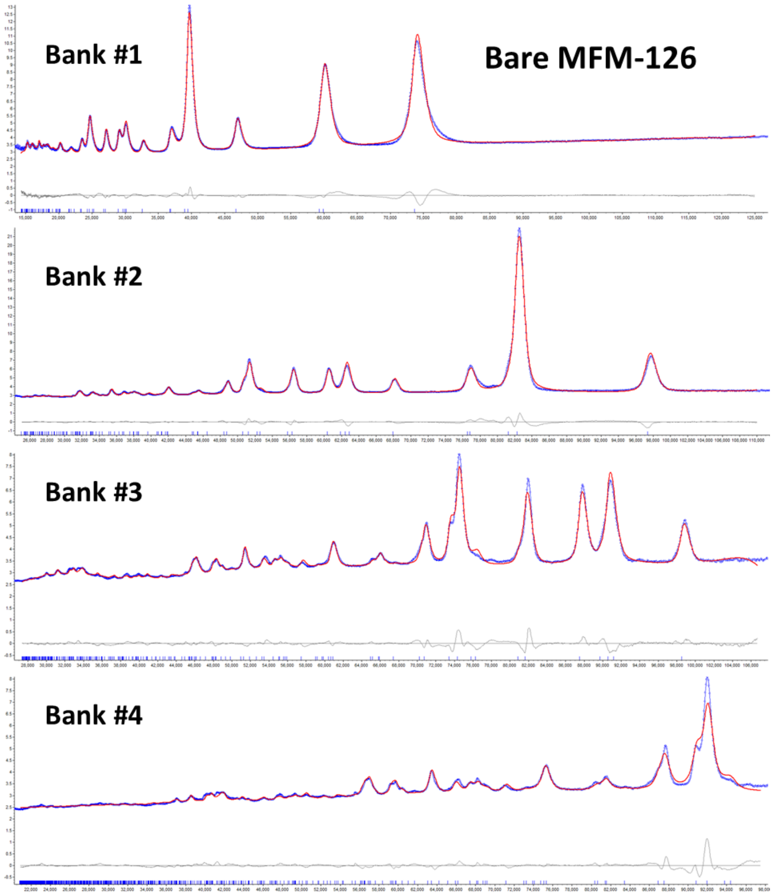

Figure S8. Observed (blue), calculated (red) and difference (grey) profiles of the Rietveld refinement of the neutron powder diffraction data (detector banks 1-4) for bare MFM-126

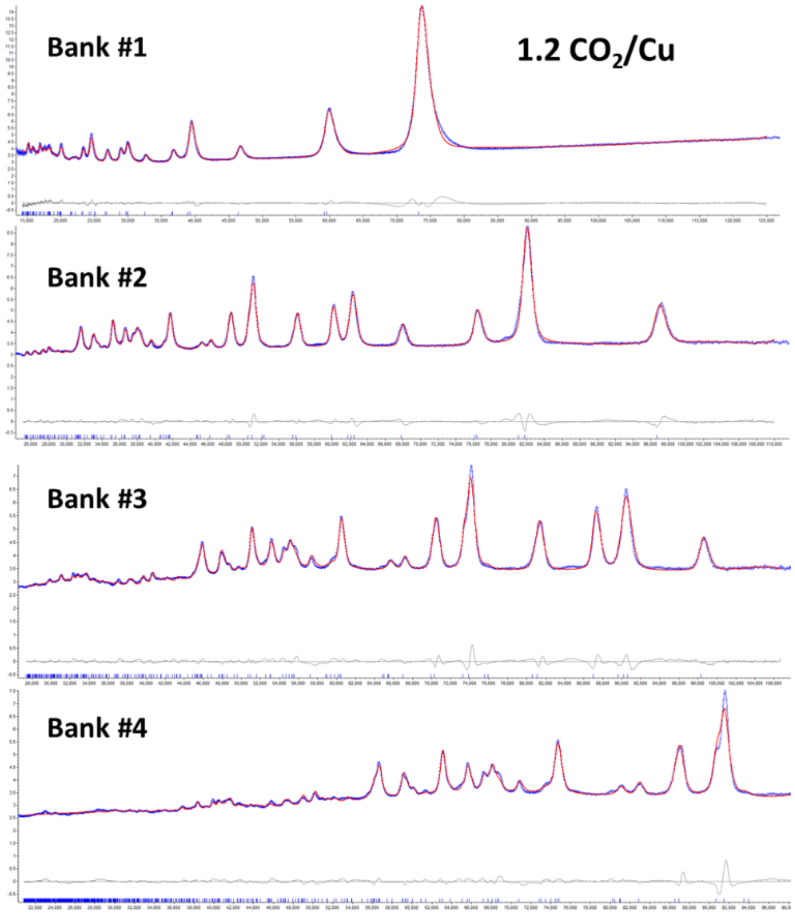

Figure S9. Observed (blue), calculated (red) and difference (grey) profiles of the Rietveld refinement of the neutron powder diffraction data (detector banks 1-4) for MFM-126 loaded with 1.0 CO_2 per Cu.

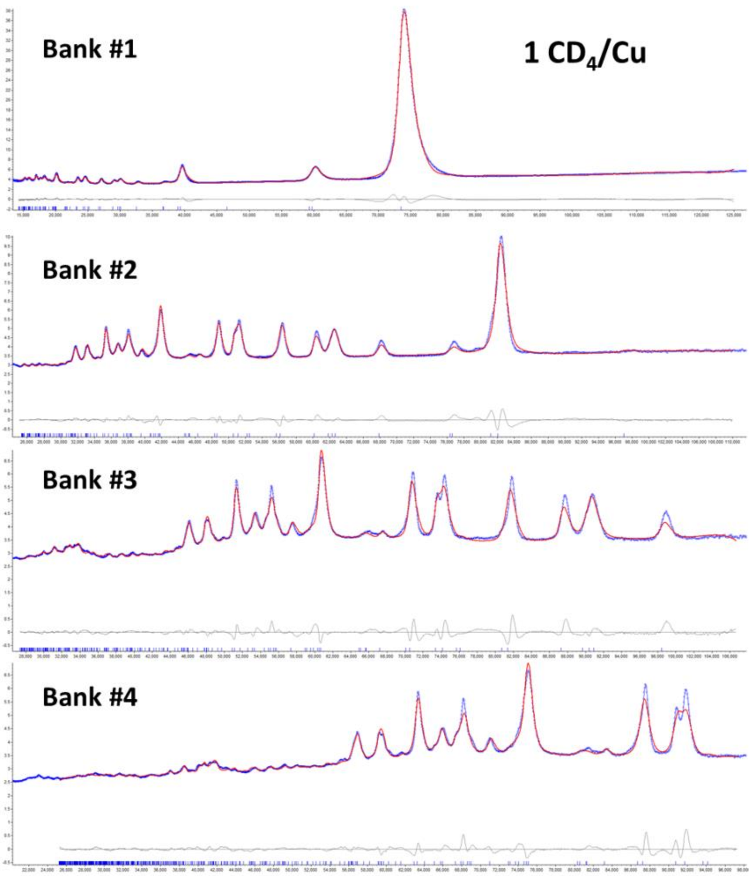

Figure S10. Observed (blue), calculated (red) and difference (grey) profiles of the Rietveld refinement of the neutron powder diffraction data (detector banks 1-4) for MFM-126 loaded with 1.0 CD_4 per Cu.

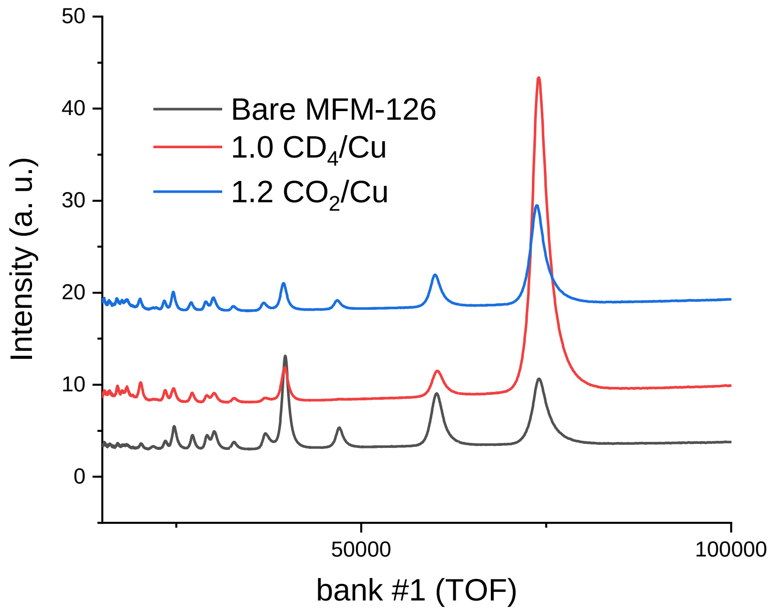

Figure S11. Comparison of the NPD patterns for bare and guest-loaded MFM-126

Table S12. Occupancies and short contacts for guest CO_2 sites determined from refinement of NPD data of MFM-126 loaded with 1.2 CO_2 per Cu(II) centre. Intramolecular distances quoted to highest level of precision supported by uncertainty.

**CO_2 Site**	**Multiplicity**	**Chemical Occupancy**	**Sum (occ. x multi.)**	**Clashes with other sites**	**Cage Location**	**Local Contact (Å)**	**Shortest Amide interaction (Å)**
**1**	36	0.415	14.940	n/a	B	$C–H_{pyrim}\cdots O_{CO_2}$ 2.32(5)	$O_{CO_2}\cdots N_{amide}$ 3.86(5)
**2**	18	0.333	6.000	n/a	B	$O_{CO_2}\cdots H–C_{isoph}$ 1.71(10)	n/a
**3**	36	0.328	11.808	4	WALL	$O_{CO_2}\cdots C_{pyrim}$ 2.44(5)	$O_{CO_2}\cdots H\text{-}N_{amide}$ 3.77(6)
**4**	36	0.284	10.224	3	WALL	$O_{CO_2}\cdots C_{isoph.}$ 3.14(11)	$O_{CO_2}\cdots H–N$ 4.14(9)

*WALL indicates guest sites located in the void space between cages **A** and **B.**

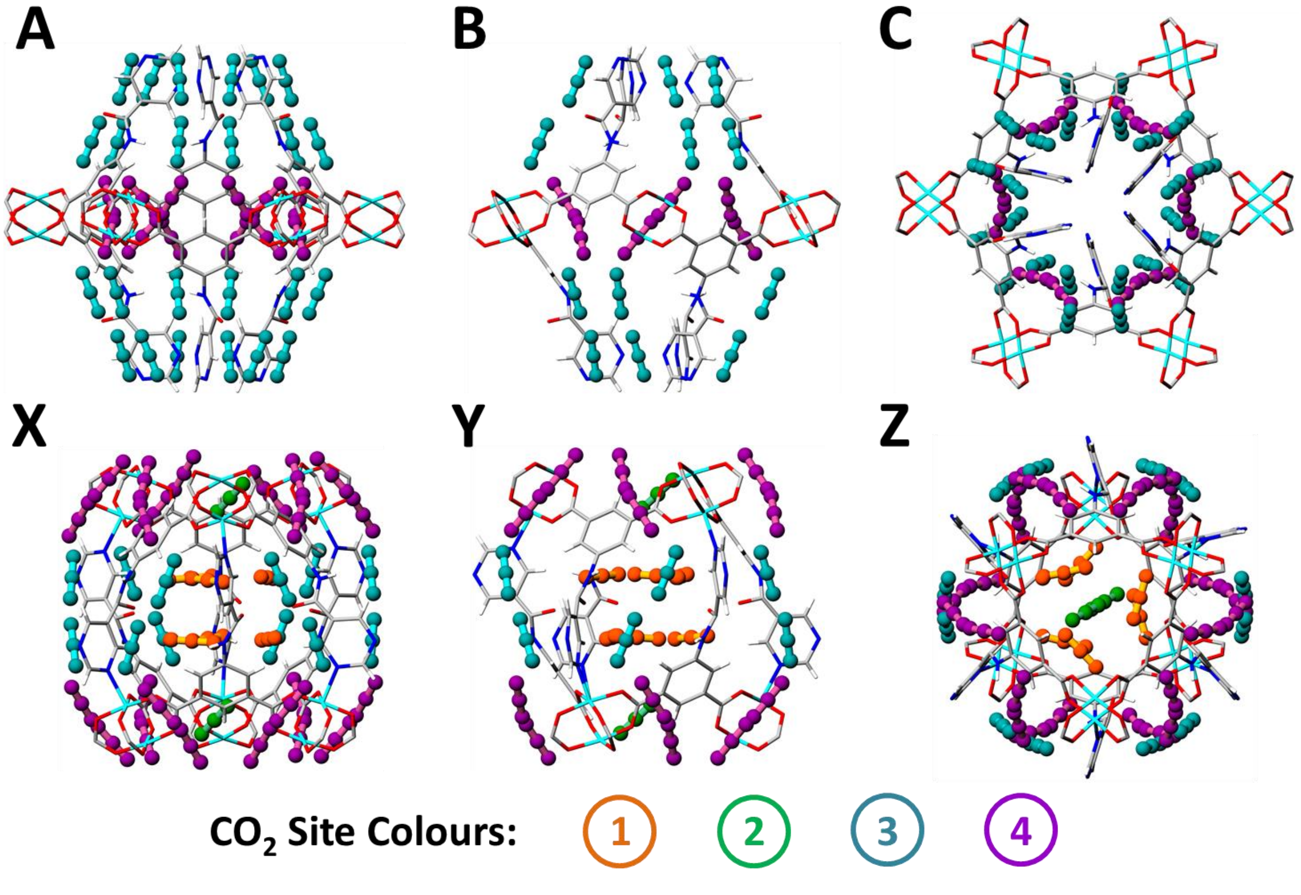

Figure S12. View of all the CO_2 binding sites in MFM-126 loaded at 1.2 CO_2 per Cu(II) centre by refinement of NPD data. (A), (B) and (C) are views of large cage **A** along the *a*, *b* and *c* axes, respectively. (X), (Y) and (Z) are views of small cage **B** along *a*, *b* and *c* axes, respectively. Colours: C, grey; H, white; O, red; N, blue; Cu, teal.

Table S13. Occupancies and short contacts for guest CD_4 sites determined from refinement of NPD data of MFM-126 loaded with 1.0 CD_4 per Cu(II) centre. Intramolecular distances quoted to highest level of precision supported by uncertainty.

**CD_4 Site**	**Multiplicity**	**Chemical Occupancy**	**Sum (occ. x multi.)**	**Clashes with other sites**	**Cage Location**	**Local Contacts (Å)**	**Shortest Amide Interaction (Å)**
**1**	12	0.630	7.56	n/a	WALL	D_4C···H–$C_{isophthal.}$ 2.96(11)	n/a
**2**	18	0.590	10.6	n/a	B	D_4C···H–$C_{isophthal.}$ 2.81(4)	D_4C···O=C_{amide} = 3.00(5)
**3**	6	0.380	2.28	n/a	A	D_4C-**3...**$C31_{isophthalate}$ 6.468(5)	n/a
**4**	18	0.250	4.50	5	WALL	D_4C-**4...**H32–$C32_{isoph}$ 4.361(17)	n/a
**5**	16	0.210	3.36	3,7	WALL	D_4C-**5..**$C14_{pyrimid}$ 4.619(8)	O28...CD_4-**5** 4.894(4)
**6**	18	0.180	3.24	n/a	A	D_4C-**6...**H14–$C14_{pyrim}$ 2.515(6)	N29…CD_4-**6** 4.242(5)
**7**	40	0.018	0.72	5	WALL	CD_4-**7**...O28 2.229(6)	O28...CD_4-**7** 2.229 (6)

*WALL indicates guest sites located in the void space between cages **A** and **B.**

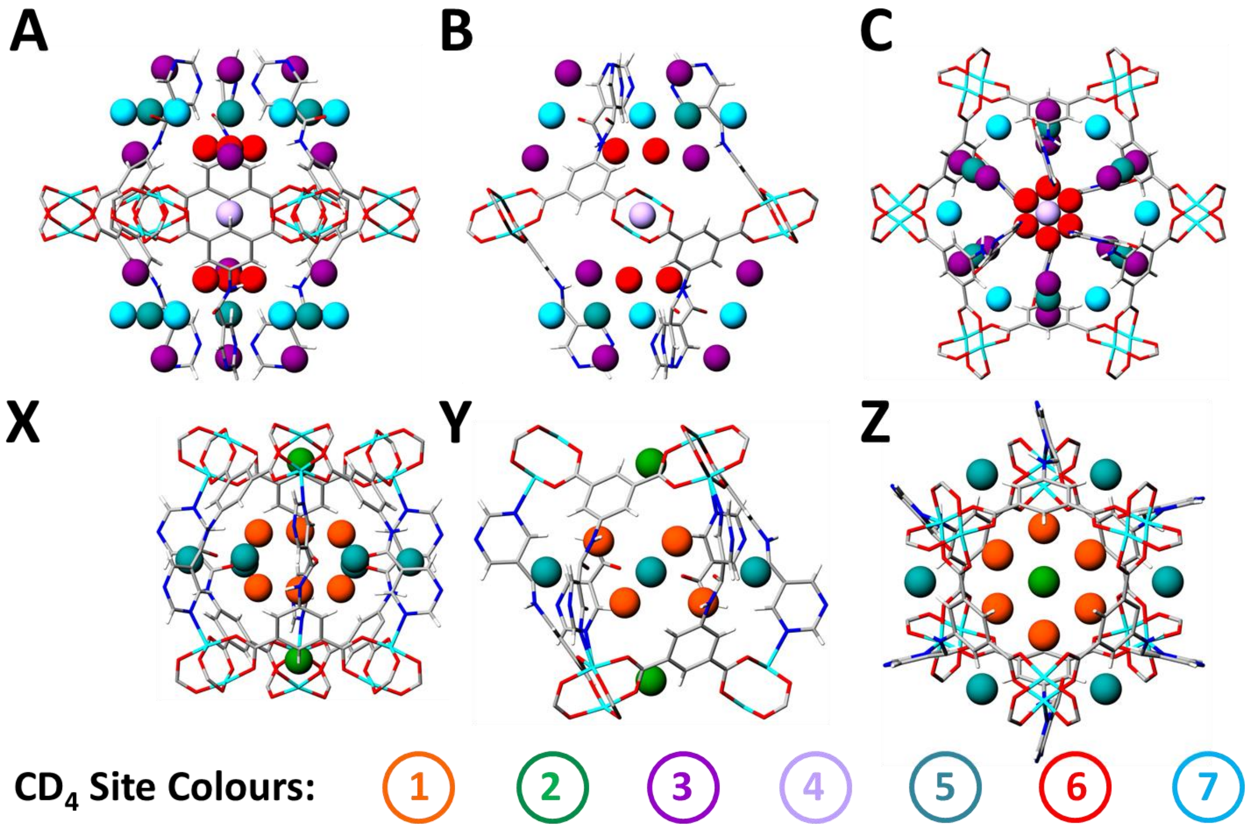

Figure S13. View of all the CD_4 binding sites in MFM-126 loaded at 1.0 CD_4 per Cu(II) centre by refinement of NPD data. (A), (B) and (C) are views of large cage **A** along the *a*, *b* and *c* axes, respectively. (X), (Y) and (Z) are views of small cage **B** along *a*, *b* and *c* axes, respectively. Colours: C, grey; H, white; O, red; N, blue; Cu, teal.

NPD Refinements of C_2D_2, CO_2 and CD_4 in MFM-127

Table S14. Summary of NPD Rietveld refinement statistics for gas-loaded MFM-127.

	**Bare MFM-127**	**1.2 CO_2**	**1.0 CD_4**	**1.0 C_2D_2**
$R_{exp/\%}$	0.238	0.229	0.232	0.233
$R_{wp/\%}$	1.25	1.22	1.47	1.23
$R_{p/\%}$	1.07	1.04	1.26	1.09
GoF	5.25	5.34	6.35	5.26
**CCDC Deposition Number**	1857740	1857741	1857742	1857743

Table S15. Occupancies, multiplicities, positions and short contacts for guest sites determined from refinement of NPD data of MFM-127 loaded with 1.0 C_2D_2 per Cu(II) centre. Intramolecular distances quoted to highest level of precision supported by uncertainty.

**C_2D_2 Site**	**Multiplicity**	**Chemical Occupancy**	**Sum (occ. x multi.)**	**Cage Location**	**Local Contacts (Å)**	**Shortest Alkyne interaction (Å)**
**1**	36	0.365	13.14	WALL	DC_2-D···$N_{pyrimidine}$ 2.54(5)	4.86(2)
**2**	36	0.247	8.892	WALL	**1**-D_2C≡C··· D-C_2D-**2** = 1.81(3)	3.89(5)
**3**	36	0.167	6.012	B	DC_2-D···η^2-C≡$C_{framework}$ = 2.68(5)	2.68(5)
**4**	36	0.116	4.176	B	DC_2-D···$O_{paddlewheel}$ = 2.25(5)	n/a
**5**	36	0.060	2.16	A	Pyrimid C-H...C_2D_2 **5** 2.20(7)	3.29(16)

*WALL indicates guest sites located in the void space between cages **A** and **B.**

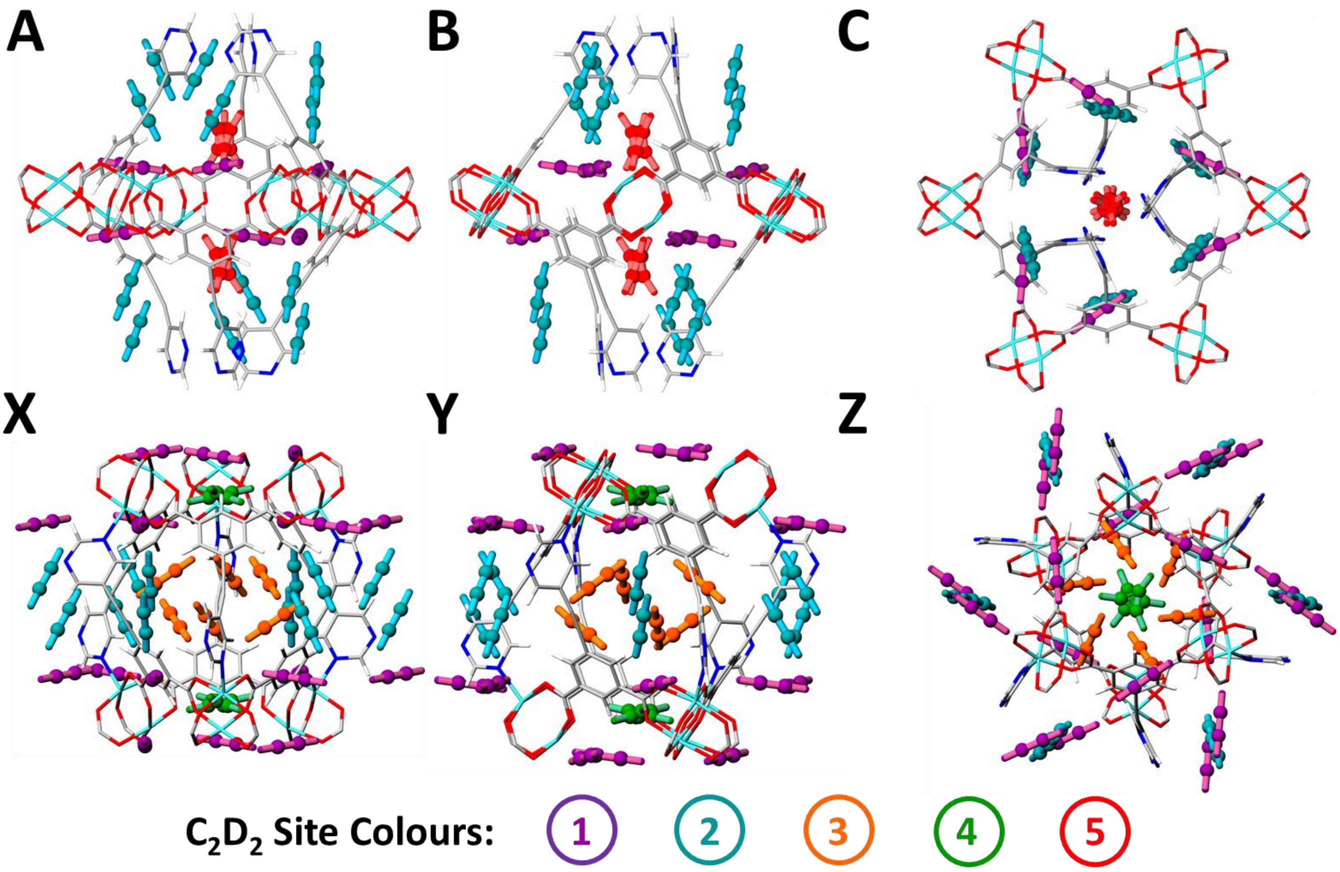

Figure S14. View of all the C_2D_2 binding sites in MFM-127 loaded at 1.0 C_2D_2 per Cu(II) centre by refinement of NPD data. (A), (B) and (C) are views of large cage **A** along the *a*, *b* and *c* axes, respectively. (X), (Y) and (Z) are views of small cage **B** along *a*, *b* and *c* axes, respectively. Colours: C, grey; H, white; O, red; N, blue; Cu, teal.

Table S16. Occupancies, multiplicities, positions and short contacts for guest CO_2 sites determined from refinement of NPD data of MFM-127 loaded with 1.0 CO_2 per Cu(II) centre. Intramolecular distances quoted to highest level of precision supported by uncertainty.

**CO_2 Site**	**Multiplicity**	**Chemical Occupancy**	**Sum (occ. x multi.)**	**Cage Location**	**Local Contacts (Å)**	**Shortest Alkyne Interaction (Å)**
**1**	24	1.00	24.000	B	CO_2 **1**...H-Cisoph 2.27(1)	n/a
**2**	36	0.445	16.020	B	CO_2 **2**...H-Cpyrim 2.87(4)	4.20(2)
**3**	36	0.103	3.708	WALL	CO_2 **3**...H-Cisoph 2.50(3)	5.79(6)

*WALL indicates guest sites located in the void space between cages **A** and **B.**

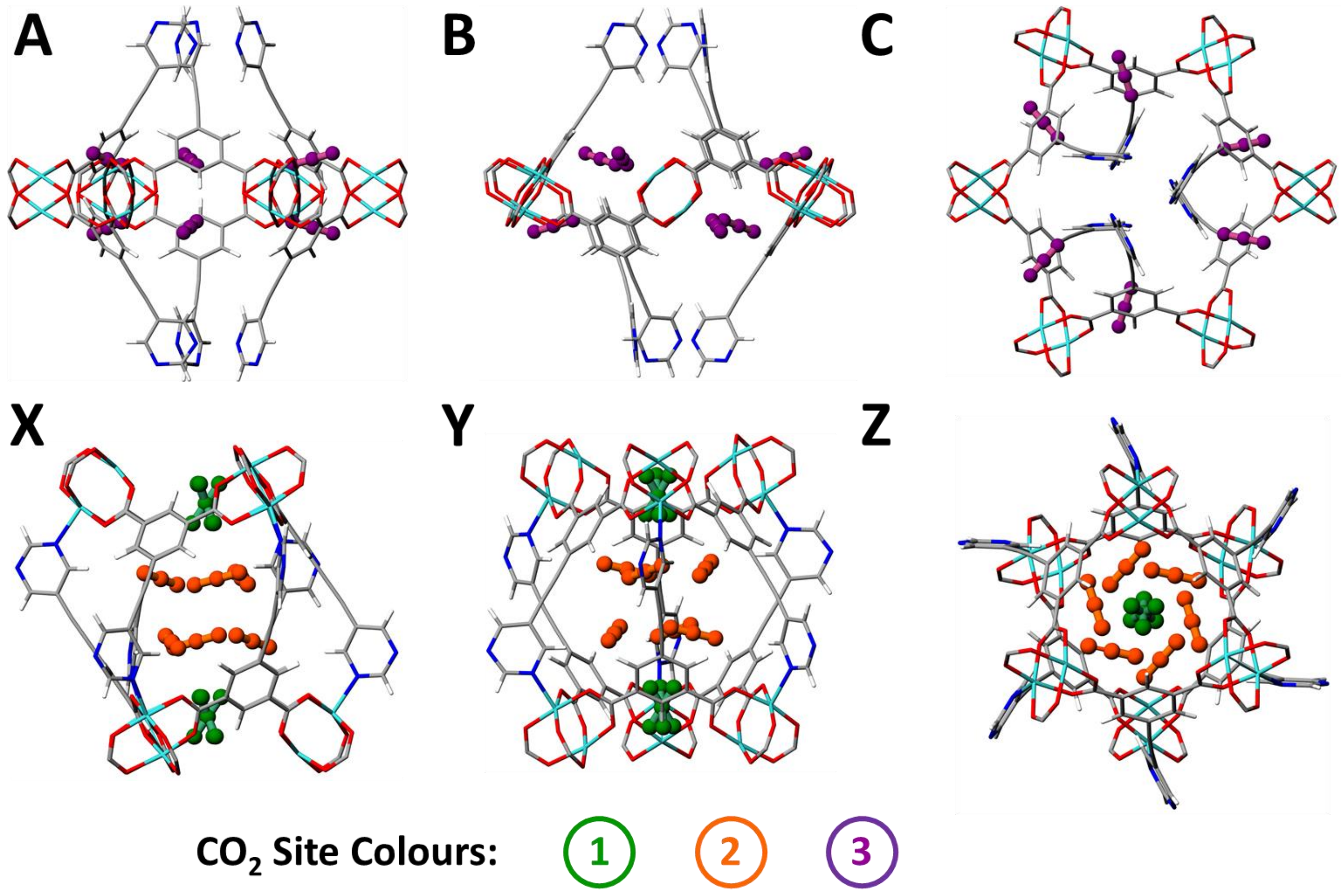

Figure S15. View of all the CO_2 binding sites in MFM-127 loaded with 1.2 CO_2 per Cu(II) centre by refinement of NPD data. (A), (B) and (C) are views of large cage **A** along the *a*, *b* and *c* axes, respectively. (X), (Y) and (Z) are views of small cage **B** along *a*, *b* and *c* axes, respectively. Colours: C, grey; H, white; O, red; N, blue; Cu, teal.

Table S17. Occupancies, multiplicities, positions and short contacts for guest CD_4 sites determined from refinement of NPD data of MFM-127 loaded with 1.0 CD_4 per Cu(II) centre. Intramolecular distances quoted to highest level of precision supported by uncertainty.

**CD_4 Site**	**Multiplicity**	**Chemical Occupancy**	**Sum (occ. x multi.)**	**Cage Location**	**Local Contacts (Å)**	**Shortest Alkyne Interaction (Å)**
**1**	24	1.00	24.000	B	CD_4 **1**...H-C(isophthal) 2.93(1)	n/a
**2**	12	0.359	4.308	B	CD_4 **2**...H-C(pyrimid) 3.26(3)	4.39(1)
**3**	18	0.163	2.934	WALL	CD_4 **3**...H-C(isophthal) 2.61(2)	n/a
**4**	6	0.047	0.282	WALL	CD_4 **4**...CD_4 **3** 3.37(4)	4.82(6)
**5**	16	0.035	0.560	A	CD_4 **5**...C(isophthal) 4.15(6)	5.17(2)

*WALL indicates guest sites located in the void space between cages **A** and **B.**

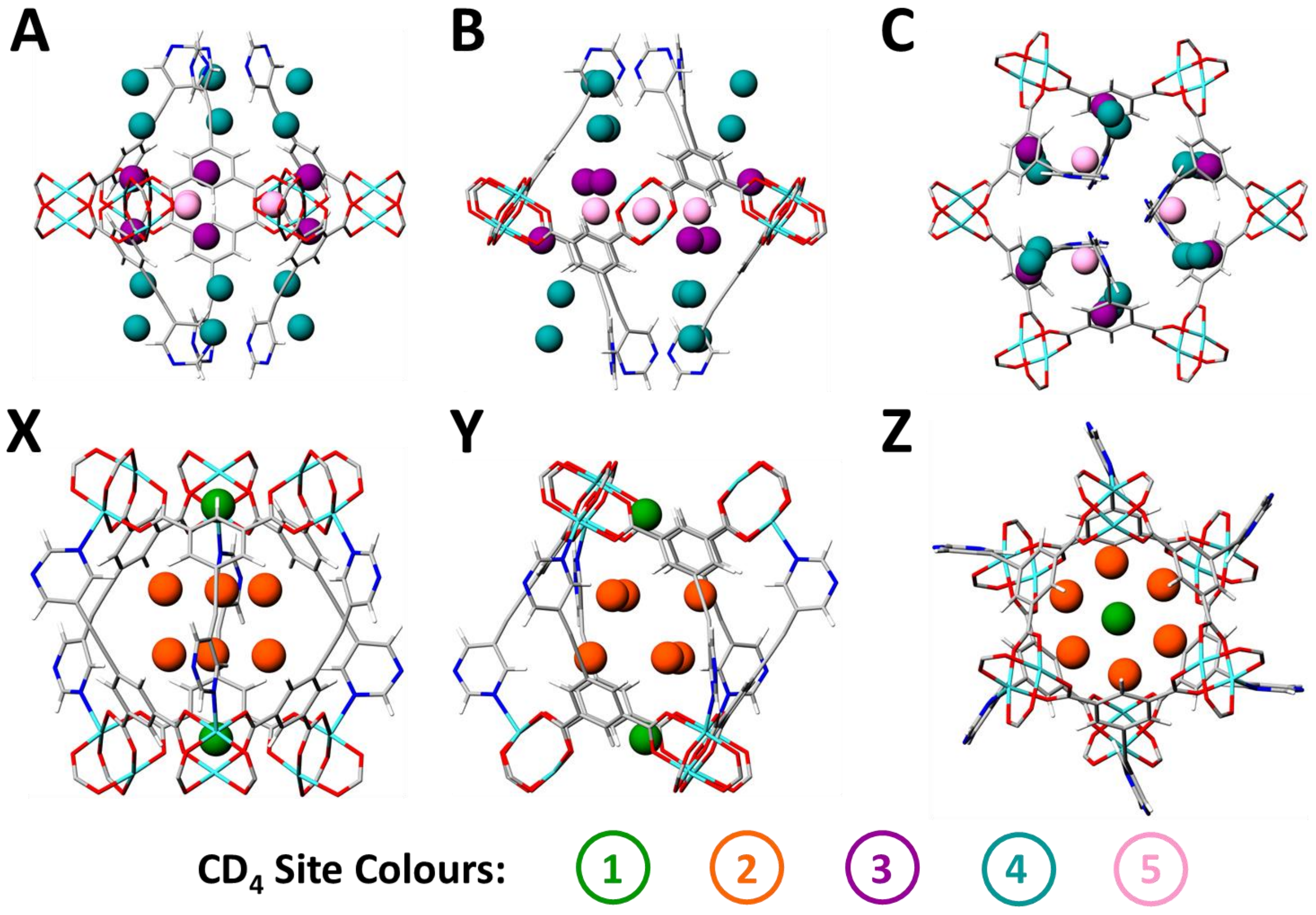

Figure S16. View of all the CD_4 binding sites in MFM-127 loaded with 1.0 CD_4 per Cu(II) centre by refinement of NPD data. (A), (B) and (C) are views of large cage **A** along the *a*, *b* and *c* axes, respectively. (X), (Y) and (Z) are views of small cage **B** along *a*, *b* and *c* axes, respectively. Colours: C, grey; H, white; O, red; N, blue; Cu, teal.

5. Inelastic Neutron Spectroscopy of MFM-126

INS data were collected on the TOSCA beamline at ISIS Muon and Neutron facility.[9] TOSCA is a general purpose inelastic neutron spectrometer which can cover the whole range of molecular vibrations from 0-4000 cm^{-1}. The instrument comprises of 130 ^3He detectors in the forward and backscattering geometry located 17 m downstream of a 300 K Gd poisoned water moderator. A temperature of 7 K was maintained during data collection by two He closed cycle refrigerators. MFM-126 (~1.5 g) was loaded from acetone into a 11 mm diameter vanadium can, sealed with Indium wire and outgassed at 10^{-6} mbar at 393 K for three days to remove any trace guest molecules. After placing the sample into a He cooled cryostat INS data of the bare framework were collected at 7 K. A loading of 1.0 CO_2 per Cu was dosed volumetrically, from a calibrated volume, at room temperature to ensure sufficient mobility of the guest species and gradually cooled to 7 K to allow for the guest species to fully adsorb into MFM-126, with no condensation elsewhere in the system. INS data of 1.0 CO_2/Cu of MFM-126 were collected at 7 K.

DFT Calculations and modelling of the INS spectra

Vibrational frequencies and polarization vectors were calculated using CP2K,[10] based on the mixed Gaussian and plane-wave scheme[11] and the Quickstep module.[12] The calculation used molecularly optimized Double-Zeta-Valence plus Polarization (DZVP) basis set,[13] Goedecker-Teter-Hutter pseudopotentials,[14] and the Perdew-Burke-Ernzerhof (PBE) exchange correlation functional.[15] The plane-wave energy cutoff was 400 Ry. The DFT-D3 level correction for dispersion interactions, as implemented by Grimme *et al*,[16] was applied, with a cutoff distance of 15 Å. The calculation was performed on Gamma point only, with no symmetry constraint. Structural optimization was performed using the Broyden-Fletcher-Goldfarb-Shannon (BFGS) optimizer, until the maximum force is below 0.00045 Ry/Bohr (0.011 eV/Å). Finite displacement method was used for the phonon calculation, with incremental displacement of 0.01 Bohr (0.0053 Å). The INS spectrum was then simulated using the OClimax software.[17]

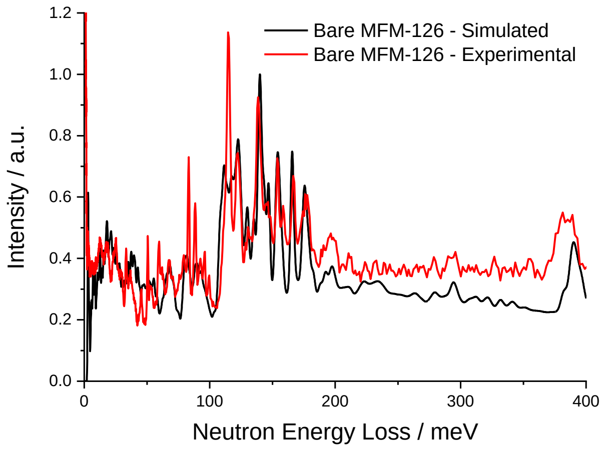

Figure S17. Simulated (black) and experimentally collected (red) INS spectra of bare MFM-126.

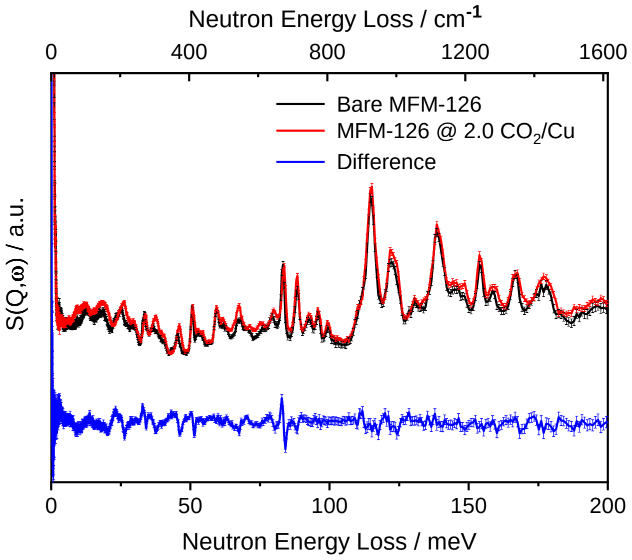

Figure S18. INS spectra for bare MFM-126 (black), CO_2-loaded MFM-126 (red) and the difference INS spectrum (blue).

6. Calculation of isosteric heats of adsorption (Q_{st} values)

Virial analysis of the gas adsorption data was used to determine the isosteric heats of adsorption.

Virial Method 1

This analysis can be performed in two methods. The first method is shown in equations **(I)** and **(II)**.

$$\ln\left(\frac{n}{p}\right) = a_0 + a_1 n + a_2 n^2 + a_3 n^3 + a_4 n^4 + \ldots \qquad \textbf{(I)}$$

where n is the quantity adsorbed at pressure p and a_0, a_1, etc. are virial coefficients. a_0 describes the adsorbate-adsorbent interactions and a_1 describes adsorbate-adsorbate interactions. Under the conditions of low surface coverage, the higher terms (a_2, etc.) can be neglected. The isosteric heat of adsorption at zero coverage is determined *via* equation **(II)**.

$$\delta a_0 = R Q_{st}^{n=0} \delta\left(\frac{1}{T}\right) \qquad \textbf{(II)}$$

Virial Method 2

Alternatively, a virial-type expression can be used to use to perform a global fitting of gas adsorption data at different temperatures *via* equation **(III)**.

$$\ln(p) = \ln(n) + \frac{1}{T}\sum_{i=0}^{m} a_i n^i + \sum_{j=0}^{n} b_j n^j \quad \textbf{(III)}$$

Where n is the quantity adsorbed at pressure p, T is temperature, a_i and b_i are temperature independent virial coefficients and m and n determine the number of terms to adequately describe the isotherm. The resulting virial coefficients, a_i through a_m,were used to calculate enthalpies of adsorption *via* equation **(IV)**.

$$Q_{st} = -R\sum_{i=0}^{m} a_i n^i \quad \textbf{(IV)}$$

R is the universal gas constant (8.314 J^{-1} K^{-1} mol^{-1}).

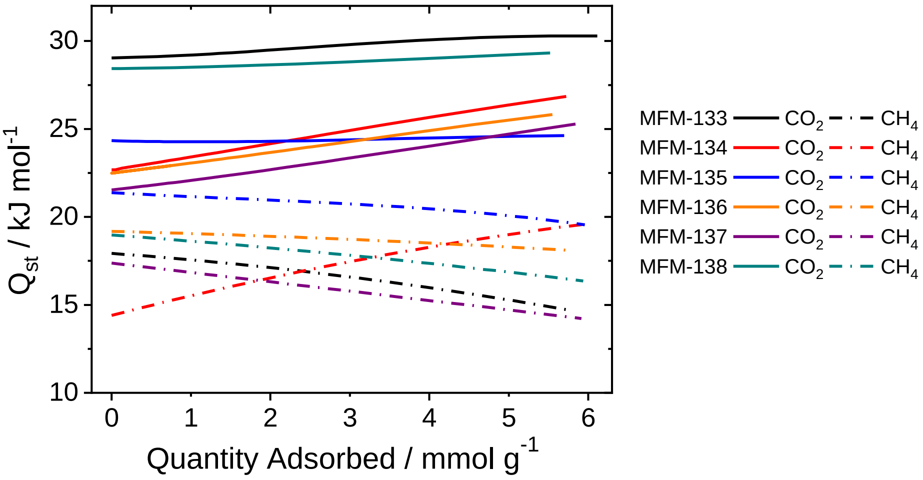

Figure S19. Isosteric heats of adsorption as a function of CO_2 and CH_4 loading in MFM-126-128 and MFM-136-138. All adsorption enthalpies are calculated using virial type 2 methods *via* global fittings.

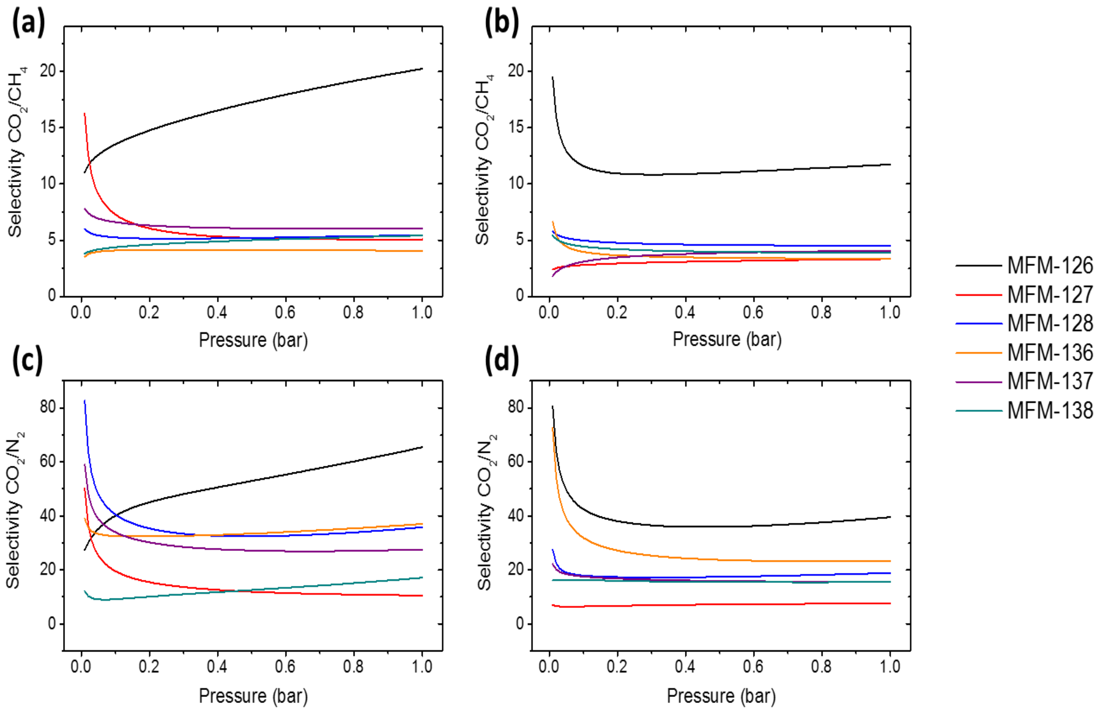

Figure S20. Selectivity values for MFM-126-128 and MFM-136-138 for equimolar mixtures of CO_2/CH_4 at a) 273 K and b) 298 K. Selectivity values for MFM-126 –128 and MFM-136–138 for CO_2/N_2 (15:85) at c) 273 K and d) 298 K.

7. Selectivity Values for CO_2/CH_4 and CO_2/N_2 Binary Mixtures

Table S18. Selectivity values for CO_2/CH_4 binary mixtures at stated compositions, temperatures and pressures.

Compound	S_{CO_2/CH_4}	$CO_2:CH_4$	Temperature / K	Pressure / bar	Reference
MFM-126	20.2	1:1	273 K	1 bar	This work
NJU-Bai8	40.8	n/a*	273 K	<0.15 bar	18
NJU-Bai7	14.1	n/a*	273 K	<0.15 bar	18
MOF-505@GO	8.6	1:1	298 K	1 bar	19
MOF-505	7.6	1:1	298 K	1 bar	19
$[Cu(bpy\text{-}1)_2(SiF_6)]$	10.5	1:1	298 K	1 bar	20
$[Cu(bpy\text{-}2)_2(SiF_6)]$	8.3	1:1	298 K	1 bar	20
MPM-1-TIFSIX	20.3	1:1	298 K	1 bar	21
SIFSIX-2-Cu-i	33	1:1	298 K	1 bar	22
CAU-1	28	n/a*	273 K	<0.15 bar	23
ZJU-15a	6.4	1:1	273 K	1 bar	24
Ni-MOF-74	30	1:1	298 K	1 bar	25
Fe(II)-MOF-74	20.2	1:1	298 K	1 bar	26

*calculated using Henry's law based on data <0.15 bar

Table S19. Selectivity values for CO_2/N_2 binary mixtures at stated compositions, temperatures and pressures.

Compound	S_{CO_2/N_2}	$CO_2:N_2$	Temperature / K	Pressure / bar	Reference
MFM-126	65.4	15:85	273 K	1.0 bar	This work
NJU-Bai8	111	n/a*	273 K	<0.15	18
NJU-Bai7	97.1	n/a*	273 K	<0.15	18
MOF-505@GO	37.2	15:85	298	1.0	19
MOF-505	27.8	15:85	298	1.0	19
MPM-1-TIFSIX	47.1	10:90	298	1.0	21
SIFSIX-2-Cu-i	140	10:90	298 K	1.0	22
CAU-1	101	n/a*	273	<0.15	23
Fe(II)-MOF-74	83.5	50:50	298	1.0	26
HKUST-1	103	50:50	273	1.0	27
2GrO@HKUST-1	186	50:50	273	1.0	27
Mg-MOF-74	148	5:95	323 K	1.0	28

*calculated using Henry's law based on data <0.15 bar

Calculation of Selectivity Values using Ideal Adsorbed Solution Theory

Selectivity values were calculated using the Ideal Adsorbed Solution Theory (IAST) method from single component isotherms. The N_2, CO_2 and CH_4 adsorption isotherms were initially fitted with the dual site Langmuir-Freundlich (DLSF) model (**V**).

$$q = q_{sat,1}\frac{b_1P^{1/n_1}}{1+b_1P^{1/n_1}} + q_{sat,2}\frac{b_2P^{1/n_2}}{1+b_2P^{1/n_2}} \qquad \textbf{(V)}$$

Where q is the amount adsorbed (mmol g^{-1}) at gas pressure P (bar), $q_{sat,i}$ is the saturation capacity (mmol g^{-1}) at sites i, b_i is the affinity coefficients of sites i, and $\boldsymbol{n_1}$is the ideal homogenous surface derivation.

After these fitting parameters were determined IAST was used to predict the mixture adsorption isotherms and subsequently calculate the selectivity values, $\boldsymbol{S_{A/B}}$ (**VI**), for binary mixtures.

$$S_{A/B} = \frac{x_A/x_B}{y_A/y_B} \qquad \textbf{(VI)}$$

Where $\boldsymbol{x_i}$ is the molar fraction of the adsorbed species and y_i is the molar fraction in the gas-phase.

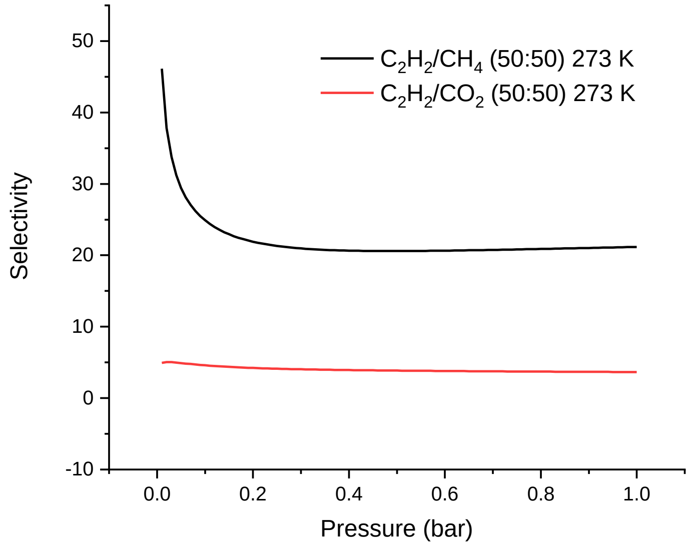

Figure S20. Selectivity values for MFM-127 of equimolar mixtures of C_2H_2/CO_2 and C_2H_2/CH_4 at 273 K.

8. Breakthrough Experiments

To evaluate the performance of MFM-126 in the selective adsorption of CO_2, breakthrough experiments were performed using a Hiden Isochema Automated Breakthrough Analyzer with integrated mass spectrometer. A column packed with MFM-126 (0.95 g) was heated at 393 K under a flow of He (100 mL min^{-1}) overnight before cooling to 298 K. Breakthrough experiments were conducted using N_2/CO_2 (85:15) and CH_4/CO_2 (50:50) which were flowed over the packed bed with a total flow rate of 10 mL min^{-1} at 298 K and 1.0 bar. After breakthrough of both components, desorption was conducted under a flow of He at room temperature to regenerate the column. Dimensionless breakthrough plots were calculated with the following parameters: bed diameter, d, (7 mm), bed length, L, (120 mm), flow rate (10 mL min^{-1}), bed volume (5 mL), sample mass (0.95 g), sample framework density (1.3 g cm^{-3}). The sample occupies a volume of 0.73 mL (assuming 100% purity and no framework collapse), and thus the fractional porosity of the fixed bed, ε, is calculated to be 0.853. The superficial gas velocity, u, at the entrance of the bed corresponds to 4.33e^{-2} m s^{-1}. The characteristic contact time between the gas and MFM-126, εL/u = 23.6 s. The dimensionless time, τ, was obtained by dividing the actual time, t, by the contact time between the gas and the MFM-126 sample, εL/u, i.e. τ = tu/εL.

References

S1 O. Benson, I. Da Silva, S. P. Argent, R. Cabot, M. Savage, H. G. W. Godfrey, Y. Yan, S. F. Parker, P. Manuel, M. J. Lennox, T. Mitra, T. L. Easun, W. Lewis, A. J. Blake, E. Besley, S. Yang and M. Schröder, *J. Am. Chem. Soc*., 2016, **138**, 14828-14831.

S2 H. Nowell, S. A. Barnett, K. E. Christensen, S. J. Teat and D. R. Allan, *J. Synchrotron Radiat*., 2012, **19**, 435-441

S3 CrysAlisPRO, **2014**, Oxford Diffraction /Agilent Technologies UK Ltd, Yarnton, England.

S4 G. M. Sheldrick, Acta Cryst, 2008, **A64**, 112-122.

S5 G. M. Sheldrick, *Acta Crystallogr. Sect. C*, 2014, **C71**, 3-8.

S6 A. Spek, *J. Appl. Cryst*., 2003, **36**, 7-13.

S7 L. C. Chapon, P. Manuel, P. G. Radaelli, C. Benson, L. Perrott, S. Ansell, N. J. Rhodes, D. Raspino, D. Duxbury, E. Spill and J. Norris, *Neutron News*, 2011, **22**, 22-25.

S8 TOPAS-Academic, 2016, Coelho Software, Brisbane, Australia.

S9 S. F. Parker, F. Fernandez-Alonso, A. J. Ramirez-Cuesta, J. Tomkinson, S. Rudic, R. S. Pinna, G. Gorini and J. F. Castañon, *J. Phys. Conf. Ser*., 2014, **554**, 12003.

S10 J. Hutter, M. Iannuzzi, F. Schiffmann and J. VandeVondele, Wiley *Interdiscip. Rev. Comput. Mol. Sci*., 2014, **4**, 15-25.

S11 B. G. Lippert and J. H. and M. Parrinello, *Mol. Phys*., 1997, **92**, 477-488.

S12 J. VandeVondele, M. Krack, F. Mohamed, M. Parrinello, T. Chassaing and J. Hutter, *Comput. Phys. Commun*., 2005, **167**, 103–128.

S13 J. VandeVondele and J. Hutter, *J. Chem. Phys*., 2007, **127**, 114105.

S14 S. Goedecker, M. Teter and J. Hutter, *Phys. Rev. B*, 1996, **54**, 1703-1710.

S15 J. P. Perdew, K. Burke and M. Ernzerhof, *Phys. Rev. Lett*., 1996, **77**, 3865-3868.

S16 S. Grimme, J. Antony, S. Ehrlich and H. Krieg, *J. Chem. Phys*., 2010, **132**, 154104.

S17 A. J. Ramirez-Cuesta, *Comput. Phys. Commun*., 2004, **157**, 226-238.

S18 L. Du, Z. Lu, K. Zheng, J. Wang, X. Zheng, Y. Pan, X. You and J. Bai, *J. Am. Chem. Soc*., 2013, **135**, 562-565.

S19 Y. Chen, D. Lv, J. Wu, J. Xiao, H. Xi, Q. Xia and Z. Li, *Chem. Eng. J*., 2017, **308**, 1065-1072.

S20 S. D. Burd, S. Ma, J. A. Perman, B. J. Sikora, R. Q. Snurr, P. K. Thallapally, J. Tian, L. Wojtas and M. J. Zaworotko, *J. Am. Chem. Soc.*, 2012, **134**, 3663-3666.

S21 P. S. Nugent, V. Lou Rhodus, T. Pham, K. Forrest, L. Wojtas, B. Space and M. J. Zaworotko, *J. Am. Chem. Soc.*, 2013, **135**, 10950-10953.

S22 P. Nugent, T. Pham, K. McLaughlin, P. A. Georgiev, W. Lohstroh, J. P. Embs, M. J. Zaworotko, B. Space and J. Eckert, *J. Mater. Chem. A*, 2014, **2**, 13884.

S23 X. Si, C. Jiao, F. Li, J. Zhang, S. Wang, S. Liu, Z. Li, L. Sun, F. Xu, Z. Gabelica and C. Schick, *Energy Environ. Sci*., 2011, **4**, 4522.

S24 X. Duan, Y. Zhou, R. Lv, B. Yu, H. Chen, Z. Ji, Y. Cui, Y. Yang and G. Qian, *J. Solid State Chem*., 2018, **260**, 31-33.

S25 D.-L. Chen, H. Shang, W. Zhu and R. Krishna, *Chem. Eng. Sci*., 2015, **124**, 109-117.

S26 W. Lou, J. Yang, L. Li and J. Li, *J. Solid State Chem*., 2014, **213**, 224-228.

S27 F. Xu, Y. Yu, J. Yan, Q. Xia, H. Wang, J. Li and Z. Li, *Chem. Eng. J*., 2016, **303**, 231-237.

S28 J. A. Mason, K. Sumida, Z. R. Herm, R. Krishna and J. R. Long, *Energy Environ. Sci*., 2011, **4**, 3030.
